# Personalized neoantigen-pulsed autologous dendritic cells in newly-diagnosed glioblastoma: a phase Ib trial

**DOI:** 10.1038/s41467-026-75066-w

**Published:** 2026-07-04

**Authors:** Yang Zhang, Xiaohan Chi, Kang Liu, Yiwen Ma, Shaoze Zhang, Xiaomin Ma, Shengjun Sun, Yu Zhang, Kangna Zhang, Na Huang, Xudong Cheng, Nan Ji

**Affiliations:** 1 Department of Neurosurgery, Beijing Tiantan Hospital, Capital Medical University, Beijing, China; 2 ZSky BioTech Co. LTD, Beijing, China; 3 Department of Radiology, Beijing Tiantan Hospital, Capital Medical University, Beijing, China; 4 Department of Neuroradiology, Beijing Neurosurgical Institute, Beijing, China

**Keywords:** Cancer immunotherapy, CNS cancer

## Abstract

Newly-diagnosed glioblastoma (nGBM) remains a significant unmet need with limited therapeutic options. Here, we present the results of a single-arm, open-label phase 1b trial on neoantigen-pulsed autologous dendritic cells (ZSNeo-DC) as an adjuvant treatment for nGBM. The primary endpoint was safety, as assessed by the incidence and severity of adverse events (AEs). Eleven patients with nGBM received intradermal administration of ZSNeo-DC following surgery and standard radio-chemotherapy. Treatment was well tolerated, with AEs predominantly grade 1 or 2; only two grade-1/2 febrile AEs were recorded as treatment-related. Secondary endpoint analysis showed that median progression-free survival (PFS) was 16.2 months, median overall survival (OS) was not reached, and 12-month OS rate was 100% from surgery. A range of 1.9- to 284.7-fold (median: 7.2-fold) and 0.7- to 507.0-fold (median: 10.3-fold) increase of peripheral neoantigen-specific immune response (NSIR) was observed respectively after the 3^rd^ and 5^th^ vaccination. Following vaccination, both the activated-to-exhausted ratio of effector/central memory T cells and the clonal evenness of the T cell receptor repertoire increased significantly in peripheral blood; these parameters positively correlated with NSIR and PFS. Overall, ZSNeo-DC exhibits a favorable safety profile and preliminary efficacy signals in nGBM, supporting phase 2 evaluation in expanded cohorts. Trial number: NCT04968366.

## Abstract (web summary)

Newly diagnosed glioblastoma (nGBM) remains a major unmet clinical need with limited effective therapeutic options. This phase 1b trial reports safety and preliminary efficacy outcomes of nGBM patients treated with neoantigen-pulsed autologous dendritic cell vaccination.

## Introduction

As the most common and aggressive primary brain malignancy in adults, glioblastoma multiforme (GBM) remains a significant unmet clinical challenge with dismal survival, owing to its poor response to current therapies^1^. The standard-of-care (SOC) paradigm for GBM treatment involves maximal surgical resection followed by radiation therapy combined with temozolomide (TMZ)-based concurrent and adjuvant chemotherapy, with consideration of tumor-treating fields (TTFs) for patients who have no evidence of progression following radiochemotherapy^2,3^. However, these standard therapies provide only modest survival benefits, yielding a median overall survival of 15-20 months and a 5-year survival rate not exceeding 10% from the date of surgery. These survival data have remained largely unchanged over the past two decades^1,4^. Therefore, there is an urgent need for novel and safe therapeutic options.

As master antigen-presenting cells, dendritic cells (DCs) play a pivotal role in eliciting anti-tumor T cell immunity. Therefore, DCs are natural vaccine candidates against tumors, and extensive efforts have been dedicated to developing DC-based vaccines against various malignancies like GBMs. These endeavors have yielded encouraging therapeutic efficacy in treating this deadly brain cancer^5–10^. Among these vaccines, DCvax, a personalized DC vaccine pulsed with autologous tumor cell lysate, has showed survival benefit in a phase 3 trial^5^. Nevertheless, despite the promising results of DCvax and other tumor-lysate DC vaccines^5,8,11,12^, the uncertainty surrounding tumor antigens poses challenges in precise monitoring of immune response and raises concerns about off-tumor toxicity^13^. Other approaches for developing DC vaccine involve pulsing DCs with tumor-associated antigens (TAAs)^14–17^ or viral antigens (VAs)^18,19^, also yielded promising early-clinical results. However, these approaches are limited by the inherent weak antigenicity of TAAs or the risk of antigen evasion due to the fixed antigens loaded.

Neoantigens, also termed as Tumor-specific Antigens (TSAs), mainly arise from somatic mutations in tumor cells. Given that they bypass central and peripheral tolerance mechanisms^20^, and are absent in normal tissues, they theoretically induce a more robust and/or specific immune response compared to other tumor antigens. Advances in next-generation sequencing along with multi-omics analysis have enabled fast and accurate neoantigen prediction^21,22^. Based on them, several studies on neoantigen peptide vaccines have demonstrated promising results in triggering intense tumor-specific T cell responses and thus extending survival in glioma patients^23,24^. Clinical response to peptide vaccines relies on patients’ own dendritic cells for antigen presentation and subsequent activation of tumor-killing T cells. However, several studies have reported glioma-induced DC dysfunction^25,26^, which may result in poor therapeutic response.

To address these issues, we developed a personalized DC vaccine (ZSNeo-DC), which was loaded with multiple neoantigen peptides to generate a robust and tumor-specific immune response while avoiding immune evasion. The vaccine was also modified with PD-L1 blockade and CD40 agonism to counter possible DC dysfunctions. Preclinical experiments have demonstrated that the vaccine improves neoantigen-specific T cell activation and elicits a strong tumor-killing effect (see below). Therefore, we initiated a phase 1b study to evaluate its safety and preliminary efficacy, when it was administered alongside the SOC therapy in newly-diagnosed GBM (nGBM) patients.

## Result

### Baseline characteristics of enrolled patients

**Fig. 1:**
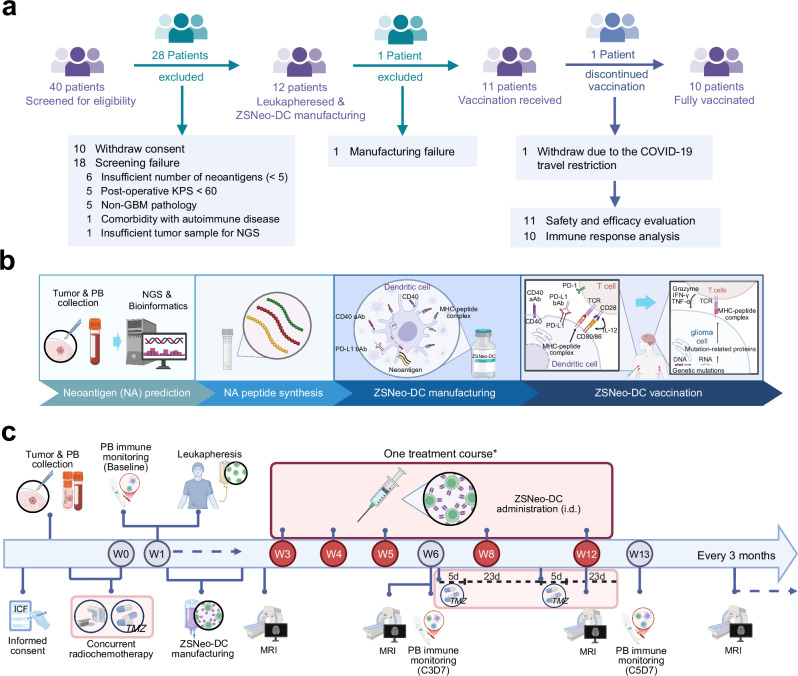
ZSNeo-DC vaccine design and study overview. **a** CONSORT flow diagram depicts patient enrollment and treatment. **b** Schematic overview of ZSNeo-DC vaccine design and generation. **c** Schematic overview of study design. The figures are created in BioRender.com. NGS, next generation sequencing; PB, peripheral blood; CTL, cytotoxic T lymphocyte; i.d., intradermal injection; C3D7, day 7 after the third vaccination; C5D7, day 7 after the fifth vaccination; TMZ, temozolomide; KPS, Karnofsky Performance Status. * Five injection cycles constitute one treatment course; up to three additional injection cycles are allowed within a single course and multiple treatment courses are permitted.

To investigate safety and preliminary efficacy of ZSNeo-DC, we initiated a phase 1b trial on the treatment of nGBMs using the vaccine as an adjuvant therapy, in combination with SOC post-operative radiochemotherapy^27^. Between July 2021 and September 2023, a total of 40 patients were screened for eligibility, and 12 were eligible and received leukapheresis for DC manufacturing (Fig. 1a). Eligible patients were aged 18 to 75 years, with nGBM diagnosis confirmed by histopathological exams and genetic analysis. Eligibility also requires ≥ 90% resection extent of contrast-enhanced lesions, ≥60 Karnofsky Performance Status (KPS) score and adequate organ functions and hematologic parameters. Due to one patient failed in the DC generation, 11 patients were eventually treated with the vaccine, and were included in the safety analysis and efficacy evaluation. For the treated patients, a median 8 neoantigen peptides were used for DC generation, and these peptides covered various gene mutations and diverse subtypes of HLA-I or II molecules (Supplementary dataset 1).

Baseline characteristics of all the treated patients are presented in Table 1. The median age of these patients was 55 years (range: 34–67 years), and median KPS was 70 (range: 60–90) at baseline. All patients achieved gross-total or near-total resection of contrast-enhanced tumors, leading to no or minimal residual tumor after operation. All patients completed one treatment course, comprising 5 to 6 doses, except one patient (R04) having 4 doses due to travel restriction by the Covid-19 pandemic. In combination with the vaccination, all patients received the standard Stupp’s regimen of radio-chemotherapy^27^, and one patient (R05) concurrently had the TTF therapy, which has also constituted a standard treatment for nGBM^2^. Noteworthily, genetic analysis showed that all the tumors were isocitrate dehydrogenase (IDH) wild type, 54.5% (6/11) of patients were O^6^-methylguanine DNA methyltransferase (MGMT) unmethylated, and 63.6% (7/11) had telomerase reverse transcriptase (TERT) mutations, indicating that most patients belonged to the poor prognosis subtype in GBMs^28^.

**Table 1:** Individual Patient Characteristics

Patient ID	Age (ys)	Sex	KPS score	Pre-operative tumor volume (cm^3^)	Post-operative tumor volume (cm^3^)	EOR (%)	Number of vaccination	Post-surgery treatment	Molecular Pathology			Post-progression treatment
									IDH	MGMT	TERT	
R01	38	M	60	148.392	0	100	6	RT/ TMZ	WT	UM	WT	-
R02	59	M	80	37.050	0	100	5	RT/ TMZ	WT	UM	M	Anti-PD-1^b^, BEV
R03	34	F	90	99.222	0	100	5	RT/ TMZ	WT	UM	WT	-
R04	67	M	80	54.738	1.034	98.11	4	RT/TMZ	WT	NA^a^	WT	TMZ
R05	47	F	80	52.364	1.482	97.17	5	RT/ TMZ /TTF	WT	UM	M	-
R06	65	M	70	28.410	0	100	5	RT/ TMZ	WT	UM	M	Anti-PD-1^b^, BEV, VP-16, CBDCA, CDDP, PTX
R07	49	M	70	44.616	0	100	5	RT/ TMZ	WT	ML	M	-
R08	58	M	80	73.500	0	100	5	RT/ TMZ	WT	UM	M	Anti-PD-1^b^, BEV
R09	55	F	60	16.555	0	100	5	RT/ TMZ	WT	ML	M	BEV
R10	55	F	60	54.018	1.047	98.06	5	RT/ TMZ	WT	UM	WT	Anti-PD-1^b^, BEV, Anlo
R11	66	F	70	64.220	0	100	5	RT/ TMZ	WT	ML	M	CDDP, TMZ, BEV

a. Insufficient tumor tissue for methylation testing. b. Anti-PD-1 antibodies included pembrolizumab (R02 and R08) and sintilimab (R06 and R10). KPS, Karnofsky Performance Status; EOR, extent of resection; RT, radiotherapy; TMZ, Temozolomide; TTF, tumor treating field; IDH, isocitrate dehydrogenase; MGMT, O^6^ -methylguanine DNA methyltransferase; TERT, telomerase reverse transcriptase; WT, wild-type; M, mutation; UM, unmethylated; ML, methylated; BEV, bevacizumab; VP-16, etoposide; CBDCA, carboplatin; CDDP, cisplatin; PTX, paclitaxel; Anlo, anlotinib.

There was no significant difference in baseline characteristics between the treated and consent-withdrawn patients, whereas those patients excluded due to a low number of predicted neoantigens exhibited better baseline KPS and lower tumor mutation burden than the treated patients (Supplementary dataset 2). Therefore, the low tumor mutation burden may partially contribute to ineligibility for ZSNeo-DC therapy.

### ZSNeo-DC vaccine and therapeutic regimen

The ZSNeo-DC vaccine adopted a DC design that combined a neoantigen-prediction algorithm based on tumor tissue/blood multi-omic data (Fig. S1), and immune cell engineering for DC modification to enhance the abilities in activating neoantigen-specific T cells (Fig. 1b and Figs. S2–3). The therapeutic regimen and corresponding immune monitoring and follow-up plan are summarized in Fig. 1c. Briefly, enrolled patients first underwent neurosurgical resection, followed by blood and tumor tissue sampling for genomic and transcriptomic profiling for neoantigen prediction. Leukapheresis for ZSNeo-DC generation was performed after concurrent chemoradiotherapy. Based on experience from benchmark DC trials in nGBMs^5,10^, the standard vaccination schedule comprised three weekly doses for immune induction and at least two booster doses for immune consolidation. Specifically, ZSNeo-DC (1 × 10^7^ DCs/dose) was administered intradermally near inguinal and axillary lymph nodes at Weeks 3, 4, 5, 8, and 12 post-chemoradiotherapy (five-dose standard course). Up to three additional doses (Weeks 16, 20, 24) were permitted if DCs were available. Multiple courses were allowed. Lymphodepletion was not used before vaccination. PBMC samples collected at leukapheresis (Baseline), 7 days after the third (C3D7) and fifth doses (C5D7), were utilized for immune response evaluation (Fig. 1c). A more detailed study protocol is shown in Methods.

### Safety and adverse events

The primary objective of the study is to assess safety of ZSNeo-DC. Treatment-emergent adverse events (TEAEs) observed during the DLT observation period (from the first shot to eight weeks after the last shot) are listed in Table 2. The most recorded TEAEs were lymphocytopenia (63.6%, 7/11), hypertriglyceridemia (54.5%, 6/11), leukopenia (45.5%, 5/11) and hypercholesterolemia (45.5%, 5/11). Three cases of hematologic AEs (2 cases of lymphocytopenia and one case of leukopenia), two cases of hydrocephalus, one case each for seizure, bronchial infection and spinal fracture were reported as grade 3 TEAEs. No grade 4 or 5 TEAEs were reported. All these AEs were deemed unrelated to the vaccine; instead, some were likely attributed to TMZ-based chemotherapy or radiotherapy^29–31^. Only two cases of grade 1/2 fever were recorded as the vaccine related. Therefore, the ZSNeo-DC vaccine was quite safe and well-tolerated, when combined with the SOC treatment for nGBMs.

**Table 2:** Adverse events recorded during the DLT observation period

Event	Grade1-2 n (%)	Grade 3 n (%)	Grade 4-5 n (%)
**General disorders and administration site conditions**			
Fever	2 (18.2%)	0	0
**Nervous system disorders**			
Seizure	2 (18.2%)	1 (9.1%)	0
Hydrocephalus	0	2 (18.2%)	0
**Blood and lymphatic system disorders**			
Lymphocytopenia	5 (45.5%)	2 (18.2%)	0
Leukopenia	4 (36.4%)	1 (9.1%)	0
Anaemia	3 (27.3%)	0	0
**Gastrointestinal disorders**			
Constipation	2 (18.2%)	0	0
**Hepatobiliary disorders**			
GGT increased	1 (9.1%)	0	0
**Infections and infestations**			
Bronchial infection	0	1 (9.1%)	0
**Injury, poisoning and procedural complications**			
Spinal fracture	0	1 (9.1%)	0
**Metabolism and nutrition disorders**			
Hyperchloremia	4 (36.4%)	0	0
Hypercholesterolemia	5 (45.5%)	0	0
Hyperglycemia	3 (27.3%)	0	0
Hyperphosphatemia	3 (27.3%)	0	0
Hypertriglyceridemia	6 (54.5%)	0	0
Hyperuricemia	1 (9.1%)	0	0
Hypokalemia	2 (18.2%)	0	0
Hyponatremia	2 (18.2%)	0	0
Hypoproteinemia	2 (18.2%)	0	0
**Psychiatric disorders**			
Delirium	1 (9.1%)	0	0
Insomnia	1 (9.1%)	0	0
**Vascular disorders**			
Hypertension	1 (9.1%)	0	0
**Investigations**			
Blood lactate dehydrogenase increased	1 (9.1%)	0	0

### Clinical efficacy

**Fig. 2:**
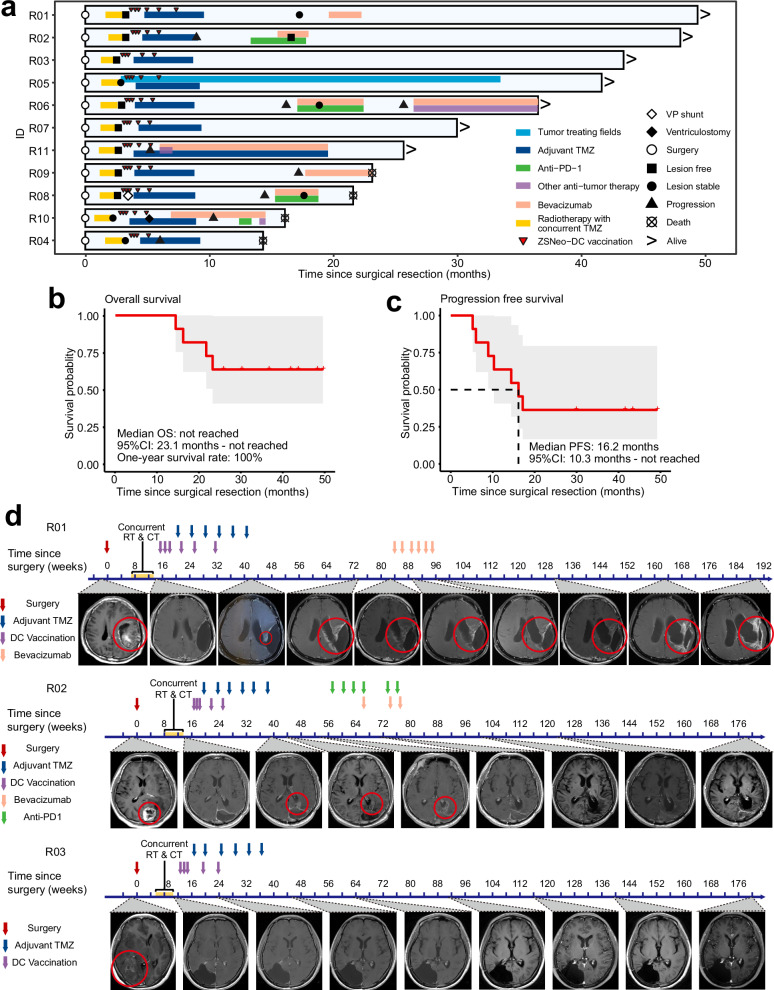
Clinical efficacy. **a** Swimmer plot illustrates therapies and survival of patients receiving the planned doses of ZSNeo-DC vaccine (*n* = 11). VP shunt, ventriculoperitoneal shunt. **b, c** Kaplan–Meier curves illustrate **b** overall survival (OS) and **c** progression free survival (PFS) from the date of tumor resection (*n* = 11). Shaded bands indicate the 95% confidence intervals of the Kaplan–Meier survival estimates. **d** Representative MRI images for subjects R01, R02, and R03 are presented. Week numbers are indicated relative to the date of tumor resection. Tumor regions are circled in red. Key treatment events are annotated with arrowheads: surgery (red), ZSNeo-DC therapy (purple), initiation of each cycle of adjuvant temozolomide (TMZ) chemotherapy (blue), bevacizumab administration (light pink), and anti-PD-1 therapy (green). Periods of concurrent radiotherapy (RT) and chemotherapy (CT) are highlighted with yellow bars.

One secondary objective was to evaluate preliminary clinical efficacy of ZSNeo-DC in treating nGBMs by assessing survival outcomes. Therapeutic results and major events are summarized in Fig. 2a. As of the cut-off analysis date (October 31, 2025), the median follow-up time duration was 41.6 months (95% CI: 30.0 months-not reached). Kaplan-Meier analysis revealed that the median PFS was 16.2 months (95% CI: 10.3 months-not reached), median OS was not reached (95% CI: 23.1 months-not reached), and the 12-month OS rate was 100% from surgery (Fig. 2b, c).

Patient R01, with a tumor located in the left frontal lobe, was the first patient treated in the trial and received six doses of ZSNeo-DC (Fig. 2d). While the patient initially maintained physical activity at baseline, contrast-enhancement emerged surrounding the resection cavity on MRI at Week 42 (10 weeks after the last vaccination) and enlarged significantly at Week 74, accompanied by reduced walking distance and slightly decreased strength in the right lower limb compared to baseline. Without any treatment, the enhancement shrank on the subsequent MRI nine weeks later. Therefore, we suspected tumor pseudo-progression, a phenomenon commonly reported in anti-glioma vaccine studies^5,32^, and administered six cycles of bevacizumab to control pseudo-progression and related symptoms. Consequently, the patient maintained good physical activity with stable enhancement on MRI until the cut-off date, leading to a PFS time as long as 49.4 months (Fig. 2a). Patient R02, with a tumor in the left occipital lobe, was administered five ZSNeo-DC injections (Fig. 2d). However, a new contrast-enhancing lesion was detected on MRI at Week 39, with progressive enlargement at Week 44 and 53, and was consequently reported as disease progression. Post-progression therapy was administered at the physician’s discretion. Based on the reported potential synergy between DC vaccines and anti-PD-1/PD-L1 therapies^23,33,34^, the patient was treated with pembrolizumab in addition to standard bevacizumab therapy for recurrent GBM. Subsequent MRI showed resolution of the recurrent lesion. The patient remained lesion-free at the time of report. Patient R03, with a tumor located in the right temporal lobe, maintained a lesion-free status for 43.4 months (Fig. 2a, d) after receiving five ZSNeo-DC shots.

### Immunological efficacy

**Fig. 3:**
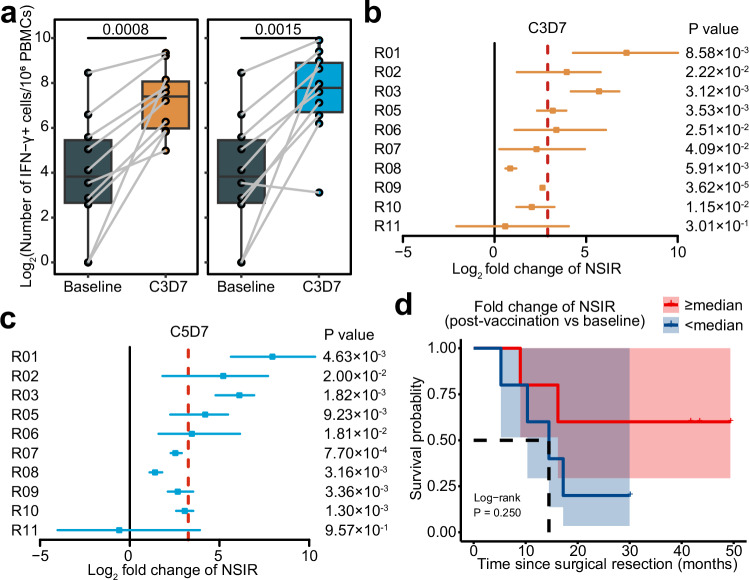
Immunological efficacy. **a** Neoantigen-specific immune response (NSIR) were assessed at baseline, day 7 after the third (C3D7) and fifth vaccinations (C5D7) using an IFN-γ secreting enzyme-linked immunospot (ELISPOT) assay. NSIR was quantified as the number of IFN-γ-secreting peripheral blood mononuclear cells (PBMCs) stimulated with neoantigen peptide-pulsed DCs, after subtracting the response to blank DCs. Data were log_2_(x + 1) transformed for analysis. Paired two-sided t-tests were applied to assess statistical significance, based on normality testing (*n* = 10). The median and interquartile range with whiskers extending to the minimum and maximum values are depicted. No adjustment for multiple comparisons was applied. **b**, **c** Forest plots showing the individual fold changes of NSIR at **b** C3D7 and **c** C5D7 relative to baseline for each patient. Each row represents one biologically independent patient (*n* = 10). For each patient and time point, ELISPOT assays were performed using three technical replicates. Data is presented as the model estimate of the post-pre-change on a log_2_(x + 1) scale with 95% confidence intervals (CI). The central point represents the median fold change. Red dashed lines indicate the median fold change. Paired two-sided t-tests were used for significance testing. No adjustment for multiple comparisons was applied. **d** Kaplan–Meier curve illustrates difference of progression-free survival between patients with NSIR above (≥) (*n* = 5) and below (<) (*n* = 5) the median fold change after vaccination. Shaded bands indicate the 95% confidence intervals of the Kaplan–Meier survival estimates. Statistical significance was assessed by the log-rank test.

Another secondary objective was to assess immunological efficacy of ZSNeo-DC. Ten patients completed the full treatment course, and peripheral blood samples were collected according to the schedule for the evaluation of immunological efficacy. Utilizing an IFN-γ secreting ELISPOT assay, we evaluated the neoantigen-specific immune response (NSIR), which was elicited by DCs pulsed by neoantigen peptides (Methods), in the PBMC samples collected at baseline, C3D7 and C5D7, respectively (Fig. 1b). Overall, a median increase of 7.2-fold (range: 1.9 to 284.7 folds) and 10.3-fold (range: 0.7 to 507.0 folds) were observed after the 3^rd^ and 5^th^ vaccination respectively compared with the baseline levels; 9 patients (90%) exhibited a positive immunological response and showed a significant increase in NSIR post-vaccination (Fig. 3a-c). Moreover, patients with a high immune response (above the median level) tended to have extended PFS than those with a low response (below the median level) (Fig. 3d), demonstrating a link between survival benefits and NSIR.

### Peripheral T cell phenotype alterations

**Fig. 4:**
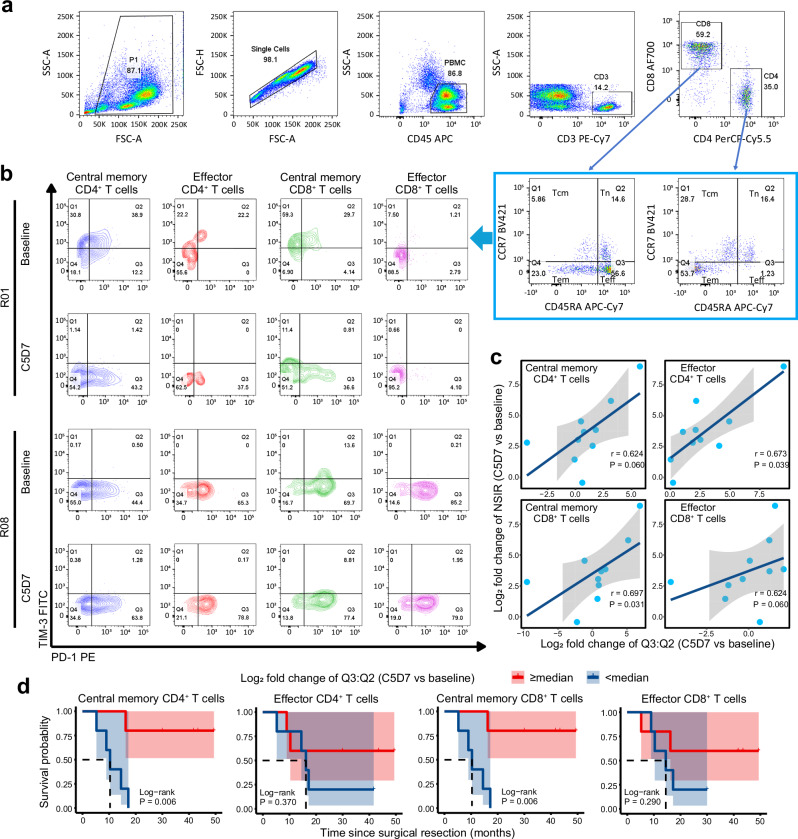
Peripheral T cell phenotype alterations. **a** Gating strategy used for T cell differentiation analysis. Naive (Tn), central memory (Tcm), effector memory (Tem), and effector (Teff) T cells were distinguished based on CD45RA and CCR7 expression. **b** Representative flow cytometry plots from patients R01 (long-PFS responder) and R08 (short-PFS non-responder) show dynamic changes in CD4^+^/CD8^+^ T cell central memory and effector populations from baseline to C5D7. Q1, TIM-3^+^ PD-1^-^; Q2, TIM-3^+^ PD-1^+^; Q3, TIM-3^-^ PD-1^+^; Q4, TIM-3^-^ PD-1^-^. **c** Correlations between the fold changes (C5D7 vs baseline) of NSIR and the activation-to-exhaustion ratio (Q3:Q2) in CD4^+^/CD8^+^ T cell central memory and effector populations (*n* = 10). Fold changes are shown on a log_2_(x + 1) scale. Associations were assessed using two-sided Spearman’s rank correlation. The solid line represents the fitted linear regression line for visualization, and the grey shaded band indicates the 95% confidence interval of the fitted line. No adjustment for multiple comparisons was applied. **d** Kaplan–Meier curves illustrate difference of progression-free survival between patients with a high level (≥ median) (*n* = 5) of change in activation-to-exhaustion ratio and those with a low level (<median) (*n* = 5). Shaded bands indicate the 95% confidence intervals of the Kaplan–Meier survival estimates. Statistical significance was assessed by the log-rank test.

We next explored T cell phenotype alterations in peripheral blood after vaccination. In both effector (CD45RA^+^CCR7^-^) and central memory (CM, CD45RA^-^CCR7^+^) T cell populations, we observed an increase in activated (PD-1^+^TIM3^-^) CD4^+^ T cells while a decrease or a trend toward decrease in exhausted (PD-1^+^TIM3^+^) CD4^+^ T cells at C5D7 compared to baseline levels (Fig. 4a, b and Fig. S4). A similar trend was also observed in CD8^+^ effector/CM T cells at C5D7 in patients with a high NSIR (Fig. 4a, b and Fig. S4). These findings indicate that ZSNeo-DC therapy may promote phenotypic conversion from exhaustion to activation in peripheral effector/CM T cell populations. Accordingly, we utilized the activated-to-exhausted ratios in these T cell populations to quantify this phenotypic alteration, and found that they positively correlated with NSIR (Fig. 4c) and extended PFS (Fig. 4d), supporting the clinical relevance of peripheral T cell phenotype remodeling following ZSNeo-DC vaccination.

### Peripheral T cell receptor repertoire changes

**Fig. 5:**
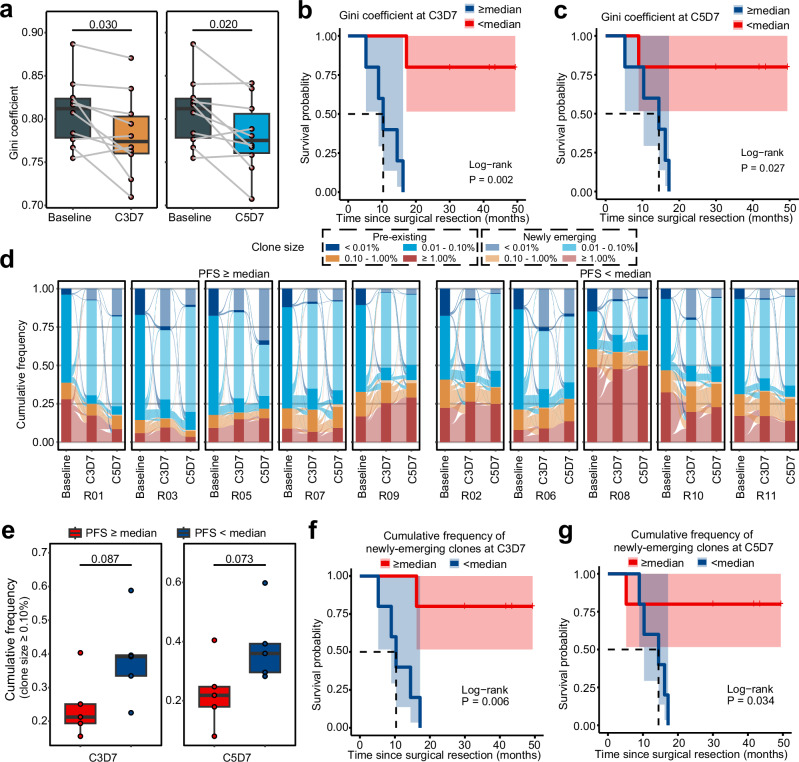
Peripheral T cell receptor repertoire changes. **a** Gini coefficient of peripheral T cell receptor (TCR) repertoire is compared among time-points. Paired two-sided t-tests were applied to assess statistical significance (*n* = 10). The median and interquartile range with whiskers extending to the minimum and maximum values are depicted. No adjustment for multiple comparisons was applied. **b, c** Kaplan–Meier curves illustrating difference of PFS between patients with Gini coefficient above (≥) (*n* = 5) and below (<) (*n* = 5) the median level at **b** C3D7 and **c** C5D7. Shaded bands indicate the 95% confidence intervals of the Kaplan–Meier survival estimates. Statistical significance was assessed by the log-rank test. **d** Sankey diagram showing the clonal frequency transitions (categorized by clone size and pre-existing or newly emerging clones) for individual patients (grouped by PFS longer than the median level or not) across sequential time points. **e** Cumulativ**e** frequencies of large-sized clones (≥ 0.1%) are compared between patients with PFS above (≥) (*n* = 5) and below (<) (*n* = 5) the median level at the indicated time-points. Two-sided t-tests or Wilcoxon rank-sum tests were applied to assess statistical significance, based on normality testing. The median and interquartile range with whiskers extending to the minimum and maximum values are depicted. No adjustment for multiple comparisons was applied. **f, g** Kaplan–Meier curve illustrating difference of PFS between patients with the cumulative frequencies of TCR clones, newly emerging at **f** C3D7 or **g** C5D7, above (≥) (*n* = 5) and below (<) (*n* = 5) the median level. Shaded bands indicate the 95% confidence intervals of the Kaplan–Meier survival estimates. Statistical significance was assessed by the log-rank test.

We subsequently utilized bulk beta-chain T cell receptor (TCR) sequencing to track TCR repertoire alteration in peripheral blood after ZSNeo-DC vaccination. Overall, we observed a significant decrease in the Gini coefficient post-vaccination (Fig. 5a), while other indicators reflecting TCR repertoire diversity showed no significant changes (Fig. S5). Moreover, the Gini coefficient was significantly correlated with patient survival, with patients in the low coefficient score group (<median level) showing extended PFS compared to those in the high score group (≥ median level) (Fig. 5b, c). And Gini coefficient scores were inversely related to NSIR levels (Fig. S6a, b). The Gini coefficient is commonly used to evaluate the evenness of TCR clone frequencies^35,36^, with lower coefficient scores indicating more even or balanced clonal landscape. These findings indicate that ZSNeo-DC vaccination induces an increase of peripheral TCR repertoire evenness, which reflects a favorable response to the therapy.

An increase of TCR repertoire evenness indicates either a loss of large-size clones or an expansion of small-size clones in TCR clone space^35,36^. We observed a dramatic difference in TCR clone space profiles between patients in the long and short PFS groups (Fig. 5d), with the long-PFS patients showing a trend toward lower proportions of large-size clones (≥ 0.1%) post-vaccination than those with a short PFS (Fig. 5e). The proportions of large-size clones also showed a trend toward inverse correlation with NSIR levels (Fig. S6c, d). These findings suggest the disproportion of large-size clones may contribute to the difference in TCR repertoire evenness between the long and short PFS patients, and thus the divergent response to ZSNeo-DC therapy. However, we further observed that the majority of large-size clones at baseline were retained post-vaccination (at C3D7 or C5D7) (Fig. 5d), indicating that the disproportion of large-size clones is not due to a loss of these clones post-vaccination, but rather from the differential expansion of small-size clones between the long and short PFS patients. Notably, the small-sized clones (<0.1%) post-vaccination were largely occupied by newly emerging clones (Fig. 5d). Therefore, the differential expansion of newly emerging clones post-vaccination may explain the difference in TCR repertoire evenness and divergent response to ZSNeo-DC vaccination. Supporting this view, increased levels of newly emerging clones were correlated with extended PFS (Fig. 5f, g) and better immune response (Fig. S6e, f). Together, we observed a dramatic increase in TCR repertoire evenness following vaccination. The increased levels of TCR repertoire evenness, primarily driven by the high expansion of newly emerging clones post-vaccination, predict a better response to ZSNeo-DC therapy.

## Discussion

In this phase 1b pilot study, we evaluated the safety and preliminary efficacy of a neoantigen-loaded DC vaccine--ZSNeo-DC, when it was combined with the SOC therapy for nGBMs. We observed that ZSNeo-DC therapy was well-tolerated. The median PFS was 16.2 months (95% CI: 10.3 months-not reached), and median OS had not been reached after a median follow-up of 41.6 months. The survival data shows an efficacy signal of ZSNeo-DC therapy, as compared with the median PFS of 7–10 months commonly reported in nGBM patients receiving SOC treatment^5,37,38^. With respect to immune response, the vaccine elicited a median increase of 7.23-fold and 10.33-fold in NSIR respectively at C3D7 and C5D7, promoted phenotypic conversion from exhaustion to activation in peripheral effector/CM T cells and induced a dramatic increase in peripheral TCR repertoire evenness. These observations suggest the potential immune activity of the vaccine. Together, these exploratory findings provide a rationale for further investigation of ZSNeo-DC in nGBMs.

ZSNeo-DC demonstrated a favorable safety profile, with only two grade 1/2 febrile TRAEs and no injection site reactions or fatigue commonly reported with DC therapy^10,39^. Consistent with previous reports^6,7,39^, the most common AE was lymphocytopenia, recorded in 63.6% (7/11) of participants and likely related to standard therapy. Notably, no serious TRAEs were observed in this cohort, aligning with the extremely low incidence (0–0.3%) reported in large cohorts of DC therapy^6,10,40^. Together, the safety profile of ZSNeo-DC is comparable to that of these landmark studies, underscoring the safety advantage of DC therapy for GBM treatment.

Many clinical trials have been conducted utilizing DC vaccines to treat malignant gliomas^5–8,10,14,18,41,42^. Current DC-vaccine strategies for gliomas include pulsing DCs with tumor lysate, TAAs and VAs. Neoantigen-loaded DC vaccines are theorized to elicit a stronger and more specific anti-tumor immune response. This study showed the promise of the DC platform to provide neoantigen-based immunotherapies against gliomas. Nevertheless, a recent trial by Latzer P et al. involving 70 patients with nGBM also demonstrated encouraging clinical outcomes and a favorable safety profile with personalized neoantigen peptide vaccines^24^, suggesting that comparable clinical benefits may be achieved with alternative neoantigen vaccine strategies. Besides the neoantigen design, ZSNeo-DC manufacturing adopts cytokine-driven maturation and utilizes PD-L1 blockade and CD40 agonism to enhance DC functions; these procedures are not routinely used in the manufacturing of other DC vaccines. The impact of these manufacturing variations on clinical and immune responses warrants further investigations.

Consistent with previous findings in tumor vaccine studies^5,11,12^, we observed a divergent response to the vaccine, with a fraction of patients showing a more than 10-fold increase in NSIR while few exhibiting modest or even no change after vaccination (Fig. 3b, c). Consequently, patients with strong NSIR tended to have extended survival than those with weak response (Fig. 3d). Many factors are reported to link with this divergency of response, including genetic mutations^43^, mutation burden^44^, tumor immune microenvironment features^23^, residual tumor size^45^, and systemic immune status^8^. In our small cohort, aside from age (a common risk factor for survival in GBM patients^46^), variables such as residual tumor size, driver gene mutations, tumor mutation burden, and local tumor immune status did not significantly impact survival (Supplementary dataset 3). However, a higher activated-to-exhausted ratio in peripheral effector/CM T cells (Fig. 4c, d), as well as a more balanced clonal space (Fig. 5b, c and Fig. S6a, b) or newly emerging clones (Fig. 5f, g and Fig. S6e, f) in the peripheral T cell repertoire, correlated with better response to ZSNeo-DC therapy. By contrast, patients without these favorable immunological profiles exhibited survival outcomes, on some occasions, comparable to those reported for the standard Stupp’s regimen^5,37,38^, suggesting potential resistance to ZSNeo-DC therapy. These findings indicate that these peripheral T cell alterations may mediate the immune reaction and thus clinical response to the vaccine.

Pseudo-progression is a common phenomenon in immunotherapies against gliomas and can confound image evaluation^5,32,47,48^. It has been reportedly associated with favorable response to anti-glioma vaccines^32^. Similarly, we observed this phenomenon in patient R01. The patient showed post-vaccination contrast-enhancement, which persisted stably up to the cut-off date (Fig. 2d). Notably, the patient achieved the longest PFS time of 49.4 months within this cohort and exhibited the highest level of NSIR, with a greater than 200-fold increase compared to baseline. Therefore, this phenomenon may also suggest effective immune and clinical response to the therapy, corroborating with previous studies^32,47^.

GBM is notorious for its “cold” and inhibitory tumor microenvironment (TME)^49,50^, leading to its resistance to immune checkpoint blockade (ICB) therapies in several large-scale trials^47^. Therefore, laborious efforts have been exerted to improve GBM’s response to ICB. Reshaping the TME into a “hot” one through tumor vaccines is a promising way^23,51^. Therefore, there are several reports showing potential synergy between DC vaccines and anti-PD1/PD-L1 therapies^33,34^, as well as ongoing trials examining their safety and efficacy when administered concurrently in GBM^52,53^. In this study, four patients received ICB therapies after tumor progression (Fig. 2a). Among them, two had stable disease afterward, and one even showed durable complete remission. Though the sample-size is small, this observation reveals an efficacy signal of ICB therapies for GBMs, implying that ZSNeo-DC vaccination may reverse GBMs’ resistance to ICB therapies. Further exploration into combinatory strategies of ICB and ZSNeo-DC therapies is needed.

As a pilot trial, this study had a limited sample size, precluding statistically significant correlations between NSIR and clinical outcomes despite observable trends. Preliminary efficacy signals may be confounded by selection bias and post-progression management variability, requiring validation in randomized controlled trials. Additionally, the 33.3% exclusion rate due to insufficient neoantigens may limit broader applicability of ZSNeo-DC therapy. Furthermore, budgetary constraints precluded monitoring of neoantigen-specific T cells during treatment. The quantity and quality of these cells-especially intratumorally-are critical for therapeutic efficacy and may underlie the observed alterations in peripheral T cell phenotypes and TCR repertoire.

Overall, ZSNeo-DC vaccine, administered with the SOC treatment, was well tolerated; preliminary data suggest possible survival benefits in this small, selected nGBM cohort, warranting validation in larger trials.

## Methods

### Study design, eligibility and vaccination procedure

This is a single-center, single-arm phase 1b study to determine the safety and preliminary efficacy of ZSNeo-DC for treating nGBMs. The study protocol received approval from the institutional review board and ethics committee of Beijing Tiantan Hospital (KY 2021-021-02), adhering to ethical guidelines. Written informed consent was obtained from all participants in accordance with the principles of the Declaration of Helsinki. This study is registered in ClinicalTrials.gov (NCT04968366; first posted on July 20, 2021), and the clinical study protocol is available within the Supplementary Information.

All patients who sign the informed consent forms are screened for the eligibility assessment. Sex or gender was not considered in the study design. Sex of participants was determined based on self-report. Inclusion criteria include: (1) age between 18 and 75 years; (2) ≥ 90% resection of contrast-enhancing lesions; (3) Karnofsky Performance Status (KPS) score ≥ 60; (4) nGBM confirmed by histological exams and molecular diagnosis; (5) adequate bone marrow, liver, kidney, and cardiac function prior to DC vaccine administration, defined as: absolute white blood cell count ≥ 2.5 × 10^9^ /L; hemoglobin level > 100 g/L; platelet count > 100 × 10^9^ /L; serum alanine transaminase and aspartate transferase levels <2.5 × ULN (Upper Limit of Normal), and serum creatinine level <1.5 × ULN; and (6) ability to understand and sign an informed consent form.

Exclusion criteria include: (1) other uncontrolled malignancies; (2) Carmustine wafer implantation within 6 months; (3) active hepatitis B/C or HIV infection; (4) poorly controlled grade 2-3 hypertension; (5) serious cardiovascular or cerebrovascular diseases; (6) active autoimmune disorders (e.g., hemolytic anemia, psoriasis, rheumatoid arthritis); (7) unstable psychiatric illness; (8) long-term immunosuppressant treatment for patients such as organ transplant recipients; (9) systemic administration of corticosteroids with a dosage exceeding 10 mg prednisone or equivalent for one day within 4 weeks before vaccination; (10) recent thromboembolic events or ongoing anticoagulation therapy; (11) pregnancy, lactation, or planned conception within 2 months post-treatment; (12) active or uncontrolled infections requiring systemic therapy; (13) prior treatment history of antitumor vaccine or gene-modified cell therapy; (14) fewer than five predicted neoantigen peptides; (15) deemed unsuitable by investigators; (16) inability to provide consent or comply with study procedures; or (17) participation in another clinical trial within 4 weeks before enrollment.

After neurosurgical resection, the enrolled patients receive the SOC radio-chemotherapy, following the Stupp’s protocol^27^, that is initiated 4-6 weeks post-surgery. Within 7 days after the end of concurrent radio-chemotherapy, leukapheresis is performed for ZSNeo-DC generation. ZSNeo-DC is administered intradermally at a dose of 1 × 10^7^ DCs per injection, delivered near the inguinal and axillary lymph nodes at Weeks 3, 4, 5, 8, and 12 post-chemoradiotherapy, comprising a total of five doses per treatment course. The dose of 1 × 10^7^ cells^54^ and the dosing scheme^5,10^ were designed primarily based on the kinetics of DC-mediated immune responses and experience from other DC trials, with three weekly induction doses for full expansion of antigen-specific T cells, and two subsequent booster doses for immune consolidation. The timing of the booster doses was set at two weeks after the start of the 5-day TMZ maintenance chemotherapy to further enhance anti-tumor immune responses^24^. If sufficient DCs remain, up to three additional doses (at Weeks 16, 20, and 24 post-chemoradiotherapy) may be administered within the same course. Multiple treatment courses are permitted.

### Outcomes and clinical assessment

The primary objective is to evaluate the safety of ZSNeo-DC, combined with the SOC treatment, for treating nGBM. Safety evaluation is based on recording and grading adverse events (AEs) according to the Common Terminology Criteria for Adverse Events (CTCAE) version 5.0. All AEs are recorded until 8 weeks after the last vaccination. AEs occurring more than 8 weeks after the last vaccination are also recorded if they are considered related to the treatment. Secondary objectives include: (1) immunological efficacy of ZSNeo-DC that is assessed by an interferon-γ secreting enzyme-linked immunospot (ELISPOT) assay at baseline, C3D7, C5D7, and the 7^th^ day after the last injection (if more than 5 injections were given), with a positive immunological response defined as at least 55 antigen-specific spot-forming cells (SFC) per 10^6^ PBMCs or a ≥ 3-fold increase from baseline; (2) preliminary clinical efficacy evaluated by PFS, OS, 1-year survival rate and quality of life assessment (in patients with postoperative measurable lesions, objective response rate, disease control rate and clinical benefit rate were also evaluated); and (3) correlations of immune profiles—immune molecular characteristics and immune cell phenotypes— in peripheral blood and cerebrospinal fluid (CSF) with immunological and clinical efficacy. Given the central role of T cells in antitumor immune response and the scarcity of T cells in CSF, we focused on alterations in T cell phenotypes and repertoire features in peripheral blood in this study.

Clinical assessment is performed within 2 weeks before vaccination, on days 21 and 63, and subsequently every 3 months until disease progression. The immunotherapy Response Assessment in Neuro-Oncology (iRANO) criteria are used to determine the disease status^55^. PFS is defined as the duration from the date of surgery to disease recurrence/progression or death from any cause. OS is defined as the duration from the date of surgery to death from any cause. One-year survival rate is defined as the rate of patients who survive more than one-year since surgery in the efficacy analysis population. Quality of life was assessed using the EORTC QLQ-C30. Clinical data were collected using an electronic data capture (EDC) system (Aidiyan Medical Technology Ltd., version 1.4.3).

### Sample collection and processing

For whole-exome sequencing (WES), HLA typing and RNA sequencing (RNA-seq), peripheral blood was collected by venipuncture into anticoagulant tubes and cryopreserved, whereas tumor specimens obtained by surgical resection or biopsy were stabilized in RNAlater solution and stored at −80 °C. For longitudinal immune monitoring, 15 mL of EDTA-anticoagulated whole blood was collected during leukapheresis (baseline) and on day 7 after the third (C3D7) and fifth vaccinations (C5D7) to isolate peripheral blood mononuclear cells (PBMCs) for ELISPOT assays, flow cytometry analysis, and T-cell receptor sequencing (TCR-seq). Peripheral blood was collected in anticoagulant-treated tubes and processed within 8 h. PBMC extraction and storage followed the standard procedure.

### WES, HLA typing and RNAseq analysis

WES and HLA typing were conducted by Novogene Biotechnology Co., Ltd. Clean reads were aligned to the human reference genome (GRCh37/hg19) using BWA (v0.7.17), and somatic variants were identified with Sentieon TNseq (v202010.01), followed by standard filtering. Variant annotation was performed using ANNOVAR, and mutational signatures were analyzed with SomaticSignatures and deconstructSigs in R (version 4.4.0). Copy number alterations were estimated with DNAcopy and visualized using Circos. Clonal architecture was inferred using PyClone (v0.13.1), with mutations exhibiting a cancer cell fraction (CCF) > 0.8 defined as clonal. Phylogenetic trees were constructed based on mutational profiles using supraHex. HLA haplotypes were predicted from WES blood samples using Polysolver (v4) and HLAminer (v1.4).

For transcriptomic profiling, raw sequencing data underwent quality control using fastp (v0.20.1) to remove adapters and low-quality reads, generating clean data. Cleaned reads were aligned to the human reference genome (GRCh38/hg38) using HISAT2 (v2.1.0). Gene expression was quantified with StringTie (v1.3.4 d) and normalized to transcripts per million (TPM), with genes exhibiting TPM < 1 excluded from further consideration. To assess mutational support in RNA, read counts at variant positions were quantified using bam-readcount, and this information was annotated into the VCF files using vcf-readcount-annotator for integration with neoantigen prediction.

### T-Cell receptor library construction, sequencing and analysis

Genomic DNA was extracted from PBMCs using the MagPure Blood DNA KF Kit (Magen, 5111-02). T-cell receptor beta (TCRβ) repertoire libraries were constructed using the HaploX TRB Immune Repertoire Library Construction Kit (HaploX), following the manufacturer’s instructions. Multiplex PCR was performed with primers targeting the flanking regions of the complementarity-determining region 3 (CDR3) of V and J segments to capture TCRβ diversity. Libraries were sequenced on the Illumina NovaSeq 6000 platform with the NovaSeq 6000 S4 Reagent Kit v1.5 (20028312) and the NovaSeq XP 4-Lane Kit v1.5 (20043131). All sequencing was conducted by HaploX (Shenzhen, China).

Raw sequencing data were processed using Fastp to remove adapter sequences and filter low-quality reads. Cleaned reads were aligned and assembled using MiXCR v4.2.2 for TCR clonotype identification, with annotation of V(D)J gene segments and determination of complementarity-determining region 3 (CDR3) amino acid sequences. Clones with read counts ≥ 3 were retained as high-confidence clones for downstream analyses. Clonal diversity and frequency distribution were quantified using the *immunarch* package, including Gini coefficient, D50, true diversity, Gini-Simpson index, Shannon index, cumulative frequency of top 25 clones, and total number of clones. Clones with a log_2_ fold change ≥ 3 relative to baseline were defined as expanded clones.

### Neoantigen prediction and prioritization

Tumor tissue underwent RNA sequencing for expression quantification and variant calling, while paired tumor tissue and peripheral blood samples were subjected to WES for somatic variant detection. Sequencing data were quality controlled, aligned to the reference genome, and processed for variant annotation. HLA typing was performed in parallel, and identified variants were integrated and analyzed for MHC–peptide binding affinity prediction. Predicted neoantigens were filtered to exclude germline and non-mutated sequences, and prioritized based on binding affinity, expression levels, and variant-specific features for downstream immunogenicity assessment.

### Neoantigen peptide synthesis

Selected neoantigen peptides were synthesized using standard Fmoc solid-phase chemistry and purified by reverse-phase HPLC ( > 90% purity) by GenScript (Nanjing, China) following GMP standard. Peptide identity was confirmed by mass spectrometry, and lyophilized products were stored at -20 °C. For experimental use, peptides were dissolved in sterile water or DMSO to 10-25 mM stock concentrations, with working concentrations of 25 μM for ex vivo assays.

### Generation of ZSNeo-DC

ZSNeo-DC was prepared as previously described (Dendritic cell preparation and preparation method thereof, Patent No.: US12440565B1). Briefly, it was manufactured under strict GMP conditions with multiple quality checkpoints described as follows. Monocytes were first positively selected from leukapheresis products and then underwent purity checking by flow cytometry. Monocytes were subsequently cultured in serum-free medium supplemented with GM-CSF and IL-4 (all from R&D Systems, USA) for generating DCs, with a medium refreshed to maintain cytokine activity. On day 6, immature DCs were harvested, phenotyped with flow cytometry and induced into mature phenotypes with a maturation medium consisting of 5-20 personalized neoantigen peptides, a cytokine cocktail including TNF-α, IL-1β, IL-6, prostaglandin E2 (all from R&D Systems, USA), and poly I:C (Invivogen, USA). During the 24-hour maturation process, the maturation medium was supplemented with a PD-L1 blockade antibody (Atezolizumab) and a CD40 agonist antibody (Cifurtilimab) to enhance the capacity of DC in antigen-presentation and T cell activation. Both antibodies are clinical-grade and have shown overall safety and clinical activity in treating other malignancies (NCT03474289, NCT03711305 and NCT02376699). Finally, DC maturation was assessed by measuring the expression levels of CD80, CD83, CD86 and HLA-DR expression using flow cytometry. Prior to clinical use, each vaccine batch underwent quality control testing, which included assessments of cell viability, migratory capacity, purity, and sterility (Supplementary dataset 4). Only DC products meeting all the release criteria were cryopreserved in aliquots of 1 × 10^7^ cells per dose using controlled-rate freezing.

### Interferon-γ secreting ELISPOT assay

Neoantigen-specific immune response in peripheral blood during vaccination was assessed using an optimized interferon-γ (IFN-γ) ELISPOT protocol (Cellular Technology Limited, USA). Briefly, a total of 2.0–5.0 × 10^5^ freshly thawed PBMCs were plated in duplicate or triplicate with DCs pulsed by neoantigen peptides (DC-pulsed) at a 10:1 ratio (PBMCs: DCs) in serum-free medium. PHA (Dakewe, USA) was used as a positive control at a final concentration of 2.5 μg/mL. Negative controls included the wells added with DMSO (blank control) or DCs without being pulsed with neoantigen peptides (DC-mock control). After 20-hour incubation, plates were developed according to the manufacturer’s protocol with stringent wash steps between antibody incubations. Spot enumeration was performed using an ImmunoSpot® analyzer with advanced image analysis algorithms. Results were normalized to spot-forming units (SFU) per 10^6^ PBMCs. Neoantigen-specific immune response was measured as the number of SFU in DC-pulsed wells minus those in DC-mock control wells.

### Flow cytometry

Multiparametric flow cytometry was performed for T cell phenotype characterization using freshly isolated or thawed PBMCs or fresh peripheral blood samples. For whole blood samples, red blood cell lysis was conducted after staining. Cells were incubated with a pre-mixed antibody cocktail containing CD45, CD3, CD4, CD8, CD45RA, CCR7, CXCR3, CD69, PD-1 and TIM-3 antibodies (all from BioLegend, USA) in staining buffer for 15 minutes at room temperature in the dark, followed by two washes with PBS. Data acquisition was carried out on a BD Lyric flow cytometer. T cell subsets were defined as: naïve (CD45RA^+^CCR7^+^), central memory (CD45RA^-^CCR7^+^), effector memory (CD45RA^-^CCR7^-^), and effector (CD45RA^+^CCR7^-^) populations. Appropriate controls including unstained cells, single-color compensations, and fluorescence-minus-one (FMO) controls were used to establish gating thresholds. All flow cytometry data were analyzed using FlowJo software (version 10.8.1) with standardized gating templates to ensure consistency across experiments. For studies involving multiple batches, batch normalization was applied during data analysis. This optimized protocol incorporates critical quality control measures including daily instrument calibration, comprehensive control samples, and standardized analysis approaches to ensure reproducible and reliable T cell immunophenotyping results. Detailed information of antibodies used for flow cytometry are listed in Supplementary dataset 5.

### Neoantigen-Specific T-cell Cloning and Cytotoxicity Assay

A 21-amino-acid neoantigen peptide (QSQHMTEVVRHCPHHERCSDS) covering the neoantigen epitope was chemically synthesized. Monocytes from healthy donors were used to generate ZSNeo-DCs following the described production process. These DCs were used to stimulate and expand CD8^+^ T lymphocytes from the same donor in vitro. P53^R175H^ T cells and control T cells were generated by co-culture with ZSNeo-DCs pulsed with mutant or blank peptides, respectively.

Briefly, ZSNeo-DCs were cocultured with CD8^+^ T in a ratio of 5:1 (T cells: DCs) in CTL medium. Cells were plated in a 24-well plate (1 × 10^6^ enriched T cells per well in 1 mL of CTL medium). IL-21 (30 ng/mL) was added to the cell mixture on day 0 for promoting CTL activation and expansion. Medium and cytokines were replenished on days 3, 5 and 7, with IL-7 and IL-15 were used at a concentration of 5 ng/mL on days 3 and 5, and at 10 ng/mL on day 7. All cytokines were purchased from Peprotech. On day 10, CTLs were harvested for phenotypic studies by flow cytometry, including assessment of activation markers CD69 and CXCR3 and T-cell differentiation profiles. Neoantigen-specific T-cell reactivity was confirmed using IFN-γ ELISPOT following overnight peptide restimulation.

After confirming the composition of T-cell subsets in the CTL by flow cytometry, tumor cell lines which had been correctly identified for the neoantigen epitope were used as target cells to assess the specific killing effect of P53^R175H^-CTL cells on the target cells. KLE tumor cells (CTCC-400-0001, Zhejiang meisen cells technology co., ltd, China) expressing the P53^R175H^ neoantigen (abbreviated as KLE^mut^) were used as target cells, while CFPAC-1 tumor cells (CTCC-400-0058, Zhejiang meisen cells technology co., ltd, China) expressing wild-type p53 served as negative control cells (abbreviated as CFPAC-1^wt^). P53^R175H^ T cells or control T cells were co-cultured with the two cell types at effector-to-target (E:T) ratios of 2.5:1, 5:1, and 10:1. The RTCA cell analyzer was used to monitor target cell death and calculate the killing activity of T cells against tumor cells.

For cytotoxicity assays, tumor cell lines (KLE^mut^ or CFPAC-1^wt^) were used as mutant target or negative control target cells. Real-time cytolytic activity was quantified using an impedance-based xCELLigence RTCA system. Target cells were seeded into 96-well microplates pre-coated with gold microelectrodes and allowed to adhere for 24 h. P53^R175H^-specific T cells or control T cells were then added at effector-to-target (E:T) ratios of 2.5:1, 5:1 and 10:1, with three technical replicate wells per condition. Cellular impedance, expressed as the Cell Index, was continuously monitored for 6 h. Cytolytic activity was calculated based on changes in the normalized Cell Index relative to control wells.

For morphological validation of tumor-cell killing, KLE^mut^ and CFPAC-1^wt^ cells were seeded into culture plates and allowed to adhere for 24 h. CTLs were then added at the indicated E:T ratios together with the apoptosis indicator Incucyte® Caspase-3/7 Green. After 12 h of co-culture, fluorescence imaging was performed using the Incucyte® Live-Cell Analysis System. Green fluorescent signals localized within tumor cells were interpreted as caspase-3/7 activation, indicating apoptosis-associated tumor-cell killing.

### Statistical methods

Demographics and relevant participant features were summarized using descriptive statistics. Kaplan-Meier curves were used to estimate the median survival time and survival probability. 95% CIs were calculated with the log method. Two-sided t-tests or Wilcoxon rank-sum tests were employed for comparisons between two groups, while ANOVA or Kruskal-Wallis H tests were utilized for significance tests between multifactorial groups based on tests for normality. Correlation analysis was performed using Spearman’s test. Two-tailed *P* < 0.05 was considered statistically significant. All statistical analyses were conducted using R (version 4.4.0).

### Reporting summary

Further information on research design is available in the Nature Portfolio Reporting Summary linked to this article.

## Supplementary information

Supplementary information

Description of Additional Supplementary Files

Supplementary Dataset 1

Supplementary Dataset 2

Supplementary Dataset 3

Supplementary Dataset 4

Supplementary Dataset 5

Reporting Summary

Transparent Peer Review file

## Data Availability

The raw WES, RNA-seq and TCR-seq data generated in this study have been deposited in the Genome Sequence Archive for Human (GSA-Human) of the China National Center for Bioinformation under accession code HRA014059 [NGDC - GSA for Human]. These human sequencing data are available under restricted access because they contain patient-derived genomic information and are subject to patient privacy, informed consent and institutional ethics requirements. Access can be obtained by applying through the GSA-Human data access system and completing the required data access agreement. Where additional approval is required, requests should be directed to the corresponding authors, Dr. Nan Ji (jinan@mail.ccmu.edu.cn) and Dr. Xudong Cheng (chengxudong@zskybio.com). Requests will be reviewed within approximately 30 business days, and approved access will be granted for the duration specified in the data access agreement. The clinical study protocol is available within the Supplementary Information. The raw patient-level clinical data are protected and are not publicly available due to privacy and consent restrictions. De-identified clinical data, processed immunological data and other data underlying the results reported in this study are provided in the Source Data files. Source data are provided with this paper.
