# Supplementary material for: Personalized neoantigen-pulsed autologous dendritic cells in newly-diagnosed glioblastoma: a phase Ib trial
Source: Nat Commun. 2026 Jul 4;17:8302. doi: 10.1038/s41467-026-75066-w (PMC13469091; doi:10.1038/s41467-026-75066-w)
Supplement: Supplementary file 1 [file 41467_2026_75066_MOESM1_ESM.pdf]

Supplemetary Information

Personalized neoantigen-pulsed autologous dendritic cells in newly-diagnosed glioblastoma: a phase Ib trial.

Yang Zhang[1#], Xiaohan Chi[1#], Kang Liu[1], Yiwen Ma[1], Shaoze Zhang[1], Xiaomin Ma[2], Shengjun Sun[3,4], Yu Zhang[1], Kangna Zhang[1], Na Huang[1], Xudong Cheng[2*], Nan Ji[1*]

Affiliation

1. Department of Neurosurgery, Beijing Tiantan Hospital, Capital Medical University, Beijing, China
2. ZSky BioTech Co. LTD, Beijing, China
3. Department of Radiology, Beijing Tiantan Hospital, Capital Medical University, Beijing, China
4. Department of Neuroradiology, Beijing Neurosurgical Institute, Beijing, China

# These authors contributed equally: Yang Zhang, Xiaohan Chi.

* These authors jointly supervised this work: Xudong Cheng, Nan Ji.

Corresponding Authors: Nan Ji, M.D., Department of Neurosurgery, Beijing Tiantan Hospital, Capital Medical University, Beijing, China (jinan@mail.ccmu.edu.cn); Xudong Cheng, Ph.D., ZSky BioTech Co. LTD, Beijing, China (chengxudong@zskybio.com);

Supplementary Figure 1-6

Supplementary Note 2: Study Protocol

Figure S1

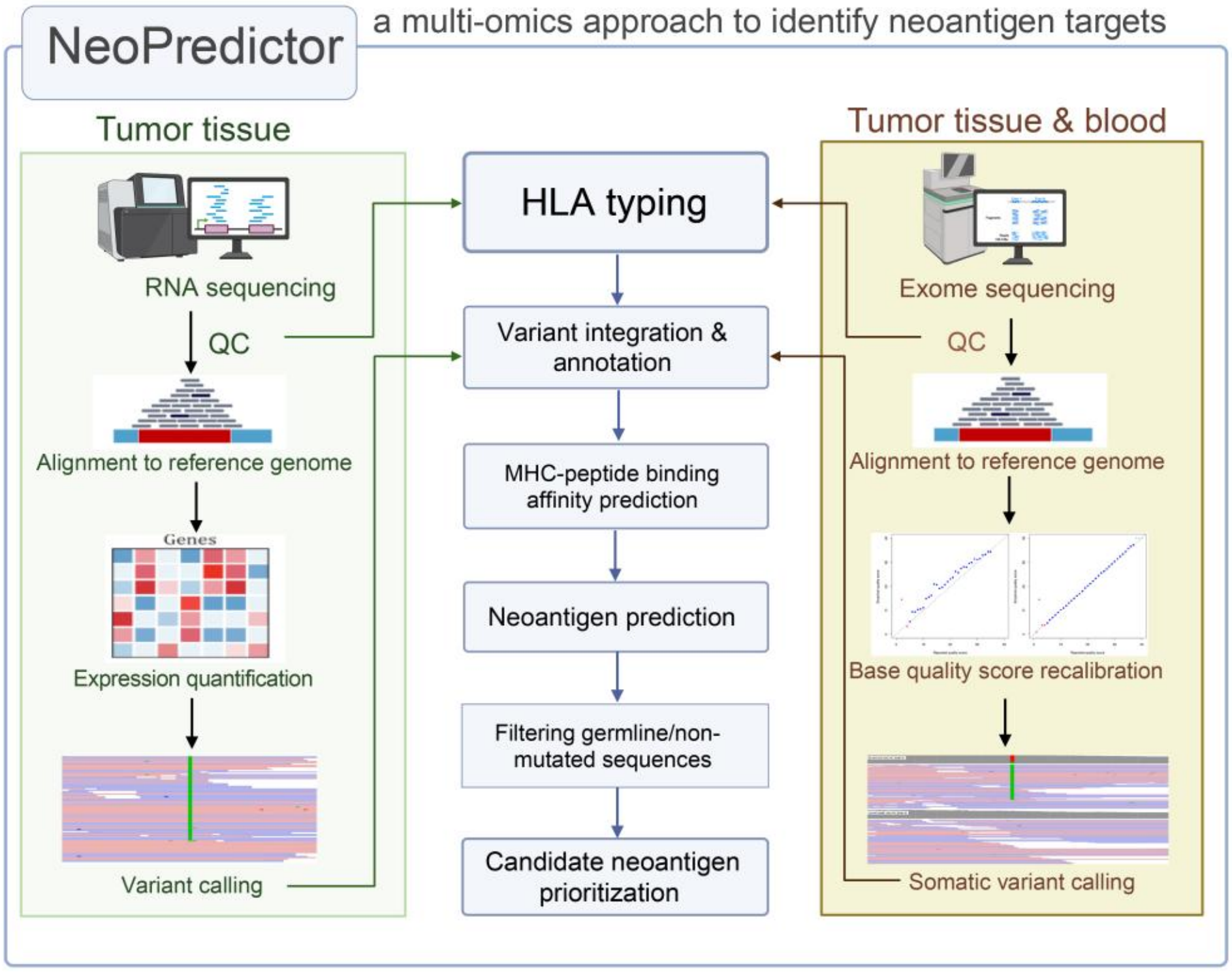

Figure S1. Workflow for neoantigen prediction. The figures are created in BioRender.com. QC, quality control.

Figure S2

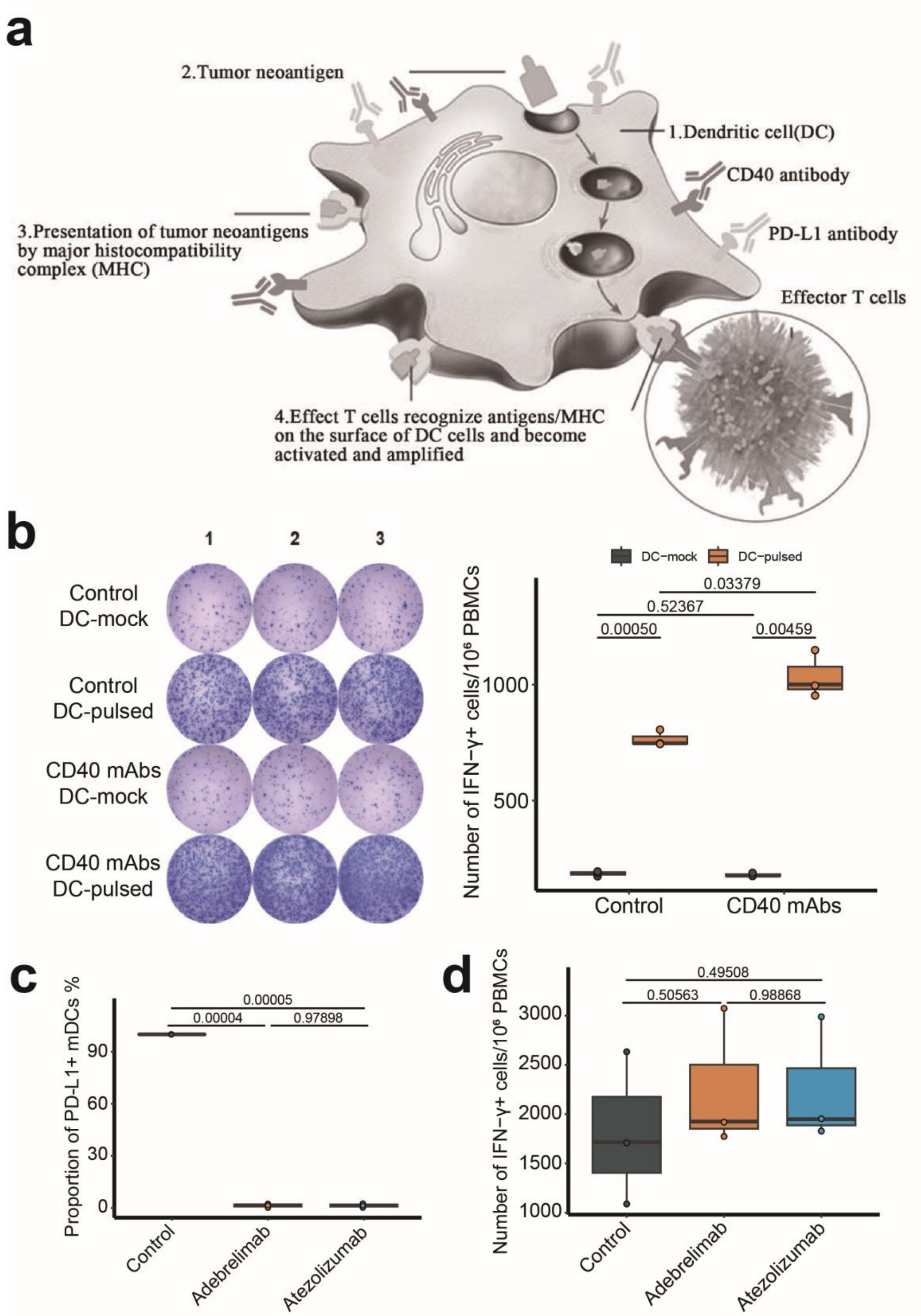

Figure S2. ZSNeo-DC enhanced with CD40 agonism and PD-L1 blockade. a Schematic diagram depicts the design of ZSNeo-DC vaccine, showing the vaccine is enhanced with CD40 agonism and PD-L1 blockade. **b** Representative images (left

panel) and a statistical plot (right panel) of the IFN-γ secreting ELISPOT assay performed 10 days after T-cell expansion, demonstrating that DCs treated with a CD40 agonist antibody (Cifurtilimab) enhance the neoantigen-specific immune response: a 35.5% increase relative to the untreated control, measured as counts of spot-forming cells (SFCs) per million input PBMCs. Two-sided t-tests were used for significance testing (n=3 technical triplicates per condition). The median and interquartile range with whiskers extending to the minimum and maximum values are depicted. No adjustment for multiple comparisons was applied. **c** DCs modified with two PD-L1 blockade antibodies (Adebrelimab and Atezolizumab) exhibiting a > 95% decrease of PD-L1 expression on the DC surface measured by Flow Cytometry. Two-sided t-tests were used for significance testing (n=3 technical triplicates per condition). The median and interquartile range with whiskers extending to the minimum and maximum values are depicted. No adjustment for multiple comparisons was applied. **d** DCs modified with two PD-L1 blockade antibodies (Adebrelimab and Atezolizumab) showing no significant impact on neoantigen-specific immune response, measured as counts of spot-forming cells (SFCs) per million input PBMCs with the IFN-γ release enzyme-linked immunospot assay. Two-sided t-tests were used for significance testing (n=3 technical triplicates per condition). The median and interquartile range with whiskers extending to the minimum and maximum values are depicted. No adjustment for multiple comparisons was applied.

Figure S3

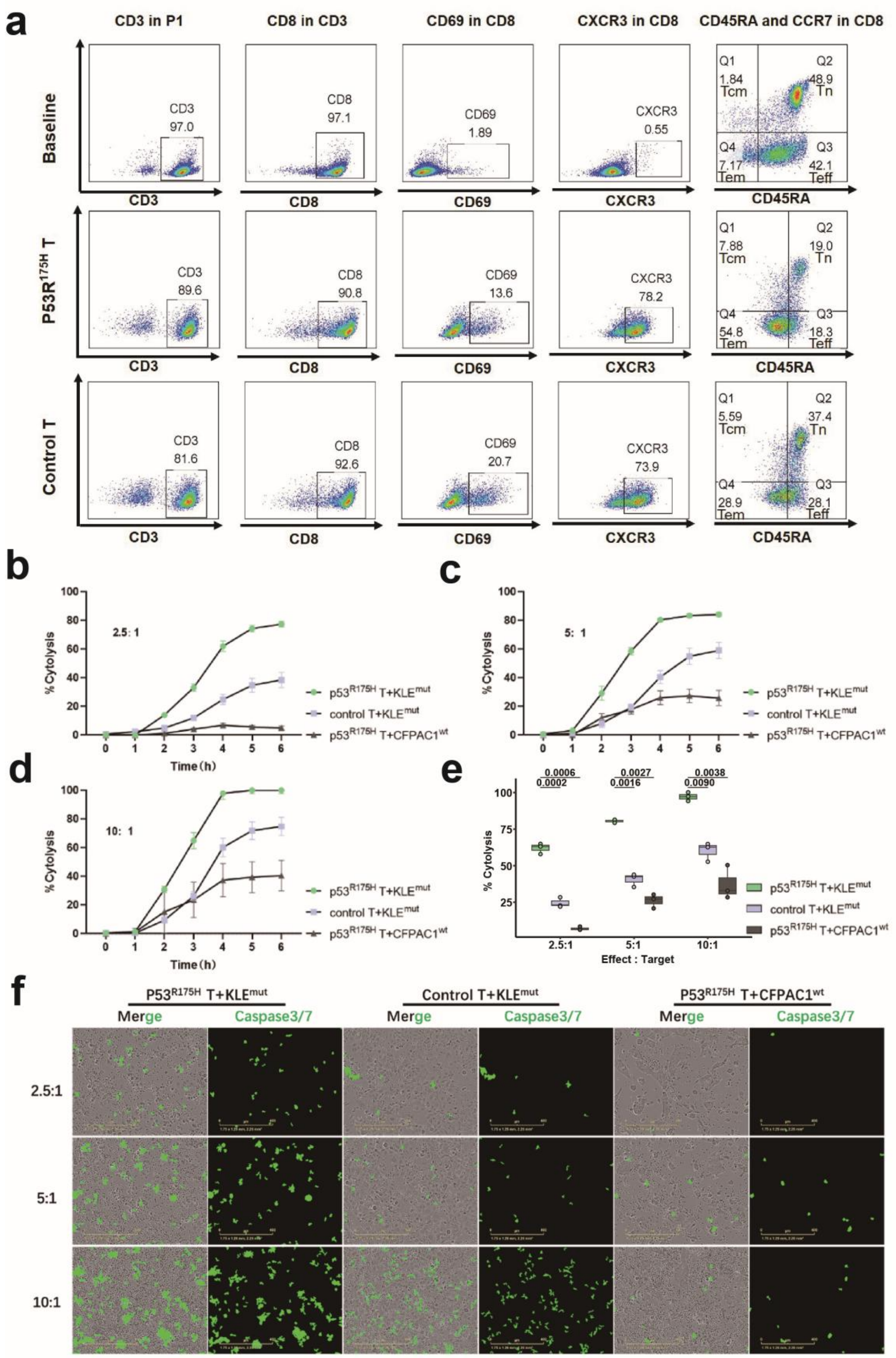
a
CD3 in P1
CD8 in CD3
CD69 in CD8
CXCR3 in CD8
CD45RA and CCR7 in CD8
Baseline
P53R175H T
Control T
CD3
CD8
CD69
CXCR3
CD45RA
b
c
d
e
f
%Cytolysis
Time (h)
2.5: 1
5: 1
10: 1
p53R175H T+KLEmut
control T+KLEmut
p53R175H T+CFPAC1wt
% Cytolysis
Effect : Target
P53R175H T+KLEmut
Control T+KLEmut
P53R175H T+CFPAC1wt
Merge
Caspase3/7
2.5:1
5:1
10:1

Figure S3. ZSNeo-DC promotes neoantigen-specific T cell activation, differentiation and tumor-killing. a Representative Flow cytometry images revealing a marked increase of activation (CD69 and CXCR3) in neoantigen-specific T cells ($P53^{R175H}$ T cells) compared to baseline, and a distinct pattern of T cell differentiation between $P53^{R175H}$ T cells and controls. $P53^{R175H}$ T cells and control T cells were generated by co-culture with ZSNeo-DCs pulsed with mutant or blank peptides, respectively. A synthetic 21-aa neoantigen peptide (QSQHMTEVVRHCPHHERCSDS) was used. T cells were expanded in CTL media at a 5:1 (T:DC) ratio, supplemented with IL-21 (30 ng/mL) initially, followed by IL-7/IL-15 (5-10 ng/mL) on days 3–7. **b, c, d** Cytolytic activity of CTLs against tumor cells was quantified using real-time impedance-based assays. Tumor cell lines (KLE^{mut} [$P53^{R175H}$ positive] or $CFPAC\text{-}1^{wt}$ [$P53^{R175H}$ negative]) were seeded into 96-well microplates pre-coated with gold microelectrodes for impedance monitoring. After 24 hours of adherence, effector cells ($P53^{R175H}$ T cells or control T cells) were added at specified effector-to-target (E:T) ratios: **(b)** 2.5:1, **(c)** 5:1, and **(d)** 10:1 (n=3 technical triplicates per condition). Cell Index (CI) was continuously monitored for 6 hours using the xCELLigence RTCA system. Data are shown as median values with 95% confidence intervals. **e** Summary of cytolytic activity at the 4-hour timepoint across different E:T ratios. $P53^{R175H}$ T cells exhibited significantly higher cytolytic activity against KLE^{mut} cells compared to control groups. Comparisons were analyzed using two-sided t-test for independent samples (n=3 technical triplicates per condition). The median and interquartile range with whiskers extending to the minimum and maximum values are depicted. No adjustment for multiple comparisons was applied. **f** Morphological observation of tumor cells co-incubation with CTLs at varying E:T ratios was performed using real-time fluorescence imaging. KLE^{mut} or $CFPAC\text{-}1^{wt}$ were seeded into culture plates and allowed to adhere for 24 hours. CTLs were then added at specified E:T ratios, alongside the apoptosis indicator Incucyte® Caspase-3/7 Green. After 12 hours of co-culture, fluorescence microscopy imaging was conducted using the Incucyte® Live-Cell Analysis System. Green fluorescent signals localized within tumor cells indicated caspase-3/7 activation, serving as a morphological hallmark of apoptosis.

Figure S4

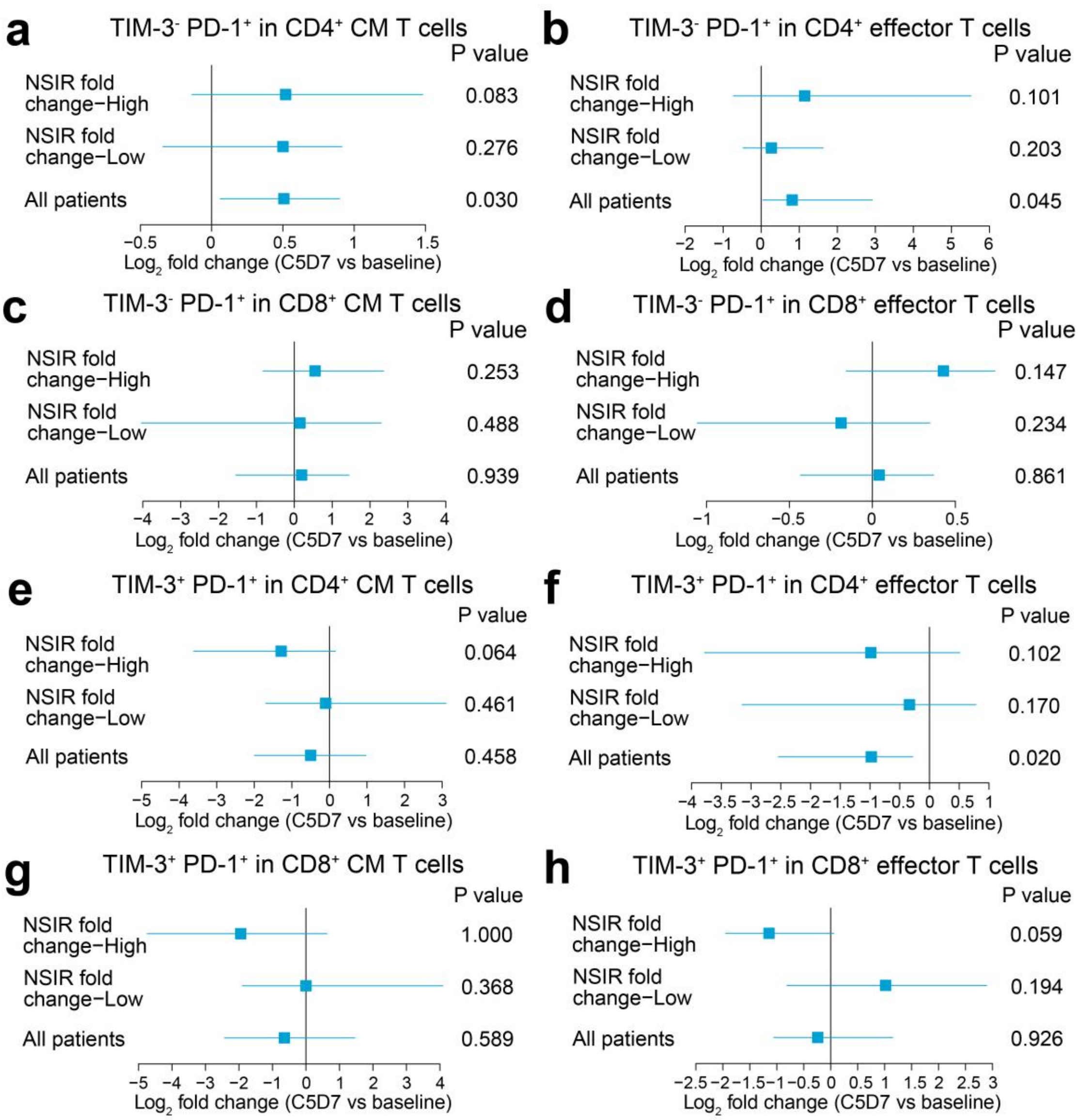

Figure S4. Alterations of peripheral T-cell activation and exhaustion phenotypes after ZSNeo-DC therapy. Forest plots showing the fold changes (C5D7 vs baseline) of proportions of activated (TIM-3$^-$ PD-1$^+$) and exhausted (TIM-3$^+$ PD-1$^+$) phenotypes in peripheral CD4/8$^+$ CM (central memory) effector T cells. Analyses were stratified by patients with the fold changes (C5D7 vs baseline) of neoantigen-specific immune response (NSIR) higher (n=5) and lower (n=5) than the median level, and all patients (n=10). Data is presented as the model estimate of the post-pre-change on a $\log_2(x + 1)$ scale with 95% confidence intervals (CI). The central point represents the median fold change. Paired two-sided t-tests were used for significance testing. No adjustment for multiple comparisons was applied.

Figure S5

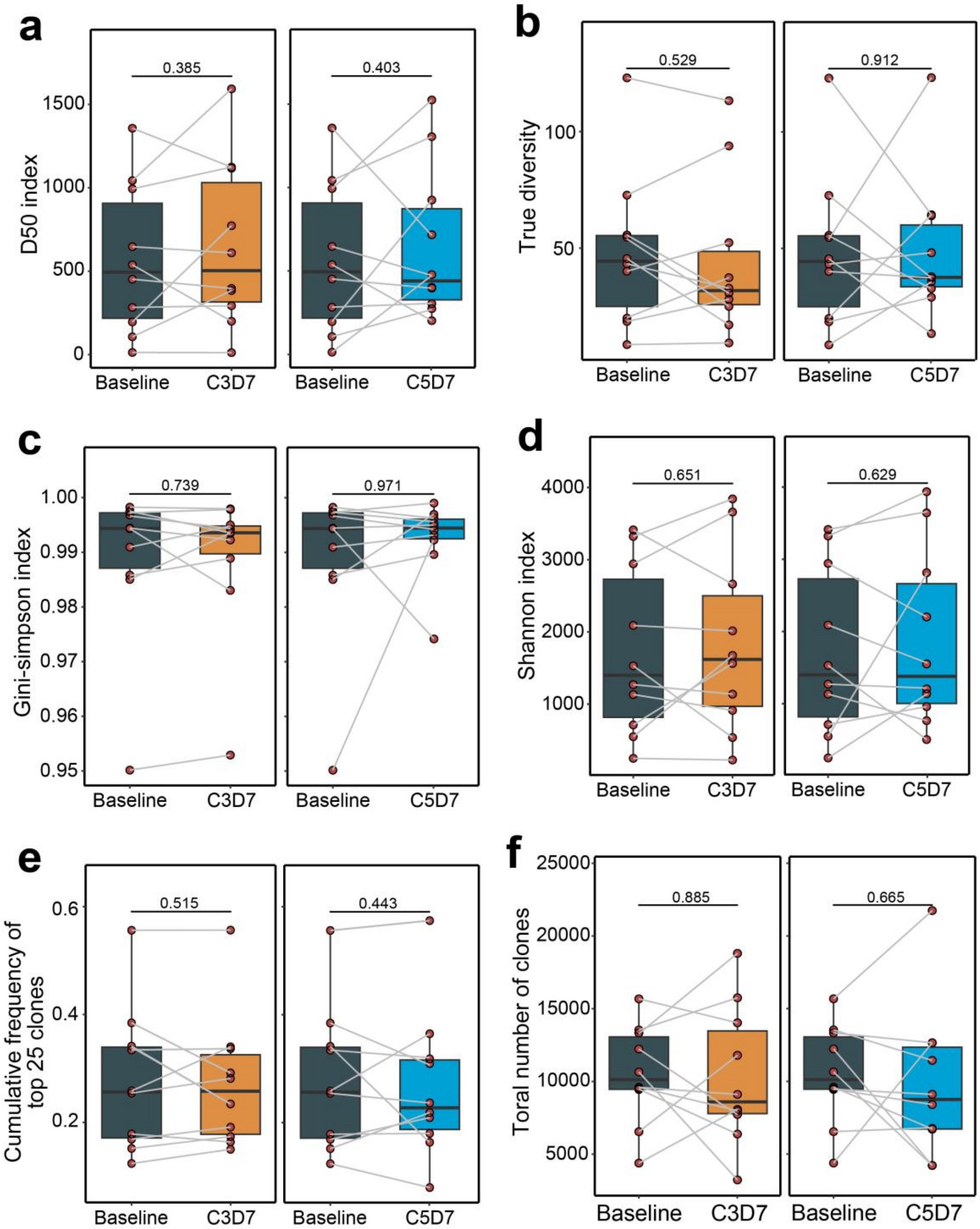

Figure S5. Alterations of peripheral TCR repertoire diversity after ZSNeo-DC therapy. a, b, c, d, e, f Indicators of T cell receptor (TCR) repertoire diversity, including **(a)** D50 index, **(b)** true diversity, **(c)** Gini-simpson index, **(d)** Shannon index, **(e)** Cumulative frequency of top 25 clones, and **(f)** Total number of clones, were compared among time-points. Two-sided t-tests or Wilcoxon rank-sum tests were applied to assess statistical significance, based on normality testing (n=10). The median and interquartile range with whiskers extending to the minimum and

maximum values are depicted. No adjustment for multiple comparisons was applied.

Figure S6

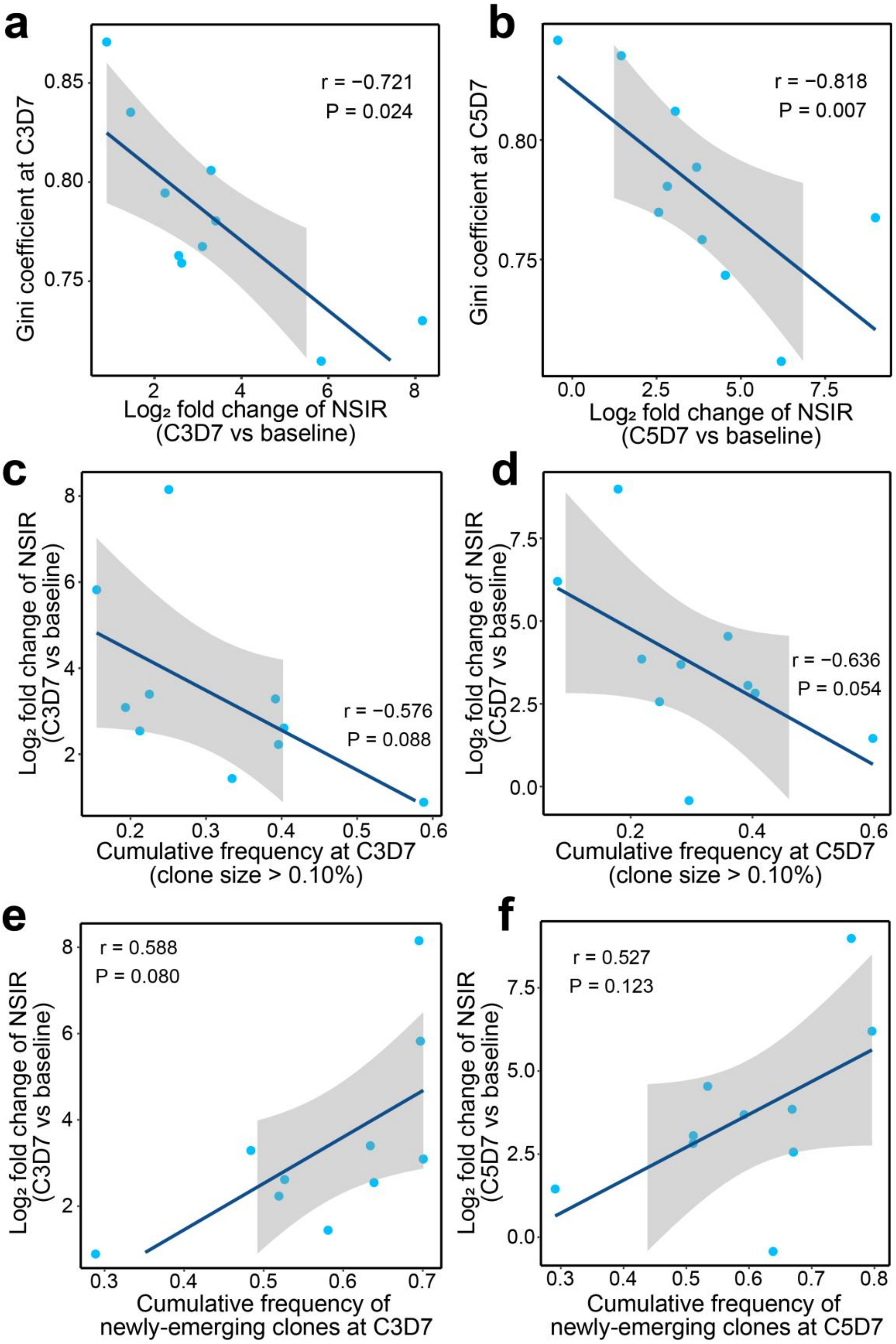

Figure S6. Correlations between TCR repertoire features and neoantigen-specific

immune response changes after ZSNeo-DC therapy. a, b Correlations between fold changes of neoantigen-specific immune response (NSIR) at **(a)** C3D7 and **(b)** C5D7 relative to baseline and the Gini coefficient score at the according timepoints (n=10). **c, d** Correlations between fold changes of NSIR at **(c)** C3D7 and **(d)** C5D7 relative to baseline and the cumulative frequencies of large-sized (frequency > 0.1%) TCR clones at the according timepoints (n=10). **e, f** Correlations between fold changes of NSIR at **(e)** C3D7 and **(f)** C5D7 relative to baseline and the cumulative frequencies of newly emerging TCR clones at the according timepoints (n=10). Fold changes are shown on a $\log_2(x+1)$ scale. Associations were assessed using two-sided Spearman's rank correlation. The solid line represents the fitted linear regression line for visualization, and the grey shaded band indicates the 95% confidence interval of the fitted line. No adjustment for multiple comparisons was applied.

Supplemenary Note 2

Safety & Efficacy of DC Vaccine and TMZ for the Treatment of Newly-diagnosed Glioblastoma After Surgery

Study Protocol

Version: 3.0

Version Date: 2022-06-30

Confidentiality Statement

This document contains confidential information of the Sponsor. Any institution or individual receiving this document shall not publish or disclose any unpublished information provided herein without prior written approval.

Protocol Amendment

After approval by the Ethics Committee (EC), any modifications to this protocol require the preparation of a protocol amendment explanation, signed by the Principal Investigator, and approved by the Sponsor. After amendment, the protocol must be reviewed or filed by the EC before implementation.

**Protocol/Amendment Number**	**Version Number**	**Date Prepared**	**Amendment Content**
	1.0	2020-09-22	Not Applicable
	2.0	2021-03-11	Adjustment of dosing frequency, revision of inclusion criteria, correction of text content, clarification of blood collection volume, supplementation of adverse
	2.1	2022-01-21	Modification of exclusion criteria description, modification of trial procedures
	3.0	2022-06-30	Modification of trial procedures and ancillary forms

Protocol Signature Page

Investigator Statement:

I agree to conduct the clinical research strictly in accordance with the design and specific provisions of this protocol, comply with all laws and regulations related to clinical research, and protect the safety and rights of the subjects. During the research process, I will strictly adhere to the current GCP and the Declaration of Helsinki, and commit that the entire research process will meet ethical, moral, and scientific requirements.

		Year Month Day
____________________	_______________	______________________
Name (Printed)	Signature	Date

Abstract

**Study Title**	Safety & Efficacy of DC Vaccine and TMZ for the Treatment of Newly-diagnosed Glioblastoma After Surgery
**Indication and Target Population**	Newly diagnosed glioblastoma patients
**Study Objectives**	**Primary Objective:**<br>To evaluate the safety of personalized neoantigen-pulsed autologous DC vaccine combined with standard TMZ treatment regimen in newly diagnosed glioblastoma patients.
	**Secondary Objectives:**<br>1）To evaluate the immunological efficacy of DC vaccine in newly diagnosed glioblastoma patients;<br>2）To evaluate the preliminary clinical efficacy of DC vaccine combined with standard TMZ treatment regimen in newly diagnosed glioblastoma patients;<br>3）To analyze the correlation between immune molecular characteristics of peripheral blood and cerebrospinal fluid, immune cell phenotypes, and anti-tumor immune responses induced by the DC vaccine;<br>4）To evaluate the role of immune molecular characteristics of peripheral blood and cerebrospinal fluid, and immune cell phenotypes in predicting the efficacy of DC vaccine therapy.
**Trial Design**	This study is a single-center, single-arm, prospective Phase I clinical trial enrolling postoperative patients with newly diagnosed glioblastoma. Participants will receive DC vaccination in combination with standard adjuvant TMZ chemotherapy. A total of ten subjects are planned for enrollment. The investigational product consists of non-genetically engineered autologous DCs pulsed with multiple tumor neoantigen peptides, administered via five multi-site injections into the inguinal or axillary lymph node regions and adjacent subcutaneous tissue. Depending on individual tolerance and compliance, investigators may extend the regimen up to a maximum of eight injections per course. In

	patients demonstrating favorable tolerability, immunologic activity, and clinical benefit, peripheral blood samples will be collected following completion of adjuvant TMZ for immune monitoring, and an additional 1–2 booster courses of DC vaccination (5–8 injections per course) may be administered.<br>Throughout treatment, immune responses and adverse events will be closely monitored by molecular, cellular, and clinical assessments, with timely interventions as required. Safety evaluation will include systematic monitoring of adverse events, physical examinations, vital signs, electrocardiograms (ECG), hematology, cytokine assays, and other relevant laboratory tests. Efficacy evaluation will focus on pre- and post-treatment comparisons of cytokine concentrations and profiles, immune cell frequencies and distributions, phenotypic and molecular characteristics of treatment-induced T-cell subsets, and additional molecular and clinical biomarkers.
**Inclusion Criteria**	Subjects must meet all of the following criteria to be eligible:<br>1) Male or female aged 18-75 years (inclusive);<br>2) Newly diagnosed glioblastoma patients confirmed by histopathology (WHO Grade 4);<br>3) Molecular pathological diagnosis confirming wild-type IDH1 and IDH2 genes;<br>4) Patients with ≥90% resection of the contrast-enhancing lesion;<br>5) KPS score ≥ 60 before DC vaccine therapy;<br>6) Normal bone marrow reserve, normal liver and kidney function, and normal cardiac function before DC vaccine therapy:<br>• Absolute white blood cell count ≥ 2.5×10^9/L;<br>• Hemoglobin > 100 g/L;<br>• Platelet count > 100×10^9/L;<br>• ALT, AST < 2.5 x ULN;<br>• Serum creatinine < 1.5 x ULN.

**Exclusion Criteria**	1) Presence of other uncontrolled malignancies;<br>2) Carmustine (BCNU) sustained-release wafer implantation within 6 months;<br>3) Active hepatitis B or C, HIV infection;<br>4) Grade 2-3 hypertension with poor blood pressure control;<br>5) Patients with severe cardiovascular or cerebrovascular diseases such as coronary heart disease, angina, myocardial infarction, arrhythmia, cerebral thrombosis, cerebral hemorrhage, etc.;<br>6) Any other uncontrolled active diseases that hinder participation in the trial, such as autoimmune disorders (e.g., hemolytic anemia, psoriasis, rheumatoid arthritis);<br>7) History of poorly controlled psychiatric disorders, or subjects with medical or mental health history that, in the investigator's opinion, may increase the risk associated with study participation or study drug administration, or may interfere with results;<br>8) Long-term use of immunosuppressants due to reasons such as organ transplantation;<br>9) Systemic use of high-dose steroids (>10mg/d prednisone or equivalent dose of other steroids) within 4 weeks before autologous DC vaccine application, excluding recent or current inhaled corticosteroid therapy;<br>10) Unstable pulmonary embolism, deep vein thrombosis, or other major arterial/venous thromboembolic events within 30 days prior to enrollment, or current anticoagulant therapy;<br>11) Pregnant or breastfeeding subjects, or subjects planning pregnancy during treatment or within 2 months after treatment;<br>12) Active or uncontrolled infection requiring systemic treatment within 14 days before enrollment (excluding simple urinary tract infections or upper respiratory tract infections);<br>13) Patients who received other vaccine therapies or other genetically modified cell therapies prior to enrollment;<br>14) After tissue sample sequencing and analysis, predicted neoantigen peptides are fewer than 5;<br>15) Subjects deemed unsuitable for trial participation by the investigator;<br>16) Subjects with conditions that impair their ability to provide written informed consent or comply with study

	procedures, or who are unwilling or unable to adhere to study requirements;<br>17) Subjects who have participated in other drug clinical trials within 4 weeks before enrollment or are currently participating in other drug clinical trials.
**Drug Dosage/Routes of Administration/Regimen**	Each dose of the DC vaccine contains (2-10)×10^6 DC cells loaded with 5-20 tumor neoantigen peptides in a volume of 1 mL, administered as multiple intradermal injections in the perinodal skin of the inguinal or axillary lymph node regions. Doses are scheduled at weeks 3, 4, 5, 8, and 12 after concurrent chemoradiotherapy (five injections constitute one course; see study flowchart). After the initial five injections, the investigator may increase the number of injections based on the subject's tolerance and adherence; in principle, the total number of injections per course should not exceed eight.<br>For subjects demonstrating favorable treatment tolerability, immunological responses, and clinical responses, peripheral blood will be collected after completion of TMZ adjuvant chemotherapy to assess immune response. Based on the assessment, an additional 1-2 courses of DC vaccination (5-8 injections per course) may be administered as booster therapy.
**Safety Assessment**	This project uses NCI CTCAE 5.0 to grade adverse events and serious adverse events. Investigators must monitor the safety of all subjects who are enrolled and receive at least one dose of the DC vaccine, and record any AEs and SAEs. Toxicities, immune responses, and long-term malignant events should be observed. Investigators must provide appropriate medical care throughout the study and follow up any SAEs or AEs leading to treatment discontinuation via appropriate healthcare measures. Follow-up should continue until the AE/SAE resolves or a reasonable explanation is established. The frequency of follow-up may be determined at the investigator's discretion.
**Efficacy Assessment**	**Immunological Efficacy Assessment:**<br>Before DC vaccine injection (at the time of apheresis), and one week after the 3rd, 5th, and the last injection of each course (if more than 5 injections), use ex vivo enzyme-linked immunospot (ELISPOT) assay to detect the number of subjects with specific IFN-γ+ T cells in peripheral blood PBMC after treatment with their own tumor neoantigens

	(positive defined as at least 55 spot-forming cells (SFC) per 10^6 PBMC or a ≥ 3-fold increase from baseline).<br>**Clinical Efficacy Assessment:**<br>After completing personalized DC vaccine therapy for postoperative newly diagnosed glioblastoma patients, treatment efficacy will be assessed using iRANO criteria. Record progression-free survival (PFS, defined as the time from surgery to tumor recurrence or progression), overall survival (OS, defined as the time from surgery to patient death), 1-year survival rate (defined as the proportion of all DC-treated patients with OS exceeding 1 year), and quality of life assessment (QOL). If measurable lesions are present, evaluate short-term efficacy (CR, PR, PD, SD), disease control rate (DCR), and clinical benefit rate (CBR). Observe changes in the immune microenvironment during and after treatment and analyze their correlation with the clinical efficacy of the vaccine.
**Study Duration**	Expected Study Start Date: May 2021<br>Expected Study End Date: December 2023
**Efficacy Analysis**	Descriptive statistics will be used for statistical description of efficacy indicators. Kaplan-Meier curves will be used to describe PFS and OS, calculating median PFS and median OS. Calculate RR, DCR, CBR, and 95% confidence intervals.

Table of Contents

Protocol Amendment 2

Protocol Signature Page 3

Abstract 4

I. Background Introduction 13

1.1 Current Status of Clinical Diagnosis and Treatment of Malignant Glioma 13

1.2 Immunotherapy for GBM 15

1.2.1 Conventional Immunotherapy 15

1.2.2 Therapeutic Cancer Vaccines 16

1.3 Personalized DC Vaccine for GBM Treatment 19

1.4 Preliminary Work 21

II. Study Objectives and Rationale 23

2.1 Primary Objective 23

2.2 Secondary Objectives 23

2.3 Significance of the Project 24

III. Project Design 25

3.1 Project Overview 25

3.1.1 Overview 25

3.1.2 Reference Protocols 25

3.2 Project Implementation Plan 26

3.2.1 Implementation Plan Overview 26

3.2.2 SOP for Collection of Tumor and Peritumoral Tissue 29

3.2.3 Blood Sample Collection and Processing SOP 30

3.2.4 SOP for Prediction of Personalized Tumor Neoantigens and Peptide Synthesis 30

3.2.5 SOP for Preparation and Quality Control of Neoantigen-Pulsed DC 31

3.2.6 Pre-treatment Regimen 31

3.2.7 Pre-treatment Baseline Assessment 31

3.2.8 Dosage and Administration 31

3.2.9 Immune Monitoring Plan 32

3.2.10 24 Hours After Vaccine Injection 33

3.2.11 Infection and Other Toxic Reactions 33

IV. Case Selection 35

4.1 Screening Records 35
4.2 Inclusion Criteria 35
4.3 Exclusion Criteria 35
4.4 Subject Completion/Non-Completion of the Study 36
4.4.1 Completion 36
4.4.2 Treatment Discontinuation 37
4.4.3 Study Withdrawal 37
V. Study Evaluation 38
5.1 Primary Endpoints (Safety Assessment) 38
5.2 Secondary Endpoints (Efficacy Assessment) 41
5.3 Clinical Monitoring Parameters and Assessment Schedule 41
5.3.1 Vital Signs Monitoring 41
5.3.2 Toxicity Assessment Indicators 41
5.3.3 Immune Efficacy Indicators 42
5.3.4 Other Immune Response Monitoring Indicators 42
5.3.5 Clinical Efficacy Evaluation 42
5.4 Quality of Life Assessment 43
VI. Adverse Reactions and Treatment Recommendations 43
6.1 Possible Adverse Reactions (Adverse Reaction Record Form) 43
6.2 Diagnosis and Treatment Recommendations for Cytokine Release Syndrome (CRS) 46
VII. Adverse Event Reporting 48
7.1 Definition 48
7.1.1 Definition and Classification of Adverse Events 48
7.1.2 Definition of Causality 49
7.2 Operational Procedures 50
7.2.1 All Adverse Events 50
7.2.2 Serious Adverse Event 51
7.2.3 Pregnancy 52
7.3 Contacting the Sponsor Regarding Safety Issues 52
8. Statistical Methods 52
8.1 Patient Enrollment and Analysis Population Description 52
8.2 General Principles of Statistical Analysis 53
8.3 Statistical Description and Analysis of Baseline Data 53

8.4 Missing Values and Outliers 53
8.5 Safety Analysis 53
8.6 Efficacy Analysis 54
9. Ethical Principles 54
9.1 Ethical Considerations in Study Design 54
9.2 Ethical Compliance 55
9.2.1 Investigator Responsibilities 55
9.2.2 Independent Ethics Committee/Institutional Review Board (IEC/IRB) 55
9.2.3 Informed Consent 56
9.2.4 Privacy of Personal Data 57
10. Document Management 58
10.1 Study Protocol Amendments 58
10.2 Registration Documents 59
10.2.1 Registration Approval/Notification 59
10.2.2 Pre-study Required Documents 59
10.3 Subject Identification Code List and Subject Screening/Enrollment Log 60
10.4 Source Documents 60
10.5 Completion of Case Report Forms 61
10.6 Data Quality Assurance/Quality Control 61
10.7 Record Retention 62
10.8 Study Completion/Termination 63
10.8.1 Study Completion 63
10.8.2 Study Termination 63
10.9 Site Audit 63
10.10 Clinical Trial Registration and Results Disclosure 63
11. Anticipated Progress 64
12. References 65
13. Appendices 67
Appendix 1 Karnofsky Performance Status (KPS) Scale 67
Appendix 2 Eastern Cooperative Oncology Group (ECOG) Performance Status Scale (ZPS, 5-point scale) 68
Appendix 3 NCI-CTCAE V5.0 Grading Criteria for Common Acute and Subacute Toxicities 69
Appendix 4 NCI CTCAE 5.0 Adverse Event Grading 71

Appendix 5 FACT-G Quality of Life Score 74

Appendix 6 EORTC QLQ-C30 Quality of Life Questionnaire (Version 3.0) 77

Appendix 7 Study Flow Chart 80

I. Background Introduction

1.1 Current Status of Clinical Diagnosis and Treatment of Malignant Glioma

Glioma is a tumor arising from neuroectodermal tissue. Its common classifications include astrocytoma, oligodendroglioma, ependymoma, etc. Among these, astrocytoma is the most common glioma, accounting for 40% of gliomas, and can grow anywhere in the brain or spinal cord [1]. In adults, astrocytomas mostly occur in the cerebrum, while in children they are often found in the cerebellum and brainstem. The World Health Organization (WHO) classifies central nervous system tumors into four grades, with glioblastoma multiforme (GBM) being the most malignant, invasive, and having the poorest prognosis as WHO grade 4 [2]. GBM accounts for approximately 54.7% of all gliomas. Primary GBM originates within the brain parenchyma, while secondary GBM results from further malignant transformation of low-grade gliomas (grades 2 and 3) [3]. Pathologically, GBM is characterized by high cellularity within the tumor tissue, large tumor cells, significant nuclear atypia, frequent mitoses, vascular endothelial cell proliferation, the presence of numerous immature blood vessels, and may be associated with extensive hemorrhage and necrosis [4]. Primary GBM mostly occurs in middle-aged and elderly patients over 55 years old, while secondary GBM is more common in patients under 55 years old. The average time for WHO grade 4 and WHO grade 3 gliomas to develop into GBM is 5 years and 2 years, respectively [1, 5], with a median survival time of approximately 1 year [8].

Over the past 30 years, the incidence of primary malignant brain glioma has been increasing annually, with an annual growth rate of about 1.2%. According to statistics from the Central Brain Tumor Registry of the United States (CBTRUS), the global incidence of primary central nervous system tumors is approximately 21.42 per 100,000 people, with about 34% being malignant. Among all primary malignant brain tumors, glioma has the highest incidence rate [1]. Data released by the China National Cancer Registry in 2014 show that gliomas account for 32% of all primary central nervous system tumors in China, about 45% of all intracranial tumors, and 81% of malignant central nervous system tumors. The incidence of malignant glioma is (5-8) per 1 million people, with a 5-year mortality rate

ranking third, second only to pancreatic cancer and lung cancer. Among primary malignant central nervous system tumors, GBM has the highest incidence, accounting for 45.6%, approximately 3.19 cases per 100,000 people, and is more common in males than females. The incidence of GBM increases with age, with the highest incidence in the 75-84 age group. The median age at diagnosis of newly diagnosed GBM is 64 years [6].

Because GBM exhibits expansive infiltrative growth with no clear boundary from normal tissue, surgery alone cannot achieve a cure, and there is a high risk of postoperative recurrence and poor prognosis [7]. Clinically, effective treatment methods are currently lacking, and the main approach remains surgical tumor resection combined with comprehensive treatments such as radiotherapy and chemotherapy [9]. Surgery can alleviate clinical symptoms, and radiotherapy can kill or inhibit residual tumor cells, prolonging patient survival. Because GBM is characterized by local recurrence, with 90% occurring within 2 cm of the primary lesion, optimizing local radiotherapy regimens is a focus of treatment. However, due to radiation dose limitations and radioresistance, clinical outcomes are poor. In recent years, various dose fractionation methods, different radiotherapy techniques (such as three-dimensional conformal radiotherapy, intensity-modulated radiotherapy, interstitial brachytherapy, stereotactic radiosurgery, etc.), and the application of new radiotherapy equipment have somewhat improved radiotherapy efficacy [10]. Chemotherapy employs both single-agent and multi-drug combination regimens. The advent of temozolomide is considered the most significant advancement in glioma chemotherapy in nearly 40 years. Large-scale phase III clinical trials by the European Organisation for Research and Treatment of Cancer (EORTC) and the National Cancer Institute of Canada (NCIC) confirmed that temozolomide (TMZ) combined with concurrent radiotherapy, followed by 6 cycles of TMZ adjuvant chemotherapy, can extend patient survival [9, 11]. TMZ concurrent radiotherapy combined with adjuvant chemotherapy has become the standard treatment for newly diagnosed GBM. Currently, TMZ and carmustine biodegradable polymers (BCNU, Gliadel Wafer) have been approved in Europe and the United States for the treatment of malignant glioma [12]. Novel therapies such as molecular targeted therapy, immune checkpoint inhibitor-based immunotherapy, and gene therapy are gradually being applied to GBM treatment, but their efficacy still requires validation in large,

randomized, controlled studies. Overall, currently available clinical treatment options are still not ideal for GBM, which has high recurrence rates and chemoresistance. Even at great cost, the 5-year overall survival (OS) for GBM patients remains less than 10% [11], urgently necessitating the search for new treatment methods to substantially improve the prognosis of GBM patients.

1.2 Immunotherapy for GBM

1.2.1 Conventional Immunotherapy

It has now been proven that malignant brain tumors are not immune deserts [13]. The discovery of lymphatic vessels within the CNS has shown that malignant brain tumors can be infiltrated by lymphocytes originating from the periphery [14], and the peripheral immune system can mount a therapeutically significant immune attack against existing malignant brain tumors [15]. Based on this understanding, various types of tumor immunotherapy have been used in clinical research for malignant gliomas, especially GBM [37].

Cancer immunotherapy includes adoptive immunotherapy (Adoptive Cell Transfer, ACT) such as CAR-T (Chimeric antigen receptor T cell), active immunotherapy such as therapeutic cancer vaccines, and passive immunotherapy such as CTLA-4, PD-1, and PD-L1. Unlike traditional anti-tumor drugs, cancer immunotherapy does not directly target tumor cells but rather the body's own immune system [16, 17]. It activates or modulates the immune system to promote immune cells such as tumor-infiltrating lymphocytes to recognize and eliminate tumor cells, generating a dynamic and sustained immune response to achieve therapeutic effects. Currently, the FDA has approved six immune checkpoint inhibitors including CTLA-4, PD-1, and PD-L1, as well as two CAR-T drugs for market use.

Adoptive cell immunotherapy is a treatment method that involves activating and expanding tumor-specific immune cells (primarily T cells) in vitro before reinfusing them into the patient. It has gone through stages such as LAK, TIL, and CIK, but due to poor targeting, it has performed poorly in clinical trials. The subsequent emergence of CAR-T technology addressed the targeting issue of T cells, representing a major advancement in adoptive immunotherapy [18]. In a CAR-T Phase I clinical trial (NCT02209376) conducted by the University of Pennsylvania and the Novartis Institutes for Biomedical Research,

which targeted EGFRvIII as a tumor antigen for glioblastoma (GBM) treatment [19], 10 patients with recurrent GBM received CAR-T therapy targeting EGFRvIII. The safety profile was acceptable for all patients, and the genetically modified T cells successfully migrated and infiltrated the GBM tumor tissue. However, due to the strong immunosuppressive tumor microenvironment of GBM, immunosuppressive molecules such as IDO, PD-L1, FoxP3, IL-10, TDO, and TGF-beta were upregulated in the GBM tumors after CAR-T cell infusion. Together with the neurotoxicity and off-target effects of CAR-T itself, these factors ultimately led to the failure of this clinical study. Passive immunotherapy, represented by anti-PD-1 and PD-L1 monoclonal antibodies, targets immune-related pathways to inhibit specific immunosuppressive mechanisms, thereby enhancing anti-tumor immune responses [20]. High mutational burden is considered essential to benefit from checkpoint inhibitor therapy [21]. However, GBM has low immune cell infiltration and low tumor mutational burden, with an average of only 20 to 50 non-synonymous mutations per tumor, resulting in GBM patients not fully benefiting from immune checkpoint inhibitor therapy [22].

1.2.2 Therapeutic Cancer Vaccines

Active immunotherapy primarily refers to therapeutic cancer vaccines, which employ processed tumor cell extracts, in vitro-synthesized tumor antigens, or dendritic cells (DCs) loaded with tumor antigens to generate vaccine formulations. These are administered to patients to stimulate or enhance tumor-specific immune responses, thereby eliminating tumor cells and preventing tumor growth and dissemination. Based on the source of antigens and the delivery platform, therapeutic cancer vaccines can be categorized into tumor antigen peptide vaccines, DNA and RNA vaccines, DC vaccines, etc. [23]. In vivo, antigen-presenting cells must take up tumor antigens released by tumor cells, process them into peptides within the cells, and then present them on the cell surface as "peptide-MHC complexes" to be recognized by naive T cells. This process enables T cells to recognize, attack, and eliminate tumor cells carrying the tumor antigen. DCs are the most potent professional antigen-presenting cells (pAPCs) known in the body and the only APCs capable of activating resting T cells, playing a critical role in anti-tumor immune responses. DC vaccines involve isolating precursor DC cells from the patient, culturing them in vitro, loading them with

tumor antigens, and then reinfusing them into the patient to stimulate specific anti-tumor T cell responses through DCs. Results from hundreds of clinical studies on therapeutic cancer vaccines conducted globally indicate that DC vaccines have the best and most stable clinical efficacy among various types of vaccines, with autologous DC vaccines being highly safe [24, 37]. In 2010, the U.S. FDA approved sipuleucel-T—the first therapeutic cancer vaccine for prostate cancer—which is an autologous DC-based vaccine [25].

In terms of the antigens delivered, therapeutic cancer vaccines have undergone two generations of technological iterations, progressing from the era of Tumor-Associated Antigens (TAAs) starting in the 1990s to the current era of Tumor-Specific Antigens (TSAs). From 1990 to 2000, TAAs combined with various vaccine adjuvants were used to create cancer vaccines that entered clinical trials, such as peptide vaccines based on tumor antigens like MUC1, MAGE-A3, and HER2, numerous TAA-pulsed DC vaccines, and TAAs-based DNA and RNA vaccines based on viruses, bacteria, and virus-like particles. However, all these attempts failed, and the only approved vaccine, Provenge, ended poorly due to efficacy and cost issues. Around 2011, the second generation of personalized therapeutic cancer vaccine technology based on TSAs began to emerge. In July 2017, Nature published two articles from different teams [35, 36]. Catherine Wu's team conducted a Phase I clinical study using a TSA-based peptide vaccine, enrolling six high-risk malignant melanoma patients (Stage III or IV) post-surgery. After vaccination, during a 20-32 month follow-up, all four Stage III patients showed no recurrence, while the two Stage IV patients experienced recurrence but achieved complete remission after PD-1 monoclonal antibody treatment. Sahin's team used a TSA-based RNA vaccine, enrolling 13 Stage III-IV melanoma patients. After intranodal vaccination with RNA encoding multiple neoantigen epitopes, metastasis incidence significantly decreased. Eight patients who were tumor-free at enrollment remained recurrence-free during a 12-23 month follow-up, while among the five patients with metastases, two achieved remission, one had stable disease, and one achieved complete remission after combined PD-1 monoclonal antibody treatment. These studies successfully demonstrated the feasibility and promising clinical application prospects of developing neoantigen-targeted cancer vaccines. In April 2018, Science Translational Medicine reported a TSA-based DC vaccine achieving a 100% two-year overall survival rate in

advanced ovarian cancer treatment [26]. Twenty-five advanced ovarian cancer patients received vaccine injections every three weeks for up to six months. Eleven patients who mounted an immune response to the vaccine achieved a 100% two-year overall survival rate, compared to only 25% in non-responders. One 46-year-old Stage IV ovarian cancer patient remained progression-free for five years after vaccine treatment. At the 2018 ASCO annual meeting, results from cancer vaccine clinical trials were also exciting. One clinical trial for endometrial and mucinous cancer showed that patients who relapsed after PDS (primary debulking surgery) and chemotherapy achieved durable immune responses with autologous DCVAC (dendritic cell vaccine) presenting tumor antigens. Sequential use of DCVAC and chemotherapy significantly improved PFS [27]. Currently, ClinicalTrials.gov records 68 global TSA studies, and therapeutic cancer vaccines based on tumor-specific antigens are becoming the focus of next-generation personalized drug development.

The clinical outcomes of these two generations of therapeutic cancer vaccine technologies differ fundamentally due to their different targets (antigens). TAAs are recognized by the immune system as self-components, resulting in weak T cell recognition and response. In contrast, TSAs are abnormal protein fragments caused by gene mutations in tumor cells that can activate the immune system. They are unique to tumor cells, highly specific, and capable of inducing long-term memory. The antigen recognition component TCR on T cells has high affinity for TSAs. However, the clinical application of TSA technology also faces challenges. Although TSAs are highly specific and immunogenic, they generate weak immune signals and are generally insufficient to induce immune rejection of tumors in vivo. Additionally, TSAs exhibit inter-individual heterogeneity, meaning different patients have different TSAs, making their discovery and identification difficult. This process requires a multi-platform approach involving genomic sequencing, transcriptome sequencing, bioinformatic analysis, proteomics, and functional immune validation, making it a highly complex and technically demanding systems endeavor/engineering that is not easy to master. Currently, research in this field is primarily led by Europe and the United States, while domestically it remains relatively undeveloped, requiring breakthroughs in technology and clinical research practice.

1.3 Personalized DC Vaccine for GBM Treatment

From an immunologic perspective, GBM is an immunologically 'cold' tumor with a relatively low burden of mutations and few neoantigens, with limited pre-existing antitumor effector cells, making it difficult to benefit from passive immunotherapy represented by immune checkpoint inhibitors. However, several clinical trials have preliminarily confirmed the efficacy of TSA-based therapeutic DC vaccines for GBM treatment. Currently, on the U.S. ClinicalTrials.gov website, 13 completed Phase I and II trials and 14 ongoing Phase I-III trials of DC vaccines for GBM have been registered. All show that therapeutic DC vaccines have mild or few adverse effects, and patients experience varying degrees of extension in progression-free survival (PFS) and overall survival (OS).

Scientists at the Dana-Farber Cancer Institute reported in Nature the results of a Phase 1b trial of a personalized neoantigen-targeted vaccine in newly diagnosed, MGMT promoter-unmethylated GBM [30]—the first demonstration of success for a neoantigen-based vaccine in an immunologically “cold” tumor. The researchers demonstrated that a personalized 'neoantigen' vaccine can activate an immune response against GBM, producing immune T-cells that migrate to the brain tumor, creating a 'hotter' inflamed environment around the cancer cells. Analysis of tumor-infiltrating T cell TCRs confirmed that the T cells infiltrating the tumor were neoantigen-specific T cells induced by the cancer vaccine, and that peripherally induced T cells could cross the blood-brain barrier to reach intracranial lesions. This is the first direct evidence that a therapeutic cancer vaccine can generate immune cells against GBM and transport them from the blood into the glioblastoma. Additionally, this study further confirmed that DC vaccines are very safe with no significant side effects. Keiichi Sakai et al. from Shinshu University School of Medicine in Japan conducted a Phase I trial to evaluate the safety and efficacy of a WT1-sensitized dendritic cell vaccine for GBM patients who relapsed after standard treatment. The results were published in the October 2015 issue of the Journal of Neurosurgery [28]. The study involved GBM patients who relapsed after standard treatment, were resistant to radiotherapy and chemotherapy, and had progressive disease. They received immunotherapy with WT1-sensitized or autologous tumor cell antigen-sensitized DC vaccines. Subcutaneous injections of 1×10^7 to 2×10^7 sensitized DC cells were administered every two weeks, with

5-7 injections constituting one course. Five patients treated with the WT1 peptide-sensitized DC vaccine achieved stable disease, and two of these five patients showed improved neurological function. MRI follow-up revealed tumor shrinkage. Aside from Grade 1-2 skin rash at the injection site, there were no other serious adverse reactions. The results indicate that WT1 peptide-sensitized DC vaccine therapy for recurrent malignant glioma is safe and feasible.

Scientists from Heidelberg University Hospital in Germany treated newly diagnosed GBM patients with a personalized DC vaccine based on tumor neoantigens [29]. After surgical resection of the tumor and standard radiotherapy and chemotherapy, patients were sequentially injected with APVAC1 and/or APVAC2 vaccines, along with granulocyte-macrophage colony-stimulating factor (GM-CSF) and poly-ICLC as adjuvants. Among patients injected with APVAC1, 92.3% developed a CD8+ T cell immune response to at least one antigenic peptide, with an increase in the number of corresponding specific CD8+ T cells in the blood and the emergence of a memory phenotype. Among patients injected with APVAC2, 80% showed a neoantigen-specific CD4+ T cell response, primarily a Th1-type response. Ultimately, the median overall survival of 15 patients reached 29 months, and the progression-free survival reached 14.2 months. One patient even survived for more than 38.9 months. In the recurrent tumor resected at 26.8 months, researchers found extensive T cell infiltration. The cancer vaccine successfully turned his "cold tumor" hot. Domestic scientists have made limited attempts in this field. In October 2018, Cancer Immunology & Immunotherapy published a Phase IIa exploratory clinical trial (NCT01567202) [31] conducted by the team of Professor Yao Yu from the Department of Neurosurgery at Huashan Hospital and Professor Chu Yiwei from the Fudan University Immunobiology Therapy Center on DC vaccine treatment for glioblastoma. This study showed that antigens formed by the lysis of A2B5+ tumor cells can elicit a strong immune response against glioma. The DC vaccine was able to increase the survival time of GBM patients, and patients with molecular characteristics such as IDH1-WT/TERT-MT and low B7-H4 expression were more likely to derive immunologic benefit. Notably, the IDH1-WT/TERT-MT molecular subtype of GBM patients, which may be more responsive to immunotherapy, is precisely the subtype that is insensitive to traditional standard treatments

and has the worst prognosis. This study brings new hope to these patients. It is reported that the Huashan Hospital Glioma Immunotherapy Team is about to launch a new RCT clinical trial to further validate the clinical efficacy of DC vaccine treatment for GBM based on molecular subtyping (Phase IIb trial).

1.4 Preliminary Work

Zhong Sheng Kang Yuan Biotechnology (Beijing) Co., Ltd. is one of the earliest institutions in China to develop personalized vaccines based on tumor neoantigens. It has established a comprehensive production and quality control system for personalized DC cell vaccines. Three patients with advanced solid tumors received a full cycle of treatment with personalized DC vaccines sensitized with tumor neoantigen peptides and entered the follow-up period. One patient with recurrent multifocal hepatocellular carcinoma after surgery and one patient with recurrent laryngeal cancer and lung metastasis after surgery both showed lesion shrinkage, achieving disease control and partial response (SD/PR) (Figure 1, Figure 2). One patient with retroperitoneal leiomyosarcoma experienced recurrence and metastasis 12 months after the first surgery, underwent a second surgery, and experienced recurrence and metastasis again 12 months after the second surgery. After conventional chemotherapy, the disease continued to progress. The patient underwent a third surgery and began treatment with a neoantigen-sensitized autologous DC vaccine 7 months post-surgery. After 4 months of observation, the lesions remained stable (SD), and T cells responding to tumor neoantigens were detected in peripheral blood (Figure 3). None of the patients receiving personalized DC vaccine treatment experienced serious adverse reactions during or after vaccination.

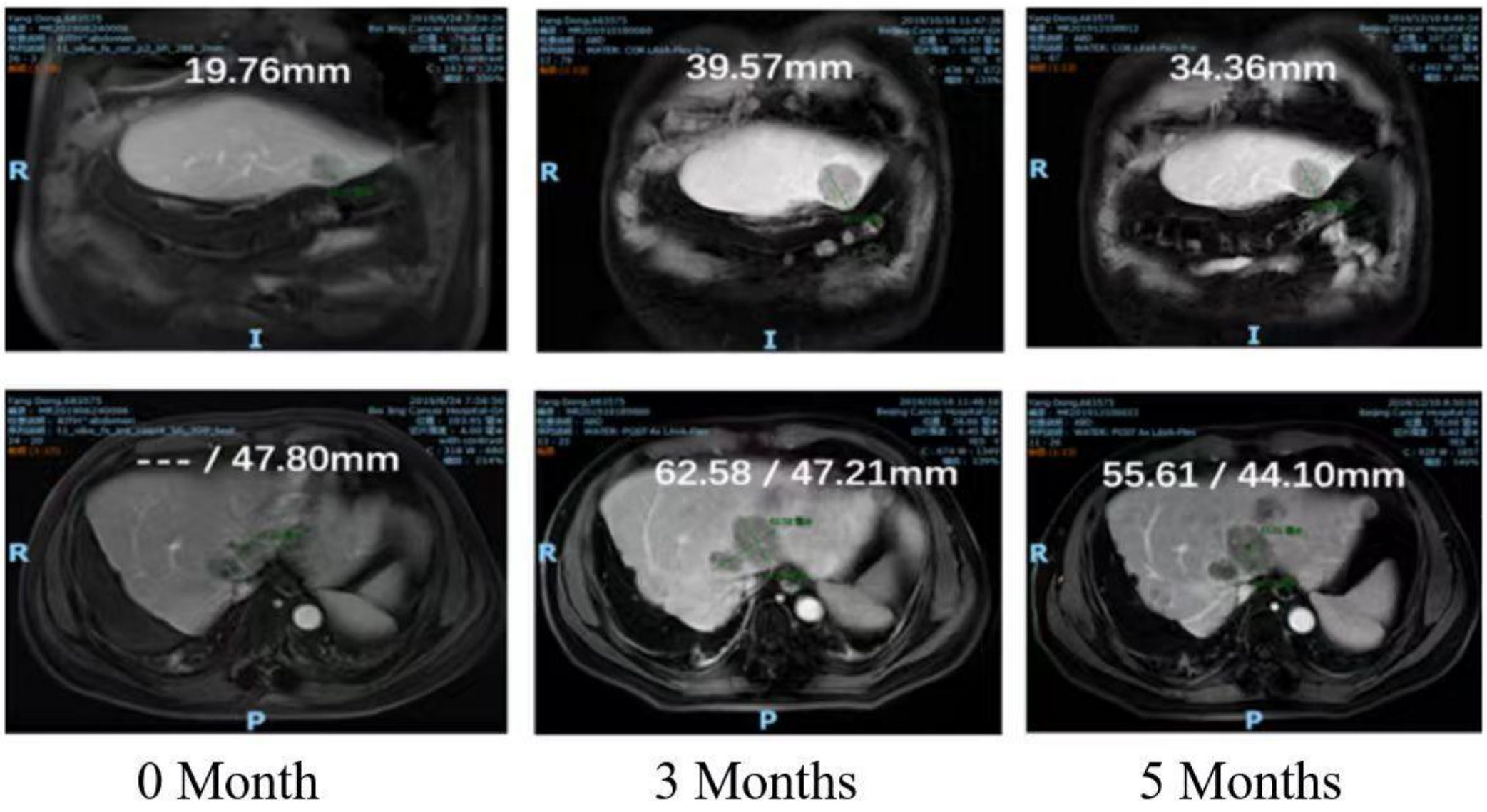

Figure 1. Imaging before and after treatment with a personalized neoantigen-pulsed DC vaccine in a patient with postoperative recurrent multifocal hepatocellular carcinoma.

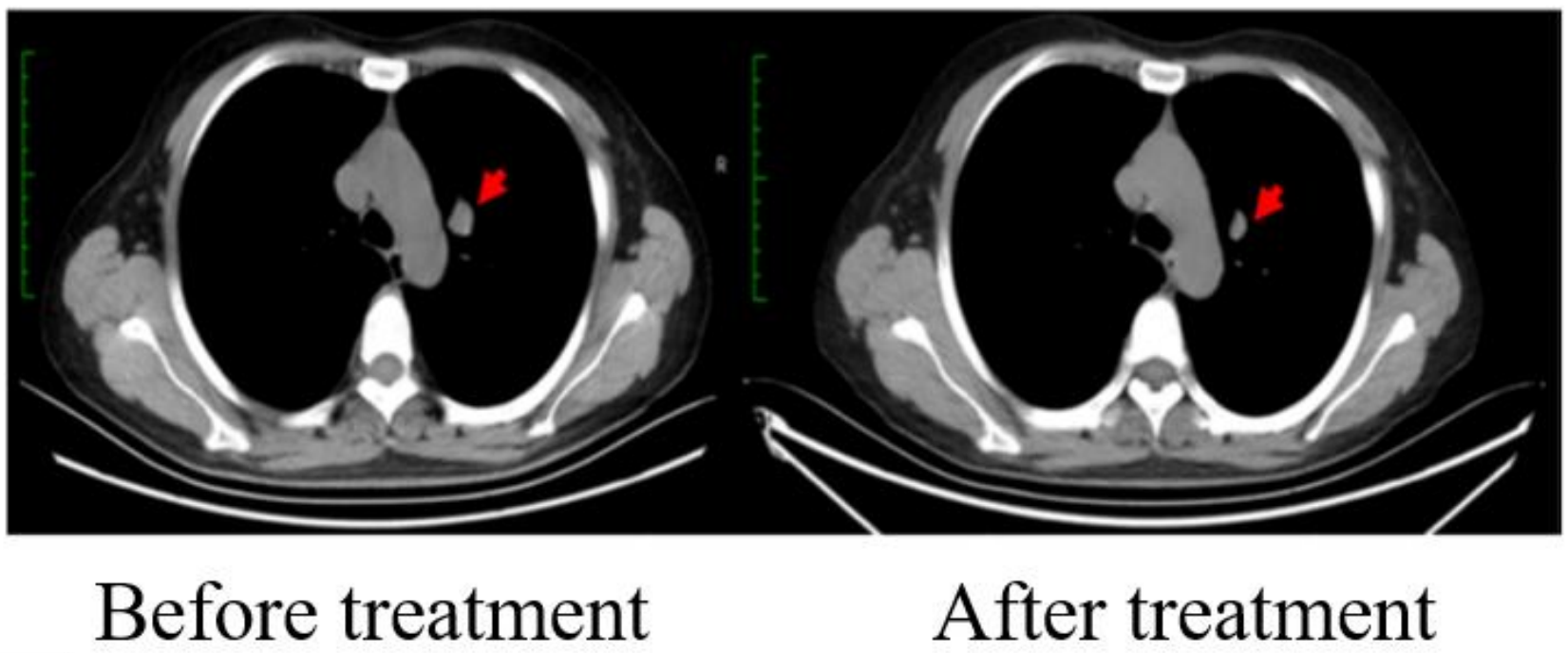

Figure 2. Imaging before and after 3 months of treatment with a personalized neoantigen-pulsed DC vaccine in a patient with postoperative recurrent laryngeal cancer with pulmonary metastases.

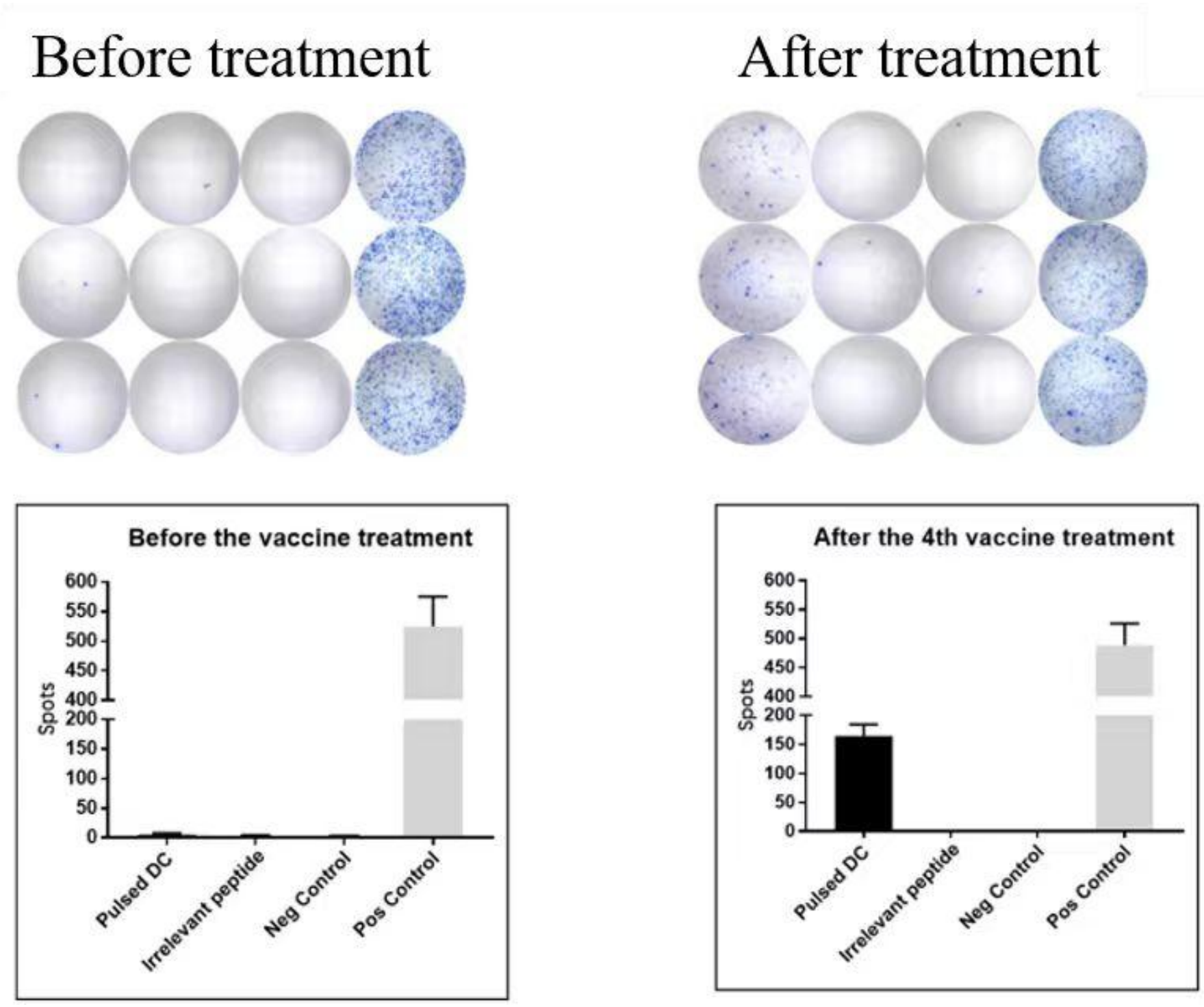

Figure 3. Detection of antigen-specific T cells in peripheral blood before and after 3 months of personalized DC vaccine therapy in a patient with retroperitoneal leiomyosarcoma.

II. Study Objectives and Rationale

2.1 Primary Objective

Evaluate the safety of personalized neoantigen-pulsed autologous DC vaccine combined with the standard TMZ treatment regimen for newly diagnosed glioblastoma.

2.2 Secondary Objectives

① Evaluate the immunologic efficacy of DC vaccine in treating newly diagnosed glioblastoma;

② To evaluate the preliminary clinical efficacy of DC vaccine in combination with standard TMZ therapy in newly diagnosed glioblastoma;

③ To analyze the correlations between immune molecular features and immune-cell phenotypes in peripheral blood and cerebrospinal fluid and the antitumor immune responses induced by the DC vaccine;

④ To evaluate the role of immune molecular features and immune-cell phenotypes in peripheral blood and cerebrospinal fluid in predicting the efficacy of DC vaccine therapy.

2.3 Significance of the Project

With advances in glioma genomics and immunology, significant progress has been made in the field of next-generation therapeutic DC vaccines for malignant brain glioma. Keskin et al. [30] found that after administration of a personalized peptide vaccine, neoantigen-specific T cells from peripheral blood can migrate to intracranial GBM tumors and produce an effective response, demonstrating the feasibility of a multi-epitope, personalized neoantigen vaccination strategy for GBM with low mutation burden and a cold tumor microenvironment. Hiff et al. [29] found that both tumor-associated antigen peptides and tumor-specific antigens showed good safety, and personalized tumor-associated antigen peptides could sustainably induce CD8+ T cell responses. At the 23rd Annual Meeting of the Society for Neuro-Oncology (SNO) in November 2018, NWBio announced data from its Phase III clinical study of DCVax®-L for newly diagnosed GBM. 33% of patients survived for 4 years or more, and 27% survived for 6 years or more. As of 2018, two patients in Phase I and II studies had survived for more than 10 years (compared to the current standard of care median survival of 14.6 months for glioblastoma). These results demonstrate the great application prospects of next-generation therapeutic DC vaccines in the clinical treatment of malignant brain glioma. However, the field is largely led by Europe and the United States, while efforts in China remain relatively limited. One important reason is the difficulty in building a tumor neoantigen discovery platform. Antigen epitope identification, vaccine design and production, and clinical research are complex multi-omics collaborative projects with high technical barriers, making them difficult to master. At the same time, malignant brain glioma exhibits severe immunosuppression and immune escape, making it difficult to accurately screen beneficiary populations. There is still a lack of in-depth research on the clinical safety and efficacy mechanisms of vaccines [33], resulting in few medical institutions in China venturing into this field.

III. Project Design

3.1 Project Overview

3.1.1 Overview

This study is a single-center, single-arm, prospective Phase I clinical study. The participant population consists of postoperative patients with newly diagnosed glioblastoma. DC vaccine treatment is administered alternately during the patients' conventional adjuvant TMZ chemotherapy. Ten participants are planned to be enrolled. Non-genetically modified autologous DC cells with high safety will be loaded with multiple tumor neoantigen peptides and administered via 5 injections into the inguinal or axillary lymph nodes and surrounding intradermal multiple sites. After completing 5 injections, the investigator will decide whether to increase the number of injections based on the participant's tolerance and compliance. In principle, the total number of injections per course should not exceed 8. For some patients with good treatment tolerance, immunological response, and clinical response, peripheral blood will be drawn after completing TMZ adjuvant chemotherapy to assess the immune response. Based on the immune response evaluation results, it will be decided whether to administer 1-2 additional courses (5-8 injections per course) of DC vaccine injections for booster treatment.

During treatment, immune responses and other clinical side effects will be closely monitored through molecular, cellular, and clinical means, with proactive management as needed. Vaccine safety will be evaluated comprehensively by monitoring adverse events, physical examination results, vital signs measurements, ECG, hematological tests, cytokine detection, etc. Vaccine efficacy will be comprehensively assessed by detecting cytokine concentrations and types, immune cell frequency and distribution, molecular characteristics of naive T-cell subsets, and other molecular biological markers and clinical biological indicators before and after treatment.

3.1.2 Reference Protocols

Pharmacology and toxicology studies are not feasible for autologous cell vaccines; therefore, existing clinical data and literature are commonly referenced. In this project, the injection site, dosing frequency, safe starting dose, permissible dose-escalation range, and

the safety and efficacy of the booster dosing interval were informed by clinical experience with similar products—particularly two successful single-arm Phase I studies of autologous DC vaccines in recurrent glioblastoma—while improvements were made in multi-epitope peptide loading, DC vaccine manufacturing processes, and intraoperative/postoperative immune monitoring techniques.

Reference Protocol 1. NCT00068510, Jonsson Comprehensive Cancer Center, Vaccine Therapy in Treating Patients With Malignant Glioma;

Reference Protocol 2. NCT01567202, Huashan Hospital, Fudan University, Study of DC Vaccination Against Glioblastoma

3.2 Project Implementation Plan

3.2.1 Implementation Plan Overview

The implementation of this project comprises four parts: vaccine preparation, clinical treatment, immune monitoring, and statistical analysis.

Vaccine preparation includes surgical sample collection of tumor tissue, genomic WES and transcriptomic RNA-seq high-throughput sequencing, bioinformatics analysis and tumor neoantigen prediction, in vitro synthesis of candidate tumor neoantigen peptides, in vitro functional validation of candidate tumor neoantigens, and preparation of autologous DC vaccines loaded with tumor neoantigen peptides.

The clinical treatment part refers to patient screening and enrollment, clinical administration of DC vaccine to patients during conventional TMZ chemotherapy, and clinical examinations during and after treatment.

Immune monitoring refers to the collection and processing of patient biospecimens, and molecular and cellular assays performed intraoperatively and postoperatively.

Statistical analysis and data review refer to the systematic analysis and organization of the clinical study data upon completion of the above steps, to evaluate the safety and efficacy of the treatment.

Overall Flowchart of the Study Protocol

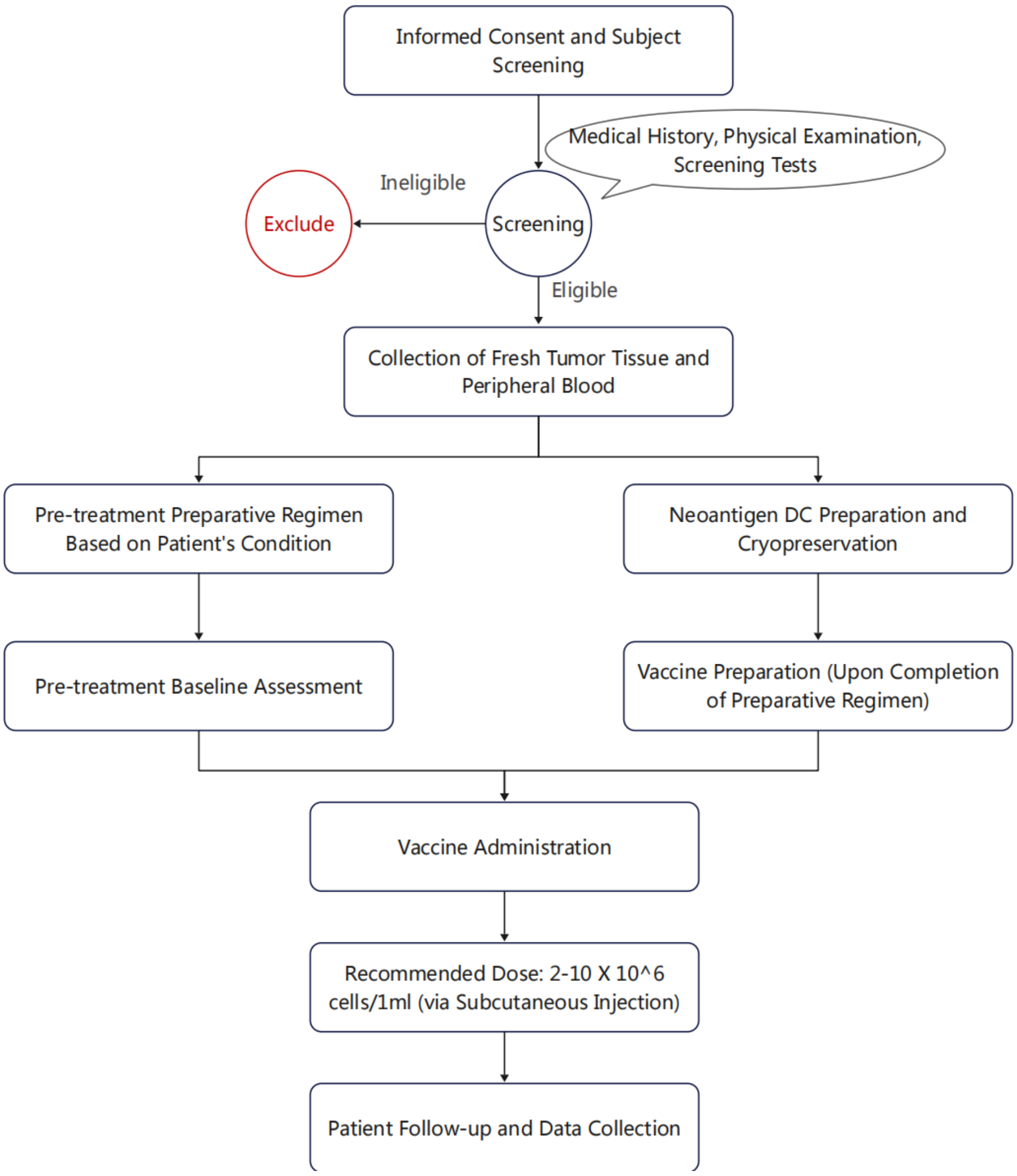

The specific project implementation process can be summarized as follows: 10 GBM patients who undergo surgical resection are determined to receive conventional postoperative radiotherapy and chemotherapy are selected. Whole exome and transcriptome high-throughput sequencing and HLA typing are performed on surgical tumor specimens and blood specimens. Tumor neoantigen epitopes are accurately predicted through a bioinformatics strategy supported by big data and artificial intelligence technology. It is verified in vitro whether the predicted epitopes have high affinity for the antigen presentation complex or whether they can effectively load patient autologous DC cells and activate CD4+/CD8+ T cells in vitro. Antigen peptides are synthesized and purified under quasi-GMP conditions using standard solid-phase peptide synthesis chemistry and reverse-phase high-performance liquid chromatography (RP-HPLC) technology (CS Bio). They are loaded onto DC cells cultured from patient mononuclear cells to prepare an autologous DC

multi-antigen vaccine. The DC vaccine is injected during TMZ treatment after completing postoperative conventional radiotherapy and chemotherapy. During treatment, immune responses and other clinical side effects are closely monitored through molecular, cellular, and clinical means, with proactive management as needed. Vaccine safety is evaluated comprehensively by monitoring adverse events, physical examination results, vital signs measurements, ECG, hematological tests, cytokine detection, etc. Vaccine efficacy is comprehensively assessed by detecting cytokine concentrations and types, immune cell frequency and distribution, molecular characteristics of naive T-cell subsets, and other molecular biological markers and clinical biological indicators before and after treatment. Simultaneously, the types, quantity, and functional status of infiltrating immune cells are analyzed to explore their correlation with anti-tumor responses and evaluate their potential as biological indicators for predicting the efficacy of personalized tumor vaccine treatment.

The neoantigen discovery/validation and cell preparation for the personalized DC vaccine are the core technical steps in the above process and will be completed through collaboration among multiple units in this clinical study. Clinical sample collection and preliminary processing will be completed by our department. High-throughput whole exome and transcriptome sequencing will be outsourced to Beijing Novogene Co., Ltd., a professional sequencing institution in China with third-party clinical testing qualifications. Quasi-GMP antigen peptide synthesis will be outsourced to GenScript Biotech Corporation. Bioinformatics prediction, immunological functional validation, immune monitoring experiments, and tumor immune microenvironment analysis will be completed by the Department of Immunology of Peking University Health Science Center in conjunction with Zhong Sheng Kang Yuan Biotechnology (Beijing) Co., Ltd. The production and preparation of the personalized DC vaccine will be completed by Zhong Sheng Kang Yuan Biotechnology (Beijing) Co., Ltd.

Flowchart of personalized DC vaccine preparation

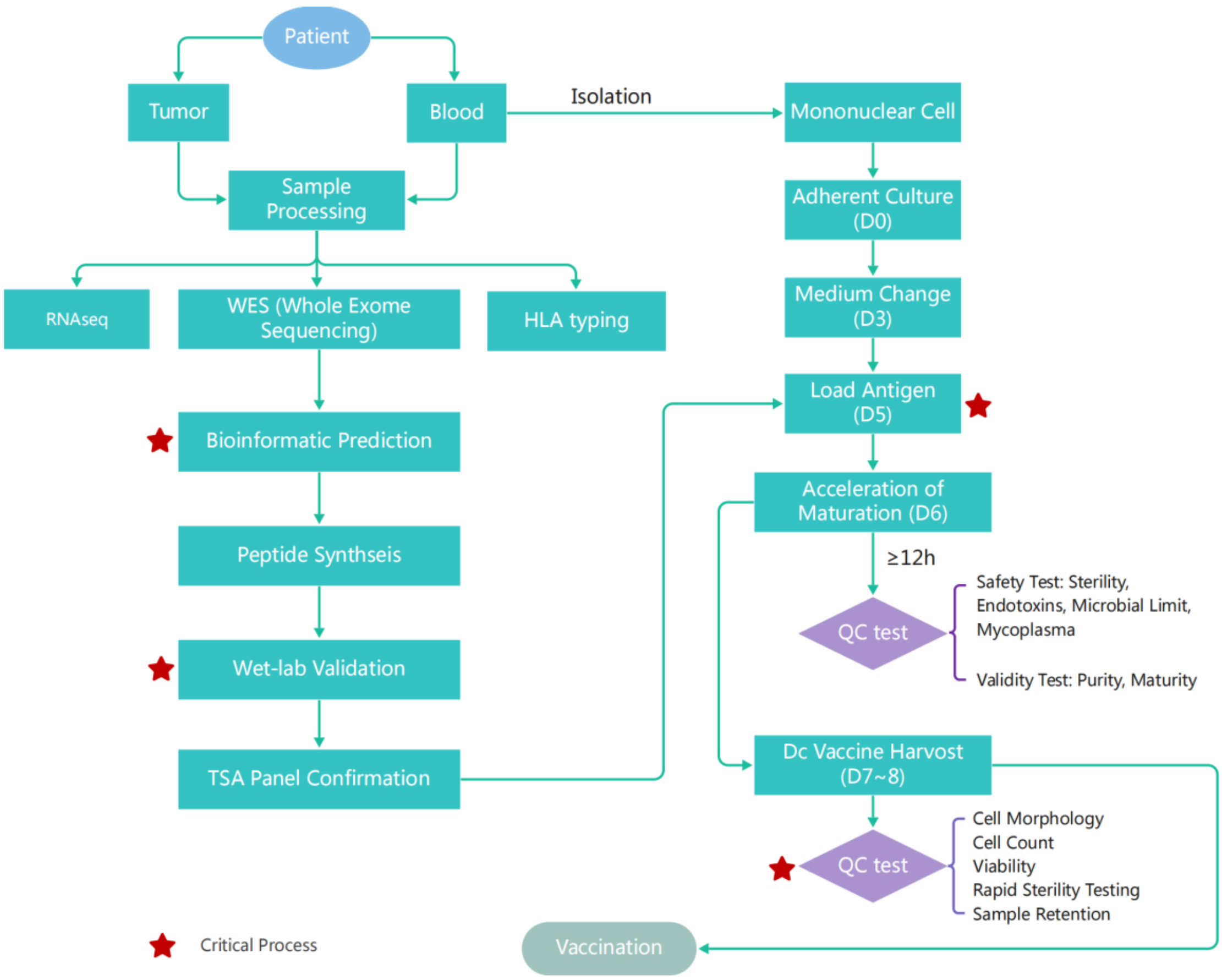

3.2.2 SOP for Collection of Tumor and Peritumoral Tissue

(1) Prepare items required for the collection of GBM tumor tissue and peritumoral tissue: liquid nitrogen containers, RNAlater preservation solution, 10% formalin, and cryotubes to be brought into the operating room. RNAlater and 10% formalin can be pre-dispensed into cryotubes and collected by a designated person.

(2) After fresh tumor tissue is excised, immediately divide it into three portions each the size of a mung bean. Rinse the surface blood off the tumor tissue with saline, and place the tumor tissue into three cryovials: take one vial and directly immerse it in liquid nitrogen, then store it in a -80°C freezer after bringing it back to the laboratory; take one vial and add 1ml RNAlater, leave it at 4°C overnight, then store it in a -80°C freezer; take one vial and add 10% formalin, immerse for 24 hours, then perform paraffin embedding.

(3) After fresh adjacent normal tissue is excised, immediately divide it into three portions each the size of a mung bean, but ensure that the operating instruments are used separately from those for tumor tissue to avoid contamination with tumor cells. Rinse

the surface blood off the tissue with saline, and place the tissue into three cryovials: take one vial and directly immerse it in liquid nitrogen, then store it in a -80°C freezer after bringing it back to the laboratory; take one vial and add 1ml RNAlater, leave it at 4°C overnight, then store it in a -80°C freezer; take one vial and add 10% formalin, immerse for 24 hours, then perform paraffin embedding.

3.2.3 Blood Sample Collection and Processing SOP

(1) For immune repertoire sequencing blood samples: Collect 1mL whole blood using an EDTA anticoagulant tube, immediately bring it back to the laboratory and add it to 4mL Trizol, disperse cell clumps or mix by shaking, let stand at room temperature for 5min, then aliquot 1ml into EP tubes and store in a -80°C freezer.

(2) For whole exome sequencing: Collect 2mL whole blood using an EDTA anticoagulant tube, immediately bring it back to the laboratory and add it to a 15ml centrifuge tube pre-filled with 6ml red blood cell lysis buffer (Solarbio R1010), mix well and place on ice for 30min (mix every 10min), centrifuge at 1600g, 4°C for 10min to obtain cell pellets, leave about 500ul liquid in the tube, resuspend and aliquot into 4 EP tubes for storage at -80°C.

(3) For immune efficacy evaluation (ex vivo enzyme-linked immunospot (ELISPOT) assay): Collect 15mL whole blood using an anticoagulant tube, immediately bring it back to the laboratory, and isolate PBMC within 3 hours. This blood sample is also used for peripheral blood cytokine analysis, immune repertoire sequencing, and immunophenotyping.

3.2.4 SOP for Prediction of Personalized Tumor Neoantigens and Peptide Synthesis

Perform deep whole exome/transcriptome sequencing and individual HLA typing on tumor tissue and blood samples using next-generation sequencing technology; after sequencing, use bioinformatics data analysis systems for tumor neoantigen prediction; based on the prediction results, synthesize and purify antigen peptides using standard solid-phase peptide chemistry and reverse-phase high-performance liquid chromatography (RP-HPLC) techniques (CS Bio). The standard flowchart is as follows:

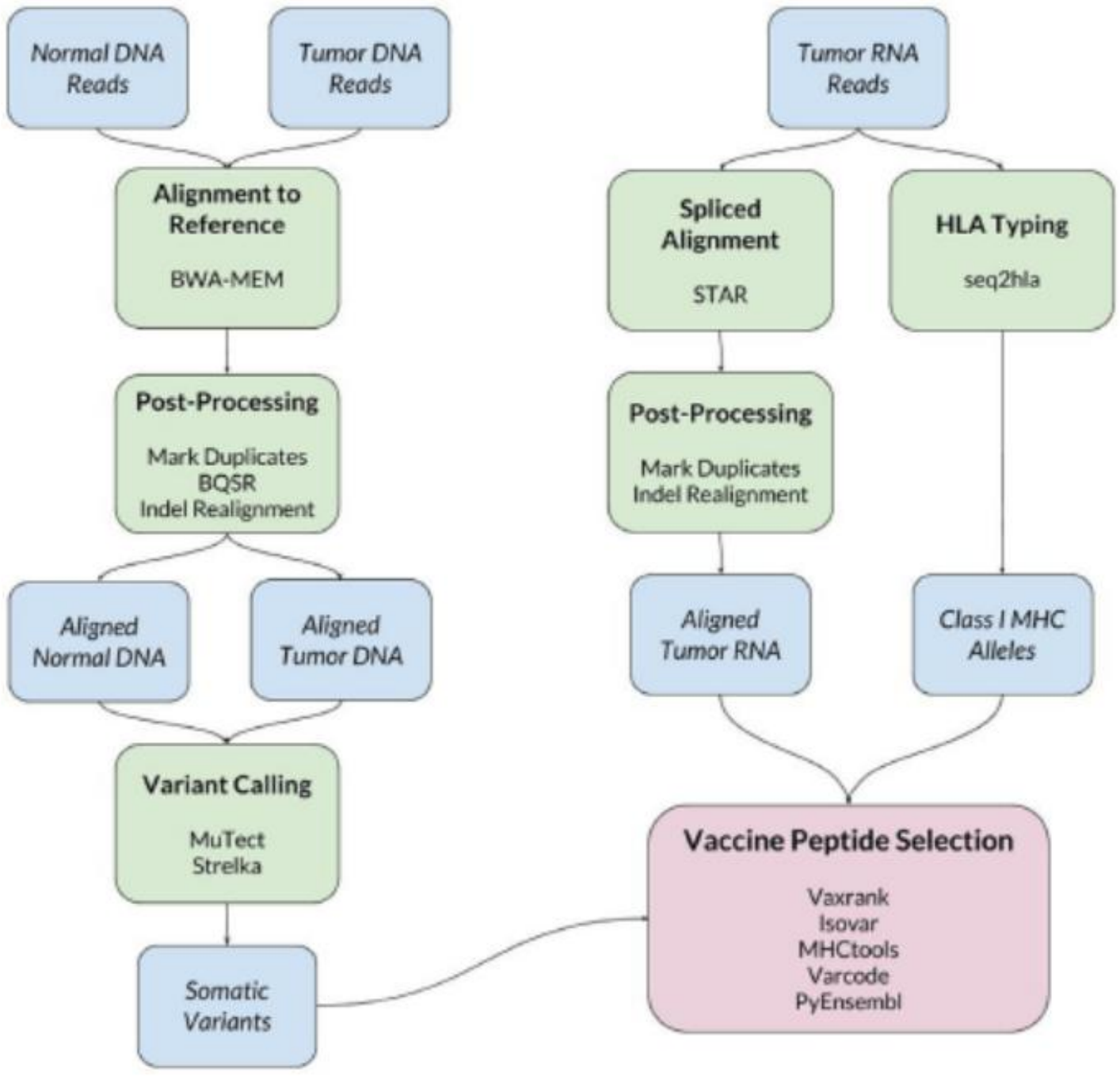

3.2.5 SOP for Preparation and Quality Control of Neoantigen-Pulsed DC

4-6 weeks after surgery, enrolled patients undergo concurrent chemoradiotherapy, after which PBMCs are collected by leukapheresis, monocytes are isolated and differentiated into DC cells in vitro, loaded with tumor neoantigen peptides, and prepared into personalized DC vaccine.

3.2.6 Pre-treatment Regimen

During the preparation process, whether pre-treatment is administered to the subject depends on the physician's assessment of the condition, tumor burden, and peripheral blood leukocyte count. After blood collection and before treatment, pre-treatment is administered 3±1 days before treatment, so that cell reinfusion can be performed 2-3 days after pre-treatment ends.

3.2.7 Pre-treatment Baseline Assessment

Baseline assessment is conducted based on established postoperative and post-conventional chemoradiotherapy clinical examination results, including tumor burden, complete blood count, etc.

3.2.8 Dosage and Administration

Each dose of DC vaccine contains 2-10×10^6 DC cells loaded with 5-20 tumor neoantigens, with a volume of 1ml, administered via multiple intradermal injections in the

groin or axillary lymph nodes and surrounding areas. Vaccination time points are at weeks 3, 4, 5, 8, and 12 after concurrent chemoradiotherapy, totaling five times per course (see study flowchart for details). After completing 5 injections, the investigator will decide whether to increase the number of injections based on the subject's tolerance and compliance. in principle, the total number of injections per course should not exceed eight.

For some subjects with good treatment tolerance, immunological response, and clinical response, peripheral blood is drawn after completing TMZ adjuvant chemotherapy to assess immune response. Based on the immune response evaluation results, it is decided whether to administer an additional 1-2 courses (5-8 injections per course) of DC vaccine injections for booster therapy.

3.2.9 Immune Monitoring Plan

During and after treatment, collect venous blood and/or cerebrospinal fluid samples from multiple time points (see figure below). Use the ELISPOT method to evaluate peripheral blood anti-tumor immune response; simultaneously perform cytokine detection in peripheral blood and cerebrospinal fluid, immune repertoire sequencing, and analysis of various immune cell compositions. Monitor dynamic changes in the patient's peripheral blood and cerebrospinal fluid immunity, analyze changes in the quantity and functional status of immune cell subsets, and multi-dimensionally analyze the correlation between actually induced anti-tumor immune response neoantigen epitopes and the therapeutic effect of the neoantigen vaccine. Analyze the composition, characteristics, and changes of immune cells and cytokines in the patient's peripheral blood and cerebrospinal fluid, and comprehensively evaluate the safety and clinical efficacy of the DC vaccine in treating glioblastoma.

DC Vaccine Injection and Immune Monitoring Plan Diagram

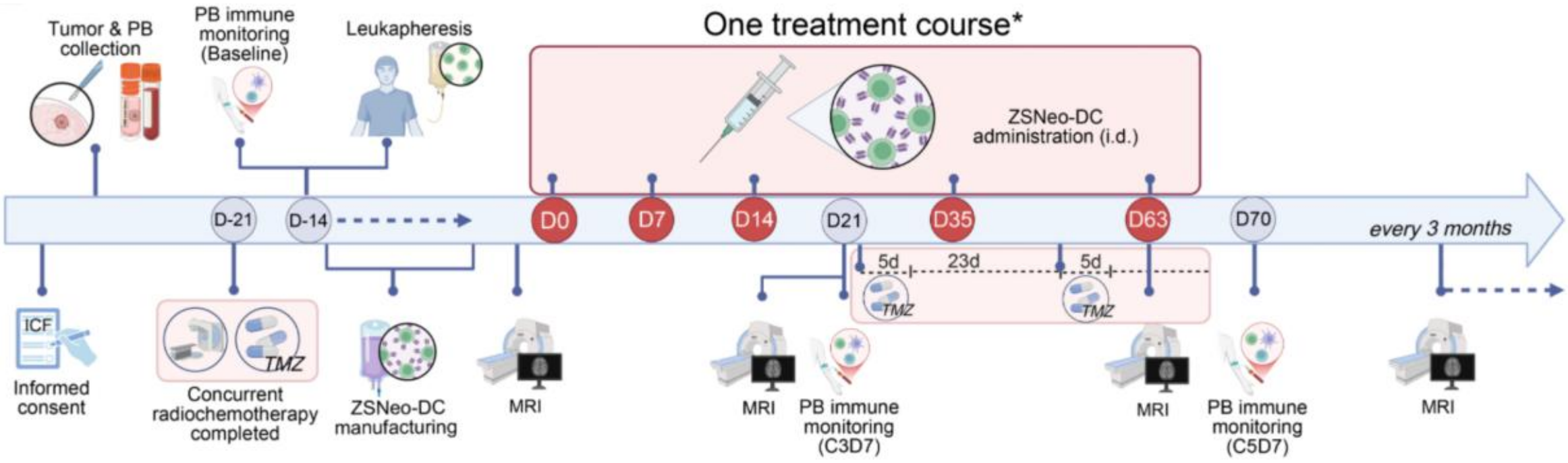

* Five injection cycles constitute one treatment course; up to three additional injection cycles are allowed within a single course and multiple treatment courses are permitted.

3.2.10 24 Hours After Vaccine Injection

Full preparations should be made to address allergic reactions, hypotension, or other side effects. Vital signs (temperature, respiration, pulse, oxygen saturation, and blood pressure) should be recorded before and after vaccination. After vaccination, record every 15 minutes for at least 1 hour, or until the condition stabilizes. Observe in the hospital for 24 hours after vaccination. If no significant side effects occur, discharge may be decided by the physician. If side effects occur, discharge may be decided by the physician 48 hours after the side effects subside.

3.2.11 Infection and Other Toxic Reactions

In very rare cases, sepsis or bacteremia may occur. If detected, blood cultures should be immediately performed and appropriate medications administered, and the sterility of the trial vaccine should be rechecked using retained samples. If the investigator believes the following toxic reactions may be related to treatment, the therapeutic vaccine should be dose-reduced, delayed, or permanently discontinued:

Toxicity	Treatment Hold Criteria for the Following Grades	Time to Resume Treatment	Discontinue Medication
Diarrhea/Colitis	2-3	Toxicity resolved to Grade 0-1	Toxicity not resolved within 8 weeks after the last dose, or unable to reduce corticosteroids to ≤10mg per day (prednisone equivalent) within 8 weeks
	4	Permanently discontinue treatment	Permanently discontinue treatment
AST/ALT/Bilirubin Elevation	2	Toxicity resolved to Grade 0-1	Toxicity not resolved within 8 weeks after the last dose
	3-4	Permanently discontinue treatment	Permanently discontinue treatment
Hyperglycemia	3-4	For newly diagnosed type I diabetes or Grade 3-4 hyperglycemia, treatment should be held	Treatment can be resumed after the patient's clinical or metabolic condition stabilizes

Hypophysitis	2-4	Toxicity resolved to Grade 0-1. Treatment can continue after initiating endocrine replacement therapy	Toxicity not resolved within 8 weeks after the last dose, or unable to reduce corticosteroids to ≤10mg per day (prednisone equivalent) within 8 weeks
Hyperthyroidism	3	Toxicity resolved to Grade 0-1	Toxicity not resolved within 8 weeks after the last dose, or unable to reduce corticosteroids to ≤10mg per day (prednisone equivalent) within 8 weeks
	4	Permanently discontinue treatment	Permanently discontinue treatment
Hypothyroidism		Treatment can continue after initiating levothyroxine replacement therapy	Treatment can continue after initiating levothyroxine replacement therapy
Infusion Reaction	3-4	Permanently discontinue treatment	Permanently discontinue treatment
Pneumonitis	2	Toxicity resolved to Grade 0-1	Toxicity not resolved within 8 weeks after the last dose, or unable to reduce corticosteroids to ≤10mg per day (prednisone equivalent) within 8 weeks
	3-4	Permanently discontinue treatment	Permanently discontinue treatment
Renal Failure or Nephritis	2	Toxicity resolved to Grade 0-1	Toxicity not resolved within 8 weeks after the last dose, or unable to reduce corticosteroids to ≤10mg per day (prednisone equivalent) within 8 weeks
	3-4	Permanently discontinue treatment	Permanently discontinue treatment
All Other Drug-Related Toxicities	3	Toxicity resolved to Grade 0-1	Toxicity not resolved within 8 weeks after the last dose, or unable to reduce corticosteroids to ≤10mg per day (prednisone equivalent) within 8 weeks
	4	Permanently discontinue treatment	Permanently discontinue treatment

IV. Case Selection

4.1 Screening Records

Investigators must record all patients who undergo screening, including those who fail screening, and keep the records in the research center's document system. This is essential to ensure unbiased selection of the subject population.

4.2 Inclusion Criteria

Subjects must meet all of the following criteria to be enrolled:

1) Male or female aged 18-75 years (inclusive);
2) Newly diagnosed glioblastoma patients confirmed by histopathology (WHO Grade 4);
3) Molecular pathology diagnosis confirms wild-type IDH1 and IDH2 genes;
4) Patients with >90% resection of the enhanced tumor lesion;
5) KPS score ≥ 60 before DC vaccine treatment;
6) Normal bone marrow reserve, normal liver and kidney function, and normal cardiac function before DC vaccine treatment:
   - Absolute leukocyte count ≥ 2.5×10^9/L;
   - Hemoglobin > 100 g/L;
   - Platelet count > 100×10^9/L;
   - ALT, AST < 2.5 x ULN;
   - Serum creatinine < 1.5 x ULN.

4.3 Exclusion Criteria

1) Concomitant with other uncontrolled malignancies;
2) Received carmustine wafer implantation within 6 months;
3) Active hepatitis B or C, or HIV infection;
4) Grade 2-3 hypertension with poor blood pressure control;
5) Patients with severe cardiovascular or cerebrovascular diseases such as coronary heart disease, angina, myocardial infarction, arrhythmia, cerebral thrombosis, or cerebral hemorrhage;
6) Any other uncontrolled active disease that hinders participation in the trial, such as

hemolytic anemia, psoriasis, rheumatoid arthritis, or other autoimmune skin diseases;

7) History of poorly controlled psychiatric disorders, or subjects whose medical or psychiatric history, in the investigator's opinion, may increase the risk associated with study participation or study drug administration, or may interfere with results;
8) Long-term use of immunosuppressants due to reasons such as organ transplantation;
9) Systemic use of high-dose steroids (>10mg/d prednisone or equivalent) within 4 weeks before autologous DC vaccine application, excluding recent or current inhaled corticosteroid therapy;
10) Unstable pulmonary embolism, deep vein thrombosis, or other major arterial/venous thromboembolic events occurring within 30 days before enrollment, or undergoing anticoagulant therapy;
11) Subjects who are pregnant or breastfeeding, or plan to become pregnant during the treatment period or within 2 months after the end of treatment;
12) Active infection requiring systemic treatment or uncontrolled infection (excluding simple urinary tract infections or upper respiratory tract infections) within 14 days before enrollment;
13) Patients who have received other vaccine therapies or other genetically modified cell therapies before enrollment;
14) After tissue sample sequencing and analysis, the predicted neoantigen peptides are fewer than 5;
15) Individuals deemed unsuitable for this trial by the investigator;
16) Subjects with conditions affecting the ability to provide written informed consent or comply with study procedures; or subjects unwilling or unable to comply with study requirements;
17) Subjects who have participated in other drug clinical trials within 4 weeks before enrollment or are currently participating in other drug clinical trials.

4.4 Subject Completion/Non-Completion of the Study

4.4.1 Completion

Subjects who discontinue treatment due to disease progression or death are considered to have completed the study.

4.4.2 Treatment Discontinuation

Subjects have the right to discontinue this study at any time during the study, and their decision will not affect the treatment of their disease. Subjects should discontinue the study if any of the following situations occur:

(1) The investigator believes that discontinuing treatment is in the best interest of the subject for safety reasons (e.g., adverse events);

(2) The subject becomes pregnant;

(3) The subject experiences disease progression;

(4) Death;

(5) Complications preventing continued medication;

(6) Insufficient quantity of DC cells obtained for vaccine preparation;

(7) Protocol violation occurs, e.g., treatment with other antitumor drugs;

(8) Subject request: Subjects have the right to withdraw from this project at any time for any reason. The reason for withdrawal should be recorded in the CRF form.

(9) Poor compliance, and the investigator deems the subject unsuitable to continue the study;

(10) Administrative reasons: Any reason leading the investigator, sponsor, ethics committee, or relevant regulatory authorities such as the Health and Family Planning Commission/Drug Administration to determine that the project must be terminated.

Upon treatment discontinuation, if the subject requires another systemic antitumor drug, all steps required at the end of the treatment visit should be completed as soon as possible after withdrawal from the study. Subjects who discontinue treatment due to efficacy remain candidates for long-term follow-up observation, unless the subject refuses to continue any study-related procedures or further efficacy evaluation is not possible due to major protocol deviations (e.g., use of other antitumor drugs). The reason for treatment discontinuation must be recorded in the CRF.

4.4.3 Study Withdrawal

Subjects may withdraw from the study for the following reasons:

(1) Lost to follow-up;

(2) Withdrawal of informed consent.

If a subject is lost to follow-up, the investigator must make every effort to contact the subject to determine the reason for withdrawal. All follow-up measures taken must be recorded. When a subject withdraws from the study before its completion, the reason for withdrawal should be recorded in the original records. The final safety and efficacy evaluation will be based on the time point of withdrawal.

V. Study Evaluation

5.1 Primary Endpoints (Safety Assessment)

This project uses NCI CTCAE 5.0 to grade adverse events (AEs) and serious adverse events (SAEs). The investigator must monitor the safety of all subjects enrolled and receiving at least one dose of DC vaccine, recording any AEs and SAEs. Observe any toxicity, immune responses, and long-term malignant events. The investigator must provide appropriate medical care to patients during the project implementation and follow up on any SAEs or AEs leading to treatment discontinuation through appropriate healthcare measures. Follow-up should continue until the AE/SAE resolves or is reasonably explained. The investigator may determine the frequency of follow-up at their discretion.

(1) AE: Any unfavorable medical event occurring in a subject after enrollment and receiving at least one dose of DC vaccine, regardless of whether it is related to this project. Thus, an AE can be any unfavorable sign, symptom, or disease temporally associated with the use of the drug but not necessarily causally related. Worsening of pre-existing conditions during the project is also considered an adverse event. The investigator will record any changes in condition and the occurrence and nature of AEs, including clinically significant signs and symptoms of disease that emerge during treatment.

(2) SAE: Collection of SAEs begins after the subject receives the investigational product, including new SAEs occurring within 8 weeks after treatment discontinuation. An SAE is defined as any event meeting one of the following criteria:

A. Death (excluding death due to disease progression, unless the investigator considers it related to the treatment in this project);

B. Requires hospitalization or prolongs existing hospitalization (excluding: ① hospital stay ≤12 hours; ② surgery or elective surgery scheduled before the start of this project; ③ hospitalization unrelated to AE);

C. Life-threatening (AE that could directly lead to the subject's death);

D. Permanent or significant disability, or incapacity;

E. Significant medical event.

(3) AE Recording and Reporting

A. All AEs occurring during the project period after receiving at least one dose of DC vaccine must be recorded in the CRF (Case Report Form). AEs occurring more than 8 weeks after the last dose must also be recorded in the CRF if they are related to the treatment in this project.

B. If any of the above AEs meet the criteria for an SAE, the investigator should report it to the sponsor via telephone and fax within 24 hours of becoming aware of the SAE.

C. After submitting the initial SAE report, if important follow-up information is obtained, the investigator should complete a follow-up SAE form and submit it to the above units. In case of death, the investigator should write a detailed death case description on the SAE report form.

D. Any new SAEs occurring within 8 weeks after the last treatment should be reported as described above. For SAEs beyond this period, if the investigator considers them related to the immune cells used, the investigator is responsible for reporting the event to the sponsor and relevant authorities in any form.

(4) Emergency response plan for adverse reactions occurring during the project: Refer to the "Standard Operating Procedure for Emergency Plans in Oncology Drug Clinical Trials."

A. Purpose: The clinical application of immune cell therapy involves systemic clinical use of immune cell therapy in humans to determine the safety and efficacy of this immune cell therapy technology. It carries certain dangers and risks. To ensure subject safety and provide timely treatment in case of danger during the treatment process, this operating procedure is established.

B. Scope: Applicable to the safety assurance of subjects in the clinical application of immune cell therapy in this specialty.

C. Responsibilities: The sponsor and investigator are responsible for the implementation of this specification.

D. "Administrative Measures for the Clinical Application of Medical Technologies" issued by the National Health and Family Planning Commission of the People's Republic of China.

(5) Rescue Procedure Content

A. Establish a dedicated clinical emergency medical team; the project leader serves as the team leader, with investigators and specialized nurses as main members. Consult relevant professionals for assistance in rescue operations.

B. In the event of a serious adverse event, key members of the emergency team must be present, and the team leader will direct the rescue and coordination efforts.

C. In case of critical illness in the ward, immediately transfer the subject to the emergency room for rescue.

D. The emergency room is equipped with dedicated oxygen suction, sputum suction, emergency medications, and ECG monitoring equipment.

E. In the event of critical illness, rescue measures should be taken first, followed by reporting to the investigator, project leader, clinical monitor, pharmacological institution, institutional ethics committee, and sponsor as soon as possible.

F. The specific rescue work should be led by an investigator with the qualifications of attending physician or above, and the progress of the condition should be reported to the project leader promptly.

G. For serious adverse events, the time of occurrence, treatment plan, outcome after treatment, and disease progression must be recorded in detail; and archived and stored together with various inspection reports and CRF during the rescue period.

H. Complete the adverse event or serious adverse event report form. Serious adverse events must be reported to relevant institutions in accordance with regulations and handled accordingly.

I. The investigator should complete the "Initial Serious Adverse Event Report Form."

J. The investigator should report the serious adverse event to the sponsor via the fastest means (e-mail or telephone) within 24 hours of becoming aware of it.

5.2 Secondary Endpoints (Efficacy Assessment)

Immune efficacy assessment: Before DC vaccine injection (at the time of apheresis), and one week after the 3rd, 5th, and the last injection of each course (if more than 5 injections), use ex vivo enzyme-linked immunospot (ELISPOT) assay to detect the number of subjects with specific IFN-γ+ T cells in peripheral blood PBMCs after processing with their own tumor neoantigens (positive defined as at least 55 antigen-specific spot-forming cells (SFC) per 10^6 PBMCs or a ≥3-fold increase from baseline).

Clinical efficacy assessment: After individualized DC vaccine treatment for patients with newly diagnosed glioblastoma post-surgery, use iRANO criteria to evaluate treatment efficacy, record progression-free survival (PFS, defined as the time from surgery to tumor recurrence or progression), overall survival (OS, defined as the time from surgery to patient death), 1-year survival rate (defined as the proportion of all patients receiving DC treatment with OS exceeding 1 year), and quality of life assessment (QOL); if measurable lesions exist, evaluate short-term treatment efficacy (CR, PR, PD, SD), disease control rate (DCR), and clinical benefit rate (CBR), etc.; observe changes in the immune microenvironment during and after treatment, and analyze their correlation with vaccine clinical efficacy.

Analyze the correlation between immune molecular characteristics of peripheral blood and cerebrospinal fluid and immune cell phenotypes with antitumor immune responses.

Evaluate the role of immune molecular characteristics of peripheral blood and cerebrospinal fluid and immune cell phenotypes in predicting the efficacy of tumor DC vaccine treatment.

5.3 Clinical Monitoring Parameters and Assessment Schedule

5.3.1 Vital Signs Monitoring

Monitor vital signs before and after vaccination until the condition stabilizes.

5.3.2 Toxicity Assessment Indicators

Complete blood count, urinalysis, blood biochemistry, coagulation routine, immune cell typing, serum Ig levels, cytokine detection, and electrocardiogram, to be tested once on

the day before vaccination and on days 1, 7, 14, 35, and 63 after the (first) vaccination. Specific test items are detailed in Attachment 7 "Study Flowchart."

For subjects receiving DC vaccine booster therapy after TMZ chemotherapy, perform the above toxicity indicator tests once during and at the end of each course.

5.3.3 Immune Efficacy Indicators

Before DC vaccine injection (at the time of apheresis), and one week after the 3rd, 5th, and the last injection of each course (if more than 5 injections), use ex vivo enzyme-linked immunospot (ELISPOT) assay to detect the number of subjects with specific IFN-γ+ T cells in peripheral blood PBMCs after processing with their own tumor neoantigens (positive defined as at least 55 spot-forming cells (SFC) per 10^6 PBMCs or a ≥3-fold increase from baseline).

For some subjects with good treatment tolerance, immune response, and clinical response, draw peripheral blood after completing TMZ adjuvant chemotherapy to assess immune response. Based on the immune response evaluation results, decide whether to administer 1-2 additional courses (5-8 injections per course) of DC vaccine injections for booster therapy. Also, draw blood once during and at the end of each course (15 mL each time) for immune efficacy assessment.

5.3.4 Other Immune Response Monitoring Indicators

Using the peripheral blood from the above ELISPOT assay, cytokine detection, immune repertoire sequencing, and analysis of various immune cell compositions are performed simultaneously; 3 mL of cerebrospinal fluid is collected before the 5th injection to complete cytokine detection, immune repertoire sequencing, and analysis of various immune cell compositions. These indicators can comprehensively evaluate the safety and immune efficacy of DC vaccines in the treatment of glioblastoma on one hand, and on the other hand, be used to discover immune indicators that predict the efficacy of DC vaccines.

5.3.5 Clinical Efficacy Evaluation

For patients, clinical efficacy is evaluated using the iRANO criteria based on cranial MR (plain scan + contrast + FLAIR + DWI) examinations combined with clinical responses. Evaluations are conducted during the enrollment period and after the 5th vaccine injection; thereafter, follow-up assessments are performed every 3 months, with the date of the clinical

efficacy evaluation after the 5th vaccine injection as the reference. For details, refer to Appendix 7 "Study Flowchart."

5.4 Quality of Life Assessment

The FACT-G scale (EORTC QLQ-30 Quality of Life Scale) is used to assess quality of life. Evaluations are conducted during the enrollment period; on the 7th, 14th, 35th, and 63rd days after the first immunotherapy; and thereafter every 3 months, with the date of the clinical efficacy evaluation after the 5th vaccine injection as the reference. For details, refer to Appendix 7 "Study Flowchart."

VI. Adverse Reactions and Treatment Recommendations

6.1 Possible Adverse Reactions (Adverse Reaction Record Form)

Classical chemotherapeutic drugs are mostly cell metabolism or cell cycle inhibitors. In addition to killing tumor cells, they non-specifically cause significant damage to normal tissues and cells with high growth and metabolic activity, such as the hematopoietic system, liver, and kidneys. Radiotherapy also damages normal tissues and organs. In contrast, reinfusing dendritic cells loaded with tumor antigens or tumor-DC fusion cells into patients can avoid these toxic reactions or side effects. These cells originate from the patient and return to the patient, primarily inducing a tumor-specific immune response in the body and enhancing the intensity of this response.

DC vaccines mainly consist of two components: dendritic cells and tumor antigens. In terms of cells, the DCs used are derived from the patient's own peripheral blood or induced and differentiated from the patient's own monocytes, thus avoiding rejection due to HLA mismatches. In terms of antigens, the new generation of personalized DC vaccines use tumor-specific neoantigens resulting from gene mutations in the patient's tumor cells. The induced specific immune response is strong but tumor-cell-specific, avoiding cross-reactions and autoimmune reactions against the patient's own normal components. No autoimmune responses have been observed in extensive clinical studies. Additionally, in the preparation process, the new generation of DC vaccines require a strict quality management system, eliminating the use of animal serum as a xenogeneic protein component in

traditional DC vaccine cultures. This prevents allergic reactions in patients with allergic constitutions. Based on the clinical application of tumor neoantigen-sensitized DCs in recent years, this approach is well-tolerated in almost all patients, and treatment can be administered directly on an outpatient basis.

According to the toxicity evaluation criteria established by the WHO, no significant side effects or toxic reactions have been observed in clinical trials of DC tumor vaccines. The more common side effects include moderate fever and local redness and swelling at the injection site for subcutaneous immunization, which subside within 2 days after immunization. No liver, kidney, or hematopoietic system dysfunction related to DC treatment has been observed. For autoimmune reactions, high titers of autoantibodies have been detected in a small number of patients, but no clinical symptoms of autoimmune diseases or functional changes in affected organs have occurred. Clinical trial results indicate that autologous DC tumor vaccines are a relatively safe tumor immunotherapy.

Generally, the possible adverse reactions in immune cell therapy include the following:

① Chills, fever.

② Cytokine release syndrome (CRS).

③ Loss of appetite, nausea, vomiting, abdominal pain.

④ Fatigue, sweating, rash.

⑤ Hypotension, arrhythmia, tachycardia.

⑥ Shortness of breath, dyspnea, acute respiratory distress syndrome.

⑦ Abnormal neurological status, such as headache, delirium, confusion, convulsions, etc.

⑧ Coagulation abnormalities, disseminated intravascular coagulation.

⑨ Capillary leak syndrome, such as systemic edema, hypoproteinemia, pulmonary edema.

⑩ Polyserositis effusion, such as pleural effusion, ascites, pericardial effusion.

11 Peripheral neuropathy, such as limb pain, paresthesia, sensory impairment.

12 Tumor lysis syndrome.

13 Infection.

14 Bone marrow suppression, such as leukopenia, thrombocytopenia, etc.

15 Abnormal liver and kidney function.

16 Monocyte activation syndrome.

17 Reduced B-cell count in peripheral blood and hypoglobulinemia.

18 Uncontrolled expansion of T cells in the body.

19 Abnormal expansion of viral vectors in the body, inducing mutations and causing other types of tumors.

20 Disease progression.

21 Multiple organ dysfunction syndrome.

In a previous "Single-Arm Phase I/II Clinical Study on the Safety and Efficacy of Personalized DC Vaccine Therapy for Advanced Solid Tumors" conducted by Zhong Sheng Kang Yuan Biotechnology (Beijing) Co., Ltd., the technology provider for this clinical study, at the Hepatobiliary and Pancreatic Surgery Department of Peking University Cancer Hospital, 8 patients received DC vaccine treatment. Among them, 6 occasionally experienced moderate fever not exceeding 38.5°C after DC vaccine injection, which subsided within 12 hours. Three patients had mild local skin redness and swelling after DC vaccine injection, which disappeared within 24 hours. All patients had normal blood routine, biochemistry, coagulation, myocardial enzymes, thyroid function, liver function, kidney function, and amylase before and after each injection, and no severe discomfort was observed after injection or during the treatment cycle.

In this clinical study, if subjects exhibit the above symptoms, symptomatic treatment will be provided. For example, for fever and chills, ibuprofen, propacetamol, or Xin Huang tablets can be administered every 4-6 hours, along with blood culture to rule out bacterial infection. Hormones should be used with caution. Headaches can be treated with paracetamol and neurological examinations. Nausea and vomiting can be managed with antiemetics such as metoclopramide, diphenhydramine, or tropisetron. Hypotension can be controlled with fluid infusion, and if uncontrolled, vasopressors can be used. Oxygen therapy is used for hypoxia, and if uncontrolled, a ventilator may be required. Corticosteroids are administered for abnormal T-cell expansion.

6.2 Diagnosis and Treatment Recommendations for Cytokine Release Syndrome (CRS)

The mechanism of action of personalized DC immunotherapy loaded with patient tumor neoantigens involves delivering autologous DC cells to lymph nodes, naturally activating and expanding effector T cells carrying TCRs (antigen recognition receptors) that match the tumor neoantigens. This immune activation requires time to establish an immune response, which then translates into long-term clinical effects. Autologous DC immunotherapy is mild and slow-acting, and theoretically does not induce CRS reactions in the body.

Although large-scale clinical studies worldwide have not observed CRS reactions induced by personalized DC vaccines, this study has also prepared countermeasures for any potential occurrences: The clinical symptoms of CRS observed in T-cell therapy are similar to mild flu-like symptoms in most patients. This typically involves high fever for several days, during which the possibility of infection causing the fever should be ruled out. High fever may appear suddenly and be accompanied by other clinical symptoms such as fatigue, anorexia, nausea, vomiting, diarrhea, myalgia, arthralgia, headache, rash, hypotension (usually requiring vasopressors), shortness of breath, hypoxia (requiring a ventilator), delirium and confusion (in a few patients), disseminated intravascular coagulation, and macrophage activation syndrome (MAS). Patients with severe CRS (Grade 3 or above) require ICU monitoring. Cytokines that may significantly increase in patients include IL-6, TNF-α, IFN-γ, IL-10, IL-2, IL-2R, MCP-1, MIP-1β, etc. In some cases, changes in transaminases (similar to acute liver failure) and coagulation disorders may also occur. Severe CRS can lead to macrophage activation, causing macrophage activation syndrome (MAS). MAS is an immune activation reaction and one of the outcomes of CRS, so it can be considered a symptom of CRS. MAS is similar to HLH (Hemophagocytic Lymphohistiocytosis; also known as Hemophagocytic Syndrome, HPS). HLH is caused by immune stimulation such as infection or autoimmunity but is different from familial or genetic HLH. There are currently no diagnostic guidelines for MAS, but guidelines for HLH can be used. The clinical symptoms of HLH include persistent high fever, cytopenia in at least 2-3 cell lines, and hepatosplenomegaly. It is also accompanied by abnormal

biochemical indicators, such as elevated circulating ferritin, soluble IL-2 receptor (sCD25), and triglyceride levels, as well as reduced circulating NK cell activity. Additionally, there may be elevated transaminases (similar to acute liver failure) and coagulation disorders (e.g., disseminated intravascular coagulation). A hallmark of MAS is the presence of CD163+ hemophagocytic macrophages in bone marrow and lymph node aspirates. Some patients may experience neurological symptoms such as delirium and seizures. These symptoms may also be caused by CRS or MAS. In most cases, these symptoms resolve after CRS treatment. Based on this, we propose

Table 1: CRS Grading and Treatment Recommendations:

Table 1. Cytokine Release Syndrome (CRS) Grading and Treatment Recommendations

Grade	Clinical Manifestations	Recommended Treatment Measures
Grade 1	Mild reactions, such as fever, nausea, vomiting, sweating, rash, fatigue, exhaustion, myalgia, etc.	Antipyretics, analgesics, and antiemetics may be administered, with close observation.
Grade 2	Symptoms require moderate intervention. Hypotension (responsive to fluid infusion or a single low-dose vasopressor), hypoxia (oxygen concentration <40%), Grade 2 organ toxicity.	Close monitoring of cardiac and other organ functions; intervention required, such as rapid symptomatic treatment (fluid infusion or low-dose vasopressors), antihistamines, NSAIDs, anesthetics, etc., administered intravenously.
Grade 3	Symptoms require aggressive intervention. Hypotension (requiring high-dose vasopressors or two or more vasopressors), hypoxia (oxygen concentration ≥40%), Grade 3 organ toxicity or Grade 4 transaminase elevation.	Continuous monitoring, supportive care (e.g., multiple or single high-dose vasopressors, oxygen therapy), consideration of cytokine antagonists (IL-6 receptor antagonist tocilizumab, TNF-α antibodies such as infliximab, adalimumab, Enbrel (etanercept for injection)), etc.
Grade 4	Life-threatening, requiring mechanical ventilation, or Grade 4 organ toxicity.	Continuous monitoring, high-dose vasopressors, ventilator support, administration of cytokine antagonists (IL-6 receptor antagonist tocilizumab, TNF-α antibodies such as infliximab, adalimumab, Enbrel (etanercept for injection)) and corticosteroids, etc.
Grade 5	Death.	---

In some cases, cytokine storm, tumor lysis syndrome, and hypotension have led to acute kidney injury, requiring temporary dialysis support. If the cytokine storm is too severe, such as worsening dyspnea, pulmonary infiltration, extreme hypoxia requiring oxygen or a

ventilator, abnormal blood pressure despite fluid infusion and moderate vasopressors, and deteriorating clinical status, tocilizumab may be administered. Tocilizumab is an anti-IL-6 receptor antibody drug, administered at a single dose of 4 mg/kg to 8 mg/kg. The use of drugs for cytokine storm treatment, including tocilizumab, should be decided by the attending physician, generally based on hemodynamic abnormalities as the main indicator. Patients with severe cytokine storm experience rapid relief of high fever (within hours) after receiving tocilizumab, while symptoms such as hypotension and hypoxia may persist for several hours to days. Additionally, related biochemical abnormalities (e.g., the accumulation of CD163+ hemophagocytic macrophages in bone marrow and lymph nodes) may appear within 48 hours. Corticosteroids are only administered in life-threatening situations.

VII. Adverse Event Reporting

In clinical studies, timely, accurate, and complete reporting and analysis of safety information are crucial for protecting subjects, investigators, and sponsors, and are mandatory requirements of drug regulatory authorities in various countries. The sponsor has established standard operating procedures (SOPs) to ensure appropriate reporting of safety information; all clinical studies conducted by the sponsor or its affiliated organizations must comply with these procedures.

7.1 Definition

7.1.1 Definition and Classification of Adverse Events

(1) Adverse event

Any untoward medical occurrence in a subject administered an investigational personalized DC vaccine, whether or not considered related to the investigational product. An adverse event can be any unfavorable and unintended sign (including an abnormal laboratory finding), symptom, or disease temporally associated with the use of an investigational (medicinal) product, whether or not considered related to the investigational (medicinal) product [International Council for Harmonisation (ICH) definition]. This includes any event that is new in onset or aggravated in severity or frequency from the

baseline condition, or any abnormal finding identified during a diagnostic procedure, including abnormal laboratory test results.

(2) Serious adverse event

A serious adverse event is defined as any untoward medical occurrence that at any dose of the DC vaccine:

① Results in death;

② Is life-threatening (at the time of the event, the subject was at risk of death. It does not include an event that, had it occurred in a more severe form, might have caused death);

③ Requires inpatient hospitalization or prolongation of existing hospitalization;

④ Results in persistent or significant disability/incapacity;

⑤ Results in a congenital anomaly/birth defect.

Note: In addition to the outcomes listed above, medical and scientific judgment should be exercised in deciding whether expedited reporting is appropriate in other situations, such as important medical events that may not be immediately life-threatening or result in death or hospitalization but may jeopardize the subject or may require intervention to prevent one of the outcomes listed above. Based on the investigator's judgment, any adverse event with significant clinical signs and symptoms that impact the patient is considered a serious adverse event.

(3) Unlisted (Unexpected) Adverse Event

Refers to an adverse event whose nature or severity is inconsistent with the applicable product information (e.g., Investigator's Brochure for an unapproved investigational product or package insert/summary of product characteristics for an approved product).

(4) Related to Drug Use

An adverse event is considered related to the use of the drug if the relationship is judged as definitely related, probably related, or possibly related.

7.1.2 Definition of Causality

(1) Definitely Not Related

The adverse event is not related to the use of the drug.

(2) Probably Not Related

The adverse event is more likely related to another factor, such as concomitant medication or concurrent disease, or the timing of the event suggests it is unlikely to be causally related to the drug use.

(3) Possibly Related

The adverse event might have been caused by the drug. It is uncertain whether the event could be due to other factors such as concomitant medication or concurrent disease. The temporal relationship with drug use is plausible, and a causal relationship cannot be ruled out.

(4) Probably Related

The event is likely to have been caused by the drug use. The timing is suggestive (e.g., confirmed by withdrawal). Other explanations, such as concomitant medication or concurrent disease, are unlikely.

(5) Definitely Related

The adverse event is listed as a possible adverse reaction and cannot be explained by other causes (e.g., concomitant medication and concurrent disease). The timing is consistent with drug use (e.g., confirmed by withdrawal or rechallenge studies).

7.2 Operational Procedures

7.2.1 All Adverse Events

All adverse events occurring from the first dose of the investigational product until 8 weeks after the last dose, except for disease progression, should be reported. Adverse events occurring after 8 weeks that are considered related to the investigational product should also be reported.

Events meeting the definition of a serious adverse event must be reported using the Serious Adverse Event Form, including those spontaneously reported to the investigator within 8 weeks after the subject completes the study (including post-study visits). All events meeting the definition of a serious adverse event must be reported as such, regardless of whether they are specified as assessment items in the study protocol. All adverse events, regardless of severity or causal relationship to the study treatment, must be recorded in medical terminology in the source documents and case report forms (CRFs). When signs and symptoms can be attributed to a common etiology, a diagnosis should be provided

whenever possible (e.g., cough, runny nose, sneezing, and sore throat should be reported as "upper respiratory tract infection"). The investigator must also record the causality of the adverse event to the investigational product, management of the adverse event, and outcome in the source documents and report them as required by the sponsor. The sponsor is responsible for reporting adverse events from the study to regulatory authorities in an appropriate manner. The sponsor is responsible for notifying the investigator of any serious adverse events (SAEs) related to the investigational product that are not listed in the package insert or investigator's brochure. The investigator (or the sponsor if required) is responsible for reporting this information to the independent ethics committee that approved the study protocol, unless otherwise required by the ethics committee. Subjects (or their authorized representatives, if applicable) must be provided with a "Study Card" indicating the name of the investigational product, study code, investigator's name, and a 24-hour emergency contact number.

7.2.2 Serious Adverse Event

All serious adverse events occurring after the subject is enrolled and receives the first dose of the DC vaccine must be reported to the designated sponsor contact within 24 hours of the investigator's awareness. Information regarding the serious adverse event will be completed in the Serious Adverse Event Form and transmitted to the sponsor, with the investigator's signature. The initial report of a serious adverse event may be made by telephone or fax. If reported by telephone, the investigator must complete and submit the Serious Adverse Event Form to the sponsor within one business day after the telephone report. For serious adverse events that have not resolved by the end of the study or upon the subject's early withdrawal, the investigator must follow up until any of the following conditions is met:

① Event resolved;

② Event stabilized;

③ Event returned to baseline level, if a baseline value is available;

④ Event can be attributed to a drug other than the investigational product or to factors unrelated to the study procedures;

⑤ When further information is unlikely to be obtained (the patient or healthcare

provider refuses to provide more information, or there is evidence that the patient has been lost to follow-up despite best efforts).

The cause of death of a subject occurring after enrollment and the first dose of the DC vaccine, whether expected or related to the investigational product, is considered a serious adverse event. Any hospitalization (or prolongation of hospitalization) occurring during the subject's participation in the study must be reported as a serious adverse event, except for hospitalizations that are part of the study protocol/disease-related study procedures (e.g., surgery, scans, endoscopy, laboratory sampling, imaging, pathological sampling, etc.). Complications arising from such procedures leading to hospitalization or prolonged hospitalization must still be reported as serious adverse events. Hospitalizations or prolonged hospitalizations due to technical, practical, or social reasons rather than an adverse event, or scheduled surgeries (planned before the study start) are excluded. However, if the prolongation of hospitalization is due to complications considered at least possibly related to the investigational product, it must be reported as a serious adverse event.

7.2.3 Pregnancy

The investigator must complete the Pregnancy Report Form and report to the sponsor within one business day of becoming aware of a subject's pregnancy. Subjects who become pregnant during the study must be withdrawn from the study. The outcome of the pregnancy and information about the newborn should be followed up.

7.3 Contacting the Sponsor Regarding Safety Issues

Questions or concerns regarding the safety of this study can be directed to the sponsor personnel. The name (and corresponding telephone number) of the sponsor contact will be listed on the contact page, which will be provided as a separate document.

8. Statistical Methods

8.1 Patient Enrollment and Analysis Population Description

Describe the number of enrolled subjects, the number of subjects in the full analysis set, per-protocol set, and safety set, and the number of excluded subjects. Describe the cases of withdrawal.

8.2 General Principles of Statistical Analysis

Unless otherwise specified, all statistical tests will be two-sided, and a p-value less than or equal to 0.05 will be considered statistically significant. Descriptive statistics for quantitative measures will include mean, standard deviation, median, minimum, and maximum. Categorical measures will be described by the number and percentage of subjects in each category. Homogeneity between groups will be compared using t-tests, $\chi 2$ tests, or rank-sum tests.

8.3 Statistical Description and Analysis of Baseline Data

Describe the baseline characteristics of various medical examination indicators before treatment. For categorical data, calculate the number and proportion of subjects in each category; for continuous data, calculate the number of subjects, mean, standard deviation, median, maximum, and minimum.

8.4 Missing Values and Outliers

In principle, there should be no missing values in the CRF, especially for important indicators (e.g., primary efficacy and safety endpoints), which must be clearly filled in. Basic data in the CRF, such as gender, date of birth, date of enrollment, and various observation dates, must not be missing. Negative results observed in the trial, results measured as zero, and those that could not be measured should be indicated with appropriate symbols and not left blank to distinguish them from missing values.

The handling of outliers should be judged from both medical and statistical professional perspectives, with particular emphasis on medical expertise.

8.5 Safety Analysis

The safety analysis set will consist of patients who received at least one dose of the DC vaccine. Describe in detail the types and severity of AEs and SAEs during the project, including time of onset, end time, severity, relationship to the project treatment, measures taken, and outcome; calculate the overall incidence and incidence by system, and analyze related factors stratified by gender, age, stage, initial treatment, retreatment, etc.

8.6 Efficacy Analysis

Descriptive statistics will be used to summarize efficacy endpoints. Kaplan-Meier curves will be used to describe PFS and OS, and median PFS and median OS will be calculated. Calculate RR, DCR, CBR, and their 95% confidence intervals.

- PFS (Progression-Free Survival): Defined as the time from the date of surgery to the date of PD or death from any cause. If it is unclear whether the subject has died or experienced PD, PFS is censored at the date of last follow-up.
- OS (Overall Survival): Defined as the time from the date of surgery to death from any cause. If it is unclear whether the subject has died, OS is censored at the date of last follow-up.
- 1-year survival rate: Defined as the proportion of all patients treated with the DC vaccine who survive for more than 1 year.
- RR (Overall Response Rate): Refers to the proportion of subjects in each treatment group with a best overall response of (CR+PR).
- DCR (Disease Control Rate): Refers to the proportion of subjects in each treatment group with a confirmed best overall response = CR+PR+SD, lasting for at least 4 weeks.
- CBR (Clinical Benefit Rate): Refers to the proportion of subjects in each treatment group with a confirmed best overall response = CR+PR+SD, lasting for at least 6 months.

9. Ethical Principles

This clinical study strictly adheres to the Declaration of Helsinki and follows the principles of China's GCP.

9.1 Ethical Considerations in Study Design

Invasive procedures required by this study, including blood sampling for hematological and clinical biochemical tests and for histopathological/cytopathological examination, are mandated by the RECIST 1.1 efficacy evaluation criteria.

9.2 Ethical Compliance

9.2.1 Investigator Responsibilities

The investigator is responsible for ensuring that the clinical study is conducted in accordance with the protocol, current Good Clinical Practice (GCP), and relevant regulations.

GCP is an international ethical and scientific quality standard for designing, conducting, recording, and reporting research involving human subjects. Compliance with this standard ensures that the rights, safety, and well-being of trial subjects are protected in accordance with the principles of the Declaration of Helsinki and that the clinical trial data are credible.

9.2.2 Independent Ethics Committee/Institutional Review Board (IEC/IRB)

Prior to study initiation, the investigator (or sponsor, if required) shall provide the IEC/IRB with copies of the following documents:

① Final study protocol and amendments (if applicable);

② Sponsor-approved informed consent form (and other written information provided to subjects);

③ Investigator’s Brochure (or equivalent information) and updates;

④ Subject recruitment materials;

⑤ Information on compensation for study-related injury or payments to subjects for participation in the study, if applicable;

⑥ Investigator’s curriculum vitae or equivalent information (unless the IEC/IRB has documentation stating that this information is not required);

⑦ Information on study funding, sponsor name, institution, etc., that may involve conflicts of interest with the subject;

⑧ Other documents required by the IEC/IRB to fulfill its obligations.

Clinical studies may only be implemented after the IEC/IRB has fully approved the final study protocol, protocol amendments (if any), informed consent form, implemented recruitment materials, and subject compensation plan, and the sponsor has received a copy of the approval document. The approval document must bear the approval date and identify the approved documents. At appropriate times during the study, the investigator will also

submit the following documents to the IEC/IRB for review and approval:

① Protocol amendments;

② Revised informed consent forms and any other written materials provided to subjects;

③ If applicable, new or revised subject recruitment materials approved by the sponsor;

④ If applicable, revised information on compensation for study-related injury and payments to subjects for participation in the study;

⑤ Investigator's Brochure updates or new editions;

⑥ Study progress summaries (at least annually or as required by the IEC/IRB);

⑦ Reports of serious, unlisted, study drug-related adverse events;

⑧ New information that may adversely affect subject safety or the safety of the study;

⑨ Protocol deviations or changes made to eliminate immediate danger to subjects;

⑩ Reports of subject deaths under the investigator's care;

11 Notify the IEC/IRB if there is a new responsible investigator at the study site;

12 Any other documents required by the IEC/IRB.

If protocol amendments increase subject risk, the amendments must be submitted to the IEC/IRB for review and approval along with the revised informed consent form before these changes are implemented. At least annually, the IEC/IRB is required to review and re-approve this clinical study. The requirement for re-review and re-approval must be documented in writing. At the end of the study, the investigator (or sponsor, if required) will notify the IEC/IRB that the study has ended.

9.2.3 Informed Consent

According to local regulations, each subject must provide written consent after being fully explained the purpose and content of the study. Before performing any study-related procedures, the subject must sign the informed consent form. The informed consent form and consent form used must be approved by the sponsor and approved by the IEC/IRB. The informed consent form must comply with the principles of the Declaration of Helsinki, current ICH and GCP guidelines, current regulations, and sponsor policies.

Before a subject enters the study, the investigator or authorized research staff must

explain the purpose, methods, expected benefits and potential risks of the study, and any possible discomfort that may result. Subjects are informed that their participation is voluntary and that they may withdraw at any time. Participation in the study will not affect the treatment of their disease. Subjects are informed that if they refuse to participate in the study, they can still choose other treatment methods, and such refusal will not affect the investigator's future treatment of them. Finally, subjects are also informed that their identity records will be kept for long-term follow-up, and that these records may be viewed by drug regulatory authorities or authorized sponsor personnel under current laws and regulations, and such viewing will not compromise the confidentiality of subject data. Once the subject signs the informed consent form, they authorize the above actions.

Subjects should have sufficient time to read the informed consent form and have the opportunity to ask questions. After receiving explanations and before entering the study, this informed process is documented by the subject signing and dating the informed consent form. After informed consent, the subject will receive a copy of the informed consent form.

If the subject (or legal representative) cannot read or write, an impartial witness should participate in the entire informed consent process (including reading and explaining all written information) and personally sign and date after the subject (or legal representative) gives oral consent.

9.2.4 Privacy of Personal Data

The collection and processing of personal data of subjects participating in this study is limited to research on the efficacy, safety, quality, and use of the drug. Sufficient measures will be taken during the collection and processing of this data to ensure data confidentiality and compliance with current laws and regulations on privacy protection.

The sponsor ensures that personal data:

① Is processed fairly and lawfully;

② Is collected for the legitimate purposes specified in the protocol and will not be processed in other ways inconsistent with these purposes;

③ Is adequate, relevant, and appropriate for the stated purposes;

④ Is accurate and, where necessary, kept up to date;

Before collecting personal data, the investigator will obtain the subject's consent for the processing of personal data. This consent also includes the transfer of data to other institutions or countries.

Subjects have the right to request their personal data from the investigator and to request correction of incorrect or incomplete personal data. Reasonable actions should be taken after considering the nature of the request, study conditions, and current laws and regulations.

Appropriate technical methods and administrative measures should be taken to protect personal data from disclosure or unauthorized access, accidental or unlawful destruction, and accidental loss or tampering. Sponsor personnel who have access to personal data due to job responsibilities will keep the identity of study subjects confidential.

10. Document Management

10.1 Study Protocol Amendments

Investigators shall not modify this study protocol without a formal amendment. All study protocol amendments must be issued by the investigator and signed and dated by the investigator. Study protocol amendments shall not be implemented until approved by the IEC/IRB or unless any objections from relevant regulatory authorities are raised, except to avoid immediate risk to subjects, in which case the amendment must be immediately submitted to the IEC/IRB and the corresponding regulatory authorities. If the change only involves logistical or administrative aspects of the study, only notification to the IRB is required.

During the study, if protocol deviations are unavoidable, except in emergencies, the institutional management representative must be contacted before implementing any protocol deviations. In any case, the specific situation must be discussed as soon as possible, and agreed measures must be taken and reported to the ethics committee. Data recorded in the eCRF and source documents should reflect any protocol deviations that occur, and the source documents should also describe the deviations.

10.2 Registration Documents

10.2.1 Registration Approval/Notification

This study protocol and its amendments must be submitted to the relevant departments of the institution. Clinical studies may only begin after all requirements of local regulatory authorities are met.

10.2.2 Pre-study Required Documents

The following documents must be prepared before the study begins:

Study protocol and amendments (if any) signed and dated by the principal investigator;

A written copy of the approval for the study protocol, amendments, informed consent form, and recruitment materials, signed and dated (or stamped, in accordance with local regulations) by the IEC/IRB. This approval document must clearly indicate the study title and code, and must be signed by the study director;

Name and address of the IEC/IRB, including the current list of IEC/IRB members and their functions. Documentation is required stating that its organization and operation comply with GCP and current laws and regulations. If the IEC/IRB provides explanatory documentation, a summary statement may replace this list. If the investigator is a member of the IEC/IRB, written documentation must state that this person did not participate in the discussion or voting/decision-making for this study;

Institutional management approval or notification (if applicable);

Signed and dated statement by the investigator (if applicable);

Investigator qualification documents (e.g., curriculum vitae);

Investigator financial disclosure form completed by the principal investigator (if applicable);

Signed and dated clinical study agreement, including financial agreement;

Any other documents required by local regulations.

Name and address of the local laboratory performing tests for this study, and a dated copy of the current laboratory normal range values for these tests (if applicable);

Documentation demonstrating the capability and reliability of the local laboratory (e.g., laboratory certification/license) (if applicable).

10.3 Subject Identification Code List and Subject Screening/Enrollment Log

The investigator agrees to complete the subject identification code list to facilitate the identification of subjects during and after the study. The sponsor's monitor will verify the completeness of this document.

The subject identification code list is a confidential document, and the investigator will keep this document in the study site file. To ensure the confidentiality of subject identity, this log shall not be copied. All study-related reports and correspondence shall use the subject's initials and number to identify the subject.

The investigator must also complete the subject screening/enrollment log, which records all subjects who have been seen by the physician and for whom the physician has determined eligibility.

10.4 Source Documents

Source documents must contain at least the following information to verify the data collected in the eCRF: subject identification information, eligibility and study identification information, date of study discussion and signed informed consent, visit dates, results of safety and efficacy evaluations required by the protocol; records of all adverse events and follow-up of adverse events; concomitant medications; record of drug administration; drug administration information; study completion date and reason for premature discontinuation of study drug or withdrawal from the study (if applicable).

In addition, the personnel recording the source documents should be identified.

The type and level of detail of the source data for study subjects should at least be consistent with the common records at the study site based on standard medical care. The specific details required as source data for the study will be reviewed by the investigator before the study and described in the monitoring guidelines (or other equivalent document).

Subject assessments designated by the investigator will be recorded directly in the electronic medical record and considered source data.

Minimum requirements for source documents regarding Section 4.2 "Inclusion Criteria" and Section 4.3 "Exclusion Criteria" (specifying whether a documented medical history is required) are as follows:

For inclusion and exclusion criteria that do not require a documented medical history, verification shall be made at least through meeting the subject or other assessments required by the study protocol (e.g., physical examination, pathologic findings, laboratory evaluations) and recorded in the source documents.

10.5 Completion of Case Report Forms

Case report forms in electronic format are provided for each subject.

This study will use an electronic data capture (EDC) system. Study staff will transcribe study data from source documents into the eCRF. The electronic file will be considered the eCRF.

Medical history registration forms may be used to obtain certain data to facilitate completion of the eCRF. Any such form will become part of the subject's source documents. All study-related data must be recorded in the eCRF. Data must be entered into the eCRF in Chinese. Site personnel must complete the eCRF as soon as possible after the subject's visit, and these forms should be available for review during the next scheduled monitoring visit.

Every effort should be made to ensure that all subjective evaluations to be recorded in the case report form (such as pain scale information or other questionnaires) are completed by the same personnel who performed the initial baseline assessment. The investigator must ensure that all data entered into the eCRF are accurate and credible.

All data entry, corrections, and changes on the eCRF must be completed by the investigator or other authorized research personnel. If necessary, query messages will be generated in the Electronic Data Capture (EDC) system. The investigator or authorized research personnel must make corrections to the eCRF (if applicable) and resolve all queries.

If corrections to the eCRF are required after initial entry, they can be made in the following three ways:

The site staff can make modifications in the Electronic Data Capture (EDC) system either spontaneously or in response to automated queries (generated by the EDC system).

The central management can raise queries that require resolution by the investigator.

10.6 Data Quality Assurance/Quality Control

Steps required to ensure data accuracy and reliability include: selection of investigators and study sites, review of the protocol by the investigator and relevant

personnel before the study begins, and periodic monitoring visits by the regulatory authority. Instructions for the purchase of investigational products (such as copies of drug purchase receipts) will be provided.

Guidelines for completing the eCRF will be provided before the study begins and reviewed with the investigators.

The regulatory authority will conduct periodic monitoring visits at the study site and, after receiving transmitted study data, check the accuracy and completeness of the eCRF; any discrepancies will be resolved with the investigator. After data are uploaded to the clinical research database, their accuracy and consistency with the source data will be verified.

10.7 Record Retention

In accordance with ICH/GCP guidelines, the investigator/institution will retain all case report forms and all original documents supporting the data collected from each subject, all study documents specified in Section 8 of ICH/GCP (Essential Documents for the Conduct of a Clinical Trial), and all study documents required by relevant regulatory requirements. The investigator/institution must take appropriate measures to prevent accidental or premature destruction of these documents.

Essential documents must be retained for at least 2 years after the final marketing approval of the product in the ICH region, and until at least 2 years have passed since the last ICH region has no pending or contemplated marketing applications, or the clinical development of the investigational product has been formally discontinued. If required by relevant regulatory requirements or agreements with the investigator, these documents will be retained for a longer period. The regulatory authority is responsible for notifying the investigator/institution when these documents no longer need to be retained.

If the responsible investigator retires, relocates, or is otherwise unable to continue retaining the study records, the custody of the study records will be transferred to a person who accepts this responsibility. The regulatory authority must be notified in writing of the name and address of the new responsible person. Under no circumstances may the investigator transfer or dispose of any study documents without written permission from the regulatory authority. If relevant legal authorities require review of study-related documents,

the investigator must allow access to these reports.

10.8 Study Completion/Termination

10.8.1 Study Completion

The study is considered completed when the last subject participating in the study has completed the final study evaluation. After the study site completes the evaluation of the last subject, the final data will be submitted to the investigator (or designated personnel) for review within the timeframe specified in the clinical research agreement.

10.8.2 Study Termination

The investigator reserves the right to close the study site or terminate the study at any time for any reason after careful consideration. The study site will be closed upon study completion. The study site is considered closed when all required documents and study supplies have been collected.

If there is a valid reason and sufficient notice has been given prior to the anticipated site closure, the investigator may initiate the site closure procedure at any time.

Reasons for the investigator to close the study site early may include, but are not limited to, the following:

① Requirements from the IEC/IRB or local health authorities, or requirements from investigator procedures or Good Clinical Practice (GCP) guidelines;

② Insufficient number of subjects recruited by the investigator;

③ Discontinuation of further drug development.

10.9 Site Audit

During or after the study, representatives from the investigator's clinical quality assurance department may visit the study site at any time to conduct audits in accordance with relevant regulations and institutional policies. During the audit, the representative may review all study records, including source documents, for the purpose of checking and comparing the eCRF. However, the privacy of the subjects must be respected. During routine audit visits conducted by the regulatory authority or its designee, the investigator and research staff must be present and available for questioning.

10.10 Clinical Trial Registration and Results Disclosure

The investigator must register and/or disclose the results of the clinical trial in

accordance with legal requirements.

11. Anticipated Progress

Anticipated study start date: May 2021

Anticipated study end date: December 2023

12. References

[1] Ostrom QT, Gittleman H, Truitt G, et al. CBTRUS Statistical Report: Primary Brain and Other Central Nervous System Tumors Diagnosed in the United States in 2011-2015. Neuro Oncol. 2018;20(suppl_4):iv1-iv86.

[2] Louis DN, Perry A, Reifenberger G, et al. The 2016 World Health Organization Classification of Tumors of the Central Nervous System: a summary. Acta Neuropathol. 2016;131(6):803-20.

[3] Ohgaki H, Kleihues P. Genetic pathways to primary and secondary glioblastoma. Am J Pathol. 2007;170(5):1445-53.

[4] Aldape K, Zadeh G, Mansouri S, et al. Glioblastoma: pathology, molecular mechanisms and markers. Acta Neuropathol. 2015;129(6):829-48.

[5] Ohgaki H, Kleihues P. The definition of primary and secondary glioblastoma. Clin Cancer Res. 2013;19(4):764-72.

[6] Chen WQ, Li H, Sun KX, et al. [Report of Cancer Incidence and Mortality in China, 2014]. Zhonghua zhong liu za zhi [Chinese journal of oncology]. 2018;40(1):5-13.

[7] Cuddapah VA, Robel S, Watkins S, et al. A neurocentric perspective on glioma invasion. Nature reviews Neuroscience. 2014;15(7):455-65.

[8] Chinese Glioma Cooperative Group (CGCG). Chinese Guidelines for Diagnosis and Treatment of Central Nervous System Glioma (2015). National Medical Journal of China. 2016;7.

[9] Stupp R, Mason WP, van den Bent MJ, et al. Radiotherapy plus concomitant and adjuvant temozolomide for glioblastoma. The New England journal of medicine. 2005;352(10):987-96.

[10] Laperriere N, Zuraw L, Cairncross G, et al. Radiotherapy for newly diagnosed malignant glioma in adults: a systematic review. Radiother Oncol. 2002;64(3):259-73.

[11] Stupp R, Hegi ME, Mason WP, et al. Effects of radiotherapy with concomitant and adjuvant temozolomide versus radiotherapy alone on survival in glioblastoma in a randomised phase III study: 5-year analysis of the EORTC-NCIC trial. Lancet Oncol. 2009;10(5):459-66.

[12] Westphal M, Ram Z, Riddle V, et al. Gliadel wafer in initial surgery for malignant glioma: long-term follow-up of a multicenter controlled trial. Acta Neurochir (Wien). 2006;148(3):269-75

[13] Louveau A, Harris TH, Kipnis J. Revisiting the Mechanisms of CNS Immune Privilege. Trends Immunol. 2015;36(10):569-77.

[14] Louveau A, Smirnov I, Keyes TJ, et al. Structural and functional features of central nervous system lymphatic vessels. Nature. 2015;523(7560):337-41.

[15] Engelhardt B, Vajkoczy P, Weller RO. The movers and shapers in immune privilege of the CNS. Nature immunology. 2017;18(2):123-31.

[16] Mellman I, Coukos G, Dranoff G. Cancer immunotherapy comes of age. Nature. 2011;480(7378):480-9.

[17] Couzin-Frankel J. Breakthrough of the year 2013. Cancer immunotherapy. Science. 2013;342(6165):1432-3.

[18] Prasad V. Immunotherapy: Tisagenlecleucel - the first approved CAR-T-cell therapy: implications for payers and policy makers. Nat Rev Clin Oncol. 2018;15(1):11-2.

[19] O'Rourke DM, Nasrallah MP, Desai A, et al. A single dose of peripherally infused EGFRvIII-directed CAR T cells mediates antigen loss and induces adaptive resistance in patients with recurrent glioblastoma. Sci Transl Med. 2017;9(399).

[20] Topalian SL, Drake CG, Pardoll DM. Immune checkpoint blockade: a common denominator approach

to cancer therapy. Cancer Cell. 2015;27(4):450-61.
[21] Champiat S, Ferte C, Lebel-Binay S, et al. Exomics and immunogenics: Bridging mutational load and immune checkpoints efficacy. Oncoimmunology. 2014;3(1):e27817.
[22] Hodges TR, Ott M, Xiu J, et al. Mutational burden, immune checkpoint expression, and mismatch repair in glioma: implications for immune checkpoint immunotherapy. Neuro Oncol. 2017;19(8):1047-57.
[23] Melief CJ, van Hall T, Arens R, et al. Therapeutic cancer vaccines. J Clin Invest. 2015;125(9):3401-12.
[24] Palucka K, Banchereau J. Dendritic-cell-based therapeutic cancer vaccines. Immunity. 2013;39(1):38-48.
[25] Cheever MA, Higano CS. PROVENGE (Sipuleucel-T) in prostate cancer: the first FDA-approved therapeutic cancer vaccine. Clin Cancer Res. 2011;17(11):3520-6.
[26] Tanyi JL, Bobisse S, Ophir E, et al. Personalized cancer vaccine effectively mobilizes antitumor T cell immunity in ovarian cancer. Sci Transl Med. 2018;10(436).
[27] Rob L, Mallmann P, Knapp P, et al. Dendritic cell vaccine (DCVAC) with chemotherapy (ct) in patients (pts) with epithelial ovarian carcinoma (EOC) after primary debulking surgery (PDS): Interim analysis of a phase 2, open-label, randomized, multicenter trial. 2018;36(15_suppl):5509-.
[28] Sakai K, Shimodaira S, Maejima S, et al. Dendritic cell-based immunotherapy targeting Wilms' tumor 1 in patients with recurrent malignant glioma. J Neurosurg. 2015;123(4):989-97.
[29] Hilf N, Kuttruff-Coqui S, Frenzel K, et al. Actively personalized vaccination trial for newly diagnosed glioblastoma. Nature. 2019;565(7738):240-5.
[30] Keskin DB, Anandappa AJ, Sun J, et al. Neoantigen vaccine generates intratumoral T cell responses in phase Ib glioblastoma trial. Nature. 2019;565(7738):234-9.
[31] Yao Y, Luo F, Tang C, et al. Molecular subgroups and B7-H4 expression levels predict responses to dendritic cell vaccines in glioblastoma: an exploratory randomized phase II clinical trial. Cancer Immunol Immunother. 2018;67(11):1777-88.
[32] Migliorini D, Dutoit V, Allard M, et al. Phase I/II trial testing safety and immunogenicity of the multipeptide IMA950/poly-ICLC vaccine in newly diagnosed adult malignant astrocytoma patients. Neuro Oncol. 2019.
[33] Weller M, Roth P, Preusser M, et al. Vaccine-based immunotherapeutic approaches to gliomas and beyond. Nat Rev Neurol. 2017;13(6):363-74.
[34] Patel AP, Tirosh I, Trombetta JJ, et al. Single-cell RNA-seq highlights intratumoral heterogeneity in primary glioblastoma. Science. 2014;344(6190):1396-401.
[35] Ott P A, Hu Z, Keskin D B, et al. An immunogenic personal neoantigen vaccine for patients with melanoma[J]. Nature, 2017, 547(7662): 217.
[36] Sahin U, Derhovanessian E, Miller M, et al. Personalized RNA mutanome vaccines mobilize poly-specific therapeutic immunity against cancer[J]. Nature, 2017, 547(7662): 222.
[37] Jacob S. Y, Fara D, Ramin A. M. et al, Immunotherapy for High-Grade Gliomas: A Clinical Update and Practical Considerations for Neurosurgeons. WORLD NEUROSURGERY 2019, 124: 397-409

13. Appendices

Appendix 1 Karnofsky Performance Status (KPS) Scale

Score	Performance Status
100	Normal, no complaints, no evidence of disease
90	Able to carry on normal activity, minor signs or symptoms of disease
80	Normal activity with effort, some signs or symptoms of disease
70	Cares for self, unable to carry on normal activity or do active work
60	Requires occasional assistance but is able to care for most personal needs
50	Requires considerable assistance and frequent medical care
40	Disabled, requires special care and assistance
30	Severely disabled, hospitalization is indicated although death not imminent
20	Very sick, hospitalization necessary, active supportive treatment required
10	Moribund, fatal processes progressing rapidly
0	Dead

Appendix 2 Eastern Cooperative Oncology Group (ECOG) Performance Status Scale (ZPS, 5-point scale)

Grade	Performance Status
0	Fully active, able to carry on all pre-disease performance without restriction.
1	Restricted in physically strenuous activity but ambulatory and able to carry out work of a light or sedentary nature, e.g., light house work, office work.
2	Ambulatory and capable of all self-care but unable to carry out any work activities; up and about more than 50% of waking hours.
3	Capable of only limited self-care, confined to bed or chair more than 50% of waking hours.
4	Completely disabled, cannot carry on any self-care; totally confined to bed or chair.
5	Dead.

Appendix 3 NCI-CTCAE V5.0 Grading Criteria for Common Acute and Subacute Toxicities

Toxicity	Grade				
	1	2	3	4	5
Hemoglobin	100 g/L - lower limit of normal	100 - 80 g/L	80 - 65 g/L; Requires transfusion	Life-threatening; Requires urgent intervention	Death
Leukopenia	<LLN - $3.0×10^9$/L	(2.0-3.0) $×10^9$/L	(1.0-2.0) $×10^9$/L	<$1.0×10^9$/L	---
Neutropenia	(1-1.5) $×10^9$/L	(1.5-1.0) $×10^9$/L	(1.0 -0.5) $×10^9$/L	<0.5× 10^9/L	---
Lymphocytopenia	<$0.8×10^9$/L	(0.5-0.8) $×10^9$/L	(0.2-0.5) $×10^9$/L	<0.2 $×10^9$/L	---
Platelet count Decreased	<LLN - $75.0×10^9$/L	(50.0-75.0) $×10^9$/L	(25.0-50.0) $×10^9$/L	<$25.0×10^9$/L	---
Prolonged prothrombin time	(1-1.5) × ULN	(1.5-2.5) × ULN	>2.5× ULN; bleeding	---	---
Hyperbilirubinemia	(1-1.5) × ULN	(1-3.0) × ULN	(3.0-10.0) × ULN	>10.0× ULN	---
Alanine aminotransferase increased (ALT/SGPT)	(1.0-3.0)× ULN	Asymptomatic: (3.0-5.0)× ULN; >3× ULN with worsening of symptoms: fatigue, nausea, vomiting, right upper quadrant pain or tenderness, fever, rash, eosinophilia.	(5.0-20.0)× ULN; >5.0× ULN for more than 2 weeks	20.0× ULN	---
Aspartate aminotransferase increased (AST/SGOT)	(1-3.0)× ULN	Asymptomatic: (3.0-5.0)× ULN; >3.0× ULN with worsening of symptoms: fatigue, nausea, vomiting, right upper quadrant pain or	(5.0-20.0)× ULN; >5.0× ULN for more than 2 weeks	>20.0× ULN	---

**Toxicity**	**Grade**				
	**1**	**2**	**3**	**4**	**5**
		tenderness, fever, rash, eosinophilia.			
Alkaline phosphatase increased (AKP)	ULN - 2.5× ULN	(2.5-5.0)× ULN	(5.0-20.0)× ULN	>20.0× ULN	---
Creatinine	Creatinine >0. 3 mg/dL; or 1.5-2.0× baseline	Creatinine 2.0-3.0× baseline	Creatinine >3.0× baseline or >4.0 mg/dL; requires hospitalization	Life-threatening; requires dialysis	Deat h
Proteinuria	Proteinuria 1+, 24-hour urine protein <1.0 g	Adults: Proteinuria 2+, 24-hour urine protein 1.0-3.4 g; Children: Urine protein/creatinin e ratio 0.5-1.9	Adults: 24-hour urine protein >3.5 g; Children: Urine protein/creatinin e ratio >1.9	---	---
Hematuria	Asymptomatic, clinical or diagnostic observations only; intervention not indicated	Mild symptoms, requiring catheterization or bladder irrigation; affecting instrumental activities of daily living	Gross hematuria, requiring transfusion, intravenous medication, or hospitalization; requiring elective endoscopy, radiotherapy, or surgical intervention; affecting personal activities of daily living	Life-threatening; requiring urgent radiological or surgical intervention	Deat h

Appendix 4 NCI CTCAE 5.0 Adverse Event Grading

Toxicity	Grade				
	1	2	3	4	5
Cytokine Release Syndrome	Mild reactions, such as fever, nausea, vomiting, sweating, rash, fatigue, exhaustion, myalgia, etc.	Symptoms require moderate intervention. Hypotension (responsive to fluid replacement or a single low-dose vasopressor), hypoxia (oxygen concentration <40%), Grade 2 organ toxicity.	Symptoms require aggressive intervention. Hypotension (requiring high-dose vasopressors or two or more vasopressors), hypoxia (oxygen concentration ≥ 40%), Grade 3 organ toxicity or Grade 4 transaminase elevation.	Life-threatening, or requiring mechanical ventilation, or Grade 4 organ toxicity	Death
Fever	38.0-39.0 °C	> 39-4.0 °C	> 40.0 °C，≤24 hours	> 40.0 °C，> 24 hours	Death
Hypotension	Asymptomatic; No intervention required	Non-urgent medical intervention	Medical intervention or Requiring hospitalization	Life-threatening, Requiring urgent intervention	Death
Infection	None	Localized; local treatment (e.g., topical antibiotics, antifungals or antiviral therapy)	Requiring intravenous antibacterial, antifungal, or antiviral therapy; Requiring radiological or surgical intervention	Life-threatening; Requiring urgent intervention	Death
Nausea	Decreased appetite without change in eating habits	Reduced oral intake without significant weight loss, dehydration, or malnutrition	Insufficient oral energy and fluid intake; requiring nasogastric tube feeding, total parenteral nutrition, or hospitalization.	---	---

**Toxicity**	**Grade**				
	**1**	**2**	**3**	**4**	**5**
Vomiting	1-2 episodes in 24 hours (separated by 5 minutes)	3-5 episodes in 24 hours (separated by 5 minutes)	≥ 6 episodes in 24 hours (separated by 5 minutes), requiring nasogastric tube feeding, total parenteral nutrition, or hospitalization	Life-threatening: requiring urgent intervention	Death
Fatigue	Fatigue relieved by rest	Fatigue not relieved by rest, affecting instrumental activities of daily living	Fatigue not relieved by rest, affecting personal activities of daily living	---	---
Rash	Transient flushing or rash; drug fever <38°C, no treatment required	Requiring intervention or infusion therapy; prompt symptomatic treatment (e.g., antihistamines, NSAIDs, narcotics); prophylactic medication for ≤24 hours	Prolonged treatment (e.g., not responsive to prompt symptomatic and/or infusion therapy); recurrence after resolution; sequelae (e.g., renal failure, pulmonary infiltrates) requiring hospitalization.	Life-threatening; requiring urgent intervention	Death
Headache	Mild headache	Moderate headache; affecting instrumental activities of daily living	Severe headache; affecting personal activities of daily living	---	---
Muscle/joint pain	Mild pain	Moderate pain; affecting instrumental activities of daily living	Severe pain; affecting personal activities of daily living	---	---
Abdominal pain	Mild pain	Moderate pain; affecting instrumental activities of daily living	Severe pain; affecting personal activities of daily living	---	---

**Toxicity**	**Grade**				
	**1**	**2**	**3**	**4**	**5**
Breathing Difficulty	Shortness of breath during moderate activity	Shortness of breath during light activity; affecting instrumental activities of daily living	Shortness of breath at rest; affecting personal activities of daily living	Life-threatening; requiring urgent intervention	Death
Heart Tachycardia	Asymptomatic, no treatment required	Symptomatic; non-urgent medical intervention	Requiring urgent intervention	---	---
Peripheral motor neuropathy	Asymptomatic, clinical or diagnostic observations only; no intervention indicated	Moderate symptoms; Affecting instrumental activities of daily living	Severe symptoms; Affecting personal activities of daily living; requiring assistive device	Life-threatening, Requiring urgent intervention	Death
Peripheral sensory neuropathy	Asymptomatic, loss of deep tendon reflexes or paresthesia	Moderate symptoms; Affecting instrumental activities of daily living	Severe symptoms; Affecting personal activities of daily living	Life-threatening, Requiring urgent intervention	Death
Pain	Mild pain	Moderate pain; Affecting instrumental activities of daily living	Severe pain; Affecting personal activities of daily living	---	---

Appendix 5 FACT-G Quality of Life Score

Below is a list of statements that other people with your illness have said are important. Please select the number that best describes your situation in the past 7 days.

	Daily activities	Not at all	A little bit	Somew hat	Quite a bit	Very much
GP1	I lack energy	0	1	2	3	4
GP2	I have nausea	0	1	2	3	4
GP3	I am unable to perform my usual household tasks	0	1	2	3	4
GP4	I have pain	0	1	2	3	4
GP5	I am bothered by side effects of treatment	0	1	2	3	4
GP6	I feel weak all over	0	1	2	3	4
GP7	I have to stay in bed	0	1	2	3	4

Please circle the number that best applies to you for each statement based on the past 7 days.

	Social/Family life	Not at all	A little bit	Somew hat	Quite a bit	Very much
GS1	I feel close to my friends	0	1	2	3	4
GS2	I get emotional support from my family	0	1	2	3	4
GS3	I get support from my friends	0	1	2	3	4
GS4	My family has accepted my illness	0	1	2	3	4
GS5	I am satisfied with family communication about my illness	0	1	2	3	4
GS6	I feel close to my partner (or the person who is my main support)	0	1	2	3	4
Q1	Regardless of your current level of sexual activity, please answer the following question. If you prefer not to answer it, please mark this box □ and go to the next section.					
GS7	I am satisfied with my sex life	0	1	2	3	4

Please circle the number that best applies to you for each statement based on the past 7 days.

	Emotional	Not at all	A little bit	Somewhat	Quite a bit	Very much
GE1	I feel sad	0	1	2	3	4
GE2	I am proud of how I'm coping with my illness	0	1	2	3	4
GE3	I feel discouraged by my illness	0	1	2	3	4
GE4	I feel nervous	0	1	2	3	4
GE5	I worry about dying	0	1	2	3	4
GE6	I worry that my condition will get worse	0	1	2	3	4

Please circle the number that best applies to you for each statement based on the past 7 days.

	Functional	Not at all	A little bit	Somewhat	Quite a bit	Very much
GF1	I am able to work (including work at home)	0	1	2	3	4
GF2	I am working to my satisfaction (including work at home)	0	1	2	3	4
GF3	I am enjoying life right now	0	1	2	3	4
GF4	I am able to accept my illness	0	1	2	3	4
GF5	I am sleeping well	0	1	2	3	4
GF6	I am enjoying my usual leisure activities	0	1	2	3	4
GF7	I am concerned about my quality of life right now	0	1	2	3	4

Appendix 6 EORTC QLQ-C30 Quality of Life Questionnaire (Version 3.0)

	Not at all	A little	Moderately	Very much
1. Do you have any trouble doing strenuous activities, like carrying a heavy shopping bag or a suitcase?	1	2	3	4
2. Do you have any trouble taking a long walk?	1	2	3	4
3. Do you have any trouble taking a short walk outside?	1	2	3	4
4. Do you need to stay in bed or a chair during the day?	1	2	3	4
5. Do you need help with eating, dressing, washing yourself or using the toilet?	1	2	3	4

During the past week	Not at all	A little	Moderately	Very much
6. Were you limited in doing either your work or other daily activities?	1	2	3	4
7. Were you limited in pursuing your hobbies or other leisure time activities?	1	2	3	4
8.Are you short of breath?	1	2	3	4
9.Are you in pain?	1	2	3	4
10.Do you need to rest?	1	2	3	4
11.Do you have trouble sleeping?	1	2	3	4
12.Do you feel weak?	1	2	3	4
13.Do you have a poor appetite?	1	2	3	4
14.Do you feel nauseous?	1	2	3	4
15.Have you vomited?	1	2	3	4
16.Are you constipated?	1	2	3	4
17.Do you have diarrhea?	1	2	3	4
18.Do you feel tired?	1	2	3	4
19.Does pain interfere with your daily activities?	1	2	3	4

20.Do you have difficulty concentrating on things, like reading the newspaper or watching television?	1	2	3	4
21.Do you feel nervous?	1	2	3	4
22.Do you feel worried?	1	2	3	4
23.Do you feel irritable?	1	2	3	4
24.Do you feel depressed?	1	2	3	4
25.Do you have difficulty remembering things?	1	2	3	4
26.Has your physical condition or medical treatment affected your family life?	1	2	3	4
27.Has your physical condition or medical treatment affected your social activities?	1	2	3	4
28.Has your physical condition or medical treatment caused you financial difficulties?	1	2	3	4

Please circle the number (1-7) that best applies to you for the following questions

29.How would you rate your overall health in the past week?						
1	2	3	4	5	6	7
Very poor Very good						

30. How would you rate your overall quality of life in the past week?						
1	2	3	4	5	6	7
Very poor Very good						

Appendix 7 Study Flow Chart

Time (Time Window) / Study Phase / Item	Before the end of chemoradiotherapy (Before Day 0)	Day 7 (-7 ~ +4 days)	Day 21 (-7~0 days)	Day 28 (-7~0 days)	Day 35 (-7~0 days)	Day 56 (-7~0 days)	Day 84 (-7~0 days)	Day 112/140/168 (-7~0 days)	Intensification Therapy after TMZ Adjuvant Chemotherapy	Every 3 months
	Specimen Collection and Neoantigen Identification	Screening and Enrollment	Vaccination 1	Vaccination 2	Vaccination 3	Vaccination 4	Vaccination 5	Vaccination 6-818	Vaccination Courses 2-319	Follow-up Period
Informed Consent Form	√									
Demographics	√									
Physical Examination	√	√	√	√	√	√	√	√	√	√
Vital Signs1		√	√	√	√	√	√	√	√	√
Electrocardiogram		√	√	√	√	√	√			
Echocardiogram		√								
Medical History and Treatme	√	√								

**nt History**										
**Tumor Pathology Testing**	√									
**Tumor Imaging Evaluation2**		√					√		√	√
**Blood Sample Collection3**		√			A		A	A	B	
**Cerebrospinal Fluid Collection4**							√			
**Blood Routine5**		√	√	√	√	√	√			
**Immune Cell Phenotyping6**			√	√	√	√	√			
**Blood Biochemistry7**		√	√	√	√	√	√			
**Coagulation Routine8**		√	√	√	√	√	√			
**Urine Routine9**		√	√	√	√	√	√			
**Pregnancy Test10**		√								
**Serum Virus Testing11**		√								
**Serum Ig Levels12**			√	√	√	√	√			
**Cytokine Testing13**			√	√	√	√	√			

**Physical Fitness Assessment14**		√	√	√	√	√	√	√	√	√
**Quality of Life Assessment15**		√	√	√	√	√	√	√	√	√
**Adverse Event Recording16**			√	√	√	√	√	√	√	√
**Concomitant Medication Recording17**		√	√	√	√	√	√	√	√	√

Note:

1. Vital Signs: Key vital signs (temperature, respiration, pulse, oxygen saturation, and blood pressure) should be recorded before and after vaccination. Record every 15 minutes after vaccination for at least 1 hour, or until the condition is stable. Observe in the hospital for 24 hours after vaccination. If there are no significant side effects, discharge may be decided by the physician. If side effects occur, discharge should be decided by the physician 48 hours after the side effects subside.
2. Tumor Imaging Evaluation: Clinical efficacy is assessed using iRANO criteria based on cranial MR (plain scan + contrast + FLAIR + DWI) and the patient's clinical response. Evaluations are conducted at enrollment and after Vaccination 5; thereafter, reassess every 3 months ±7 days, using the date of the clinical efficacy assessment after Vaccination 5 as the reference.
3. Blood Sample Collection: On Day 7 (-7 ~ +4 days), perform apheresis to collect PBMCs for DC vaccine preparation. Use these PBMCs and collect 15 mL of anticoagulated blood one week after the 3rd, 5th, and final (if more than 5) vaccinations (A) for immune response analysis (immune repertoire sequencing, whole exome sequencing, ELISPOT analysis, cytokine analysis, immune cell phenotyping). If the patient meets the criteria for subsequent intensification therapy, collect 15 mL of anticoagulated blood once during and once after each treatment course (B) at appropriate times for the above immune response analyses.
4. Cerebrospinal Fluid Collection: Collect 3 mL of cerebrospinal fluid before the 5th vaccination for cerebrospinal fluid cytokine testing, immune repertoire sequencing, and various immune cell phenotyping analyses.
5. Blood Routine: Absolute leukocyte count, absolute neutrophil count, relative neutrophil count, absolute lymphocyte count, relative lymphocyte count, absolute monocyte count, relative monocyte count, absolute eosinophil count, relative eosinophil count, absolute basophil count, relative basophil count, absolute red blood cell count, hemoglobin, absolute platelet count, C-

reactive protein.

6. Immune Cell Phenotyping: B lymphocyte subsets (CD3-CD19+), natural killer cells CD3-CD16+56+, T lymphocyte subsets (CD3+), helper T cells (CD3+ CD4+), suppressor T cells (CD3+ CD8+), helper T cell/suppressor T cell ratio.
7. Blood Biochemistry: Alanine aminotransferase, aspartate aminotransferase, alkaline phosphatase, γ-glutamyl transferase, total protein, albumin, globulin, total bilirubin, direct bilirubin, prealbumin, lactate dehydrogenase, urea, creatinine, uric acid, inorganic phosphorus, calcium, magnesium, potassium, sodium, chloride, glomerular filtration rate, glucose, β2-microglobulin.
8. Coagulation Routine: Prothrombin time, international normalized ratio, activated partial thromboplastin time, fibrinogen, thrombin time.
9. Urine Routine: Urine pH, urine protein, urine occult blood, urine leukocytes, urine glucose, urine specific gravity, urine ketones.
10. Pregnancy Test: Serum pregnancy test is required for women of childbearing potential during the screening period. Postmenopausal women may be exempted from testing.
11. Serum Virus Testing: Hepatitis C antibody, hepatitis B surface antigen, hepatitis B surface antibody, hepatitis B e antigen, hepatitis B e antibody, hepatitis B core antibody, human immunodeficiency virus antibody, Treponema pallidum specific antibody.
12. Serum Ig Levels: IgG, IgM, IgE, IgA.
13. Cytokine Testing: IL-1β, IL-2, IL-6, IL-8, IL-10, TNF-α.
14. Physical Fitness Assessment: Karnofsky Performance Status score, ECOG Performance Status score.
15. Quality of Life Assessment: FACT-G Quality of Life score, EORTC QLQ-30 Quality of Life questionnaire.
16. Adverse Event Recording: Investigators must record adverse events from the first DC vaccination, tracking until the adverse event reaches CTCAE 5.0, resolves, or the subject withdraws from the trial; all adverse events occurring from the first dose of the study drug until 8 weeks after the last dose should be reported. Adverse events occurring after 8 weeks that are considered related to the study drug should also be reported.
17. Concomitant Medication Recording: Investigators must record concomitant medications from enrollment, paying special attention to the use of hormones, chemotherapy drugs, radiotherapy doses, and targeted drugs, until the subject withdraws from the trial.
18. After the subject completes 5 injections, the investigator will decide whether to increase the number of injections based on the subject's tolerance and compliance; if increased, administer the DC vaccine every four weeks, but the total number of injections per course should not exceed 8.
19. For some subjects with good treatment tolerance, immunological response, and clinical response, peripheral blood will be drawn after completion of TMZ adjuvant chemotherapy to assess immune response. Based on the immune response assessment results, decide whether to administer 1-2 additional courses (5-8 injections per course) of DC vaccine intensification therapy. Simultaneously, draw blood once during and once after each course at appropriate times for safety and immunogenicity evaluation.
