# Supplementary material for: Personalized neoantigen-pulsed autologous dendritic cells in newly-diagnosed glioblastoma: a phase Ib trial
Source: Nat Commun. 2026 Jul 4;17:8302. doi: 10.1038/s41467-026-75066-w (PMC13469091; doi:10.1038/s41467-026-75066-w)
Supplement: Supplementary file 2 [file 41467_2026_75066_MOESM2_ESM.pdf]

Descriptions of Additional Supplementary Files

Supplementary Dataset 1: Summary of neoantigen peptides used in treated patients. This dataset provides a summary of tumor mutation burden, selected neoantigen-associated genes, HLA class I and II loci involved, and the number of manufactured neoantigen peptides for each treated patient. Patient-specific sheets provide detailed information on the neoantigen peptides used for each patient, including gene name, HLA allele, MHC-I and MHC-II epitope sequences, full peptide sequence, and peptide length.

Supplementary Dataset 2: Comparisons of baseline characteristics between enrolled and screen-failed patients. This dataset presents baseline demographic, clinical, surgical, and molecular characteristics of treated patients, consent-withdrawn patients, and patients excluded because fewer than five neoantigens were predicted. The dataset also includes the statistical comparisons between treated patients and each screen-failed subgroup.

Supplementary Dataset 3: Progression-free survival correlations with clinical variables and tumor molecular features. This dataset provides exploratory analyses of associations between progression-free survival and clinical variables, surgical factors, and tumor molecular features. For categorical variables, log-rank test results are provided; for continuous variables, univariate Cox proportional hazards regression results are shown as hazard ratios with 95% confidence intervals.

Supplementary Dataset 4: Quality control and release criteria for ZSNeo-DC. This dataset lists the quality control assays and release criteria used for ZSNeo-DC manufacturing, including viable cell concentration, cell viability, migratory capacity, dendritic cell phenotypic markers, non-DC contaminants, endotoxin, mycoplasma, and sterility testing.

Supplementary Dataset 5: Antibodies used for flow cytometry. This dataset provides details of antibodies used for flow cytometry experiments, including experiment type, marker, fluorophore, supplier, catalog number, clone, lot number, and dilution.
