# Supplementary material for: Personalized neoantigen-pulsed autologous dendritic cells in newly-diagnosed glioblastoma: a phase Ib trial
Source: Nat Commun. 2026 Jul 4;17:8302. doi: 10.1038/s41467-026-75066-w (PMC13469091; doi:10.1038/s41467-026-75066-w)
Supplement: Supplementary file 3 [file 41467_2026_75066_MOESM8_ESM.pdf]

nature portfolio

Corresponding author(s): Nan Ji & Xudong Cheng

Last updated by author(s): Jan 10, 2026

Reporting Summary

Nature Portfolio wishes to improve the reproducibility of the work that we publish. This form provides structure for consistency and transparency in reporting. For further information on Nature Portfolio policies, see our Editorial Policies and the Editorial Policy Checklist.

Statistics

For all statistical analyses, confirm that the following items are present in the figure legend, table legend, main text, or Methods section.

n/a	Confirmed	
☐	☒	The exact sample size (*n*) for each experimental group/condition, given as a discrete number and unit of measurement
☐	☒	A statement on whether measurements were taken from distinct samples or whether the same sample was measured repeatedly
☐	☒	The statistical test(s) used AND whether they are one- or two-sided<br>*Only common tests should be described solely by name; describe more complex techniques in the Methods section.*
☐	☒	A description of all covariates tested
☐	☒	A description of any assumptions or corrections, such as tests of normality and adjustment for multiple comparisons
☐	☒	A full description of the statistical parameters including central tendency (e.g. means) or other basic estimates (e.g. regression coefficient) AND variation (e.g. standard deviation) or associated estimates of uncertainty (e.g. confidence intervals)
☐	☒	For null hypothesis testing, the test statistic (e.g. *F*, *t*, *r*) with confidence intervals, effect sizes, degrees of freedom and *P* value noted<br>*Give P values as exact values whenever suitable.*
☒	☐	For Bayesian analysis, information on the choice of priors and Markov chain Monte Carlo settings
☒	☐	For hierarchical and complex designs, identification of the appropriate level for tests and full reporting of outcomes
☐	☒	Estimates of effect sizes (e.g. Cohen's *d*, Pearson's *r*), indicating how they were calculated

Our web collection on statistics for biologists contains articles on many of the points above.

Software and code

Policy information about availability of computer code

Data collection	Clinical data were collected using an electronic data capture system (Aidiyan Medical Technology Ltd., version 1.4.3). No custom software was used for clinical data collection.
Data analysis	WES reads were aligned to the human reference genome GRCh37/hg19 using BWA v0.7.17, and somatic variants were identified using Sentieon TNseq v202010.01. Variant annotation was performed using ANNOVAR v2020-06-08. Mutational signatures were analyzed using SomaticSignatures v2.44.0 and deconstructSigs v1.9.0 in R. Copy number alterations were estimated using DNAcopy v1.68.0 and visualized using Circos v0.69-9. Clonal architecture was inferred using PyClone v0.13.1, and phylogenetic trees were constructed using supraHex v1.34.0. HLA haplotypes were predicted using Polysolver v4 and HLAminer v1.4.<br>For RNA-seq analysis, raw reads were quality-controlled using fastp v0.20.1 and aligned to the human reference genome GRCh38/hg38 using HISAT2 v2.1.0. Gene expression was quantified using StringTie v1.3.4d.<br>For TCR repertoire analysis, raw TCRβ sequencing reads were processed using fastp v0.20.1 and aligned and assembled using MiXCR v4.2.2. TCR repertoire diversity and clonal frequency metrics were quantified using the immunarch R package v0.9.1.<br>Statistical analyses and figure generation were performed using R v4.4.0. No custom code was developed for this study.

For manuscripts utilizing custom algorithms or software that are central to the research but not yet described in published literature, software must be made available to editors and reviewers. We strongly encourage code deposition in a community repository (e.g. GitHub). See the Nature Portfolio guidelines for submitting code & software for further information.

Data

Policy information about availability of data

All manuscripts must include a data availability statement. This statement should provide the following information, where applicable:

- Accession codes, unique identifiers, or web links for publicly available datasets
- A description of any restrictions on data availability
- For clinical datasets or third party data, please ensure that the statement adheres to our policy

The raw WES, RNA-seq and TCR-seq data generated in this study have been deposited in the Genome Sequence Archive for Human (GSA-Human) of the China National Center for Bioinformation under accession code HRA014059 [https://ngdc.cncb.ac.cn/gsa-human/browse/HRA014059]. These human sequencing data are available under restricted access because they contain patient-derived genomic information and are subject to patient privacy, informed consent and institutional ethics requirements. Access can be obtained by applying through the GSA-Human data access system and completing the required data access agreement. Where additional approval is required, requests should be directed to the corresponding authors, Dr. Nan Ji and Dr. Xudong Cheng. Requests will be reviewed within approximately 30 business days, and approved access will be granted for the duration specified in the data access agreement.
The raw patient-level clinical data are protected and are not publicly available due to privacy and consent restrictions. De-identified clinical data, processed immunological data and other data underlying the results reported in this study are provided in the Source Data files. Source data are provided with this paper.

Research involving human participants, their data, or biological material

Policy information about studies with human participants or human data. See also policy information about sex, gender (identity/presentation), and sexual orientation and race, ethnicity and racism.

Reporting on sex and gender	Sex was not considered in the study design. Sex of participants was determined based on self-report. Both male and female patients were included in the study. Analyses were adjusted for sex, and no significant sex-related differences were observed (Tables S3,4). Written informed consent, including disclosure of sex information was obtained from all participants, in accordance with ethics committee approval.
Reporting on race, ethnicity, or other socially relevant groupings	Not applicable.
Population characteristics	Age, sex, KPS score at baseline, pre-operative volume of contrast-enhanced MRI lesion volume, post-operative volume of contrast-enhanced MRI lesion volume, extent of resection, presence of MGMT methylation, presence of TERT mutation, tumor mutation burden and immune subtype of tumor microenvironment.
Recruitment	Participants were recruited from the outpatient clinics of the Department of Neurosurgery at Beijing Tiantan Hospital. Patients with suspected or newly diagnosed GBM were screened by the study investigators according to the predefined eligibility criteria. Eligible patients were informed of the study procedures, potential risks and benefits, and voluntary nature of participation, and written informed consent was obtained before enrollment.<br>Because recruitment was conducted at a single tertiary neurosurgical center and participation required willingness to undergo personalized vaccine preparation and longitudinal immune monitoring, potential selection bias may have been introduced. Patients who consented to participate may have had better performance status, greater access to specialized care, or stronger motivation for experimental therapy than the broader nGBM population. These factors may limit the generalizability of the findings and could lead to overestimation of feasibility, adherence, and preliminary efficacy.
Ethics oversight	The study protocal received approval from the institutional review board and ethics committee of Beijing Tiantan Hospital (KY 2021-021-02).

Note that full information on the approval of the study protocol must also be provided in the manuscript.

Field-specific reporting

Please select the one below that is the best fit for your research. If you are not sure, read the appropriate sections before making your selection.

☒ Life sciences ☐ Behavioural & social sciences ☐ Ecological, evolutionary & environmental sciences

For a reference copy of the document with all sections, see nature.com/documents/nr-reporting-summary-flat.pdf

Life sciences study design

All studies must disclose on these points even when the disclosure is negative.

Sample size	This study is a phase I investigator-initiated clinical trial primarily designed to assess safety and feasibility. A total of 11 participants were enrolled, with only 10 participants completed a full treatment course. No formal sample size calculation was performed. The sample size was chosen based on feasibility considerations and typical numbers used in early-phase exploratory studies.
Data exclusions	One patient who did not receive one complete treatment course was excluded from the efficacy analysis according to the protocol.

Replication	Cell-based in vitro experiments were independently repeated and yielded similar results, supporting the reproducibility of these experimental findings. For assays involving patient-derived clinical samples, including ELISPOT and flow cytometry, each sample and condition was analyzed using technical triplicates where applicable to assess within-assay measurement precision. These technical replicates were averaged before patient-level analysis and were not treated as independent biological replicates. Independent biological replication of patient-specific ELISPOT and flow cytometry assays was limited by the availability of clinical samples and personalized neoantigen materials. No findings included in the manuscript failed reproducibility or technical quality checks.
Randomization	This is a phase I single-center study designed to assess safety and feasibility of DC vaccine. Randomization is not relevant.
Blinding	This is a phase I single-center study designed to assess safety and feasibility of DC vaccine. Blinding is not relevant.

Reporting for specific materials, systems and methods

We require information from authors about some types of materials, experimental systems and methods used in many studies. Here, indicate whether each material, system or method listed is relevant to your study. If you are not sure if a list item applies to your research, read the appropriate section before selecting a response.

Materials & experimental systems

n/a	Involved in the study
☐	☒ Antibodies
☐	☒ Eukaryotic cell lines
☒	☐ Palaeontology and archaeology
☒	☐ Animals and other organisms
☐	☒ Clinical data
☒	☐ Dual use research of concern
☒	☐ Plants

Methods

n/a	Involved in the study
☒	☐ ChIP-seq
☐	☒ Flow cytometry
☒	☐ MRI-based neuroimaging

Antibodies

Antibodies used	Detailed information of antibodies used in the current study were listed in Table S6
Validation	All primary antibodies used in the immune assays were validated for the corresponding species and application. Validation information was obtained from manufacturer datasheets. Links to each antibody's manufacturer datasheet are provided below:<br>https://files.dakewe.cn/crm/instruction/blg/368511.pdf<br>https://files.dakewe.cn/crm/instruction/blg/300419.pdf<br>https://files.dakewe.cn/crm/instruction/blg/317427.pdf<br>https://files.dakewe.cn/crm/instruction/blg/344723.pdf<br>https://files.dakewe.cn/crm/instruction/blg/304127.pdf<br>https://files.dakewe.cn/crm/instruction/blg/353207.pdf<br>https://files.dakewe.cn/crm/instruction/blg/329905.pdf<br>https://files.dakewe.cn/crm/instruction/blg/345021.pdf<br>https://www.bdbiosciences.com/content/dam/bdb/product_assets/product_pdf/singcolorpureantibody/apc/pdf_0/566762.pdf<br>https://files.dakewe.cn/crm/instruction/blg/310929.pdf<br>https://files.dakewe.cn/crm/instruction/blg/353727.pdf<br>https://files.dakewe.cn/crm/instruction/blg/305207.pdf<br>https://files.dakewe.cn/crm/instruction/blg/305323.pdf<br>https://files.dakewe.cn/crm/instruction/blg/305429.pdf<br>https://files.dakewe.cn/crm/instruction/blg/307626.pdf

Eukaryotic cell lines

Policy information about cell lines and Sex and Gender in Research

Cell line source(s)	KLE(CTCC-400-0001, Zhejiang meisen cells technology co., ltd, China),Homo sapiens,Female;<br>CFPAC-1(CTCC-400-0058, Zhejiang meisen cells technology co., ltd, China),Homo sapiens,Male
Authentication	Human cell lines used in this study were purchased in 2021 and authenticated by short tandem repeat (STR) profiling according to the cell identification standard in effect at that time (ASN-0002-2011), which included eight STR loci. The STR profiles were used to confirm cell line identity before experimental use, and no evidence of cell line misidentification was detected.
Mycoplasma contamination	We confirm that all cellines tested negative for mycoplasma contamination.
Commonly misidentified lines<br>(See ICLAC register)	We confirm that all cell lines used in the current study were not listed in the database of known misidentified cell lines maintained by the International Cell Line Authentication Committee (ICLAC).

Clinical data

Policy information about clinical studies

All manuscripts should comply with the ICMJE guidelines for publication of clinical research and a completed CONSORT checklist must be included with all submissions.

Clinical trial registration	Clinical trial information: NCT04968366 at Clinicaltrials.gov.
Study protocol	Full study protocol has been provided as additional review material during submission.
Data collection	Clinical data were collected using an electronic data capture (EDC) system (Aidiyan Medical Technology Ltd., version 1.4.3). Participants were recruited from the Department of Neurosurgery outpatient clinics at Beijing Tiantan Hospital between July 2021 and September 2023. Baseline clinical data were collected from the electronic medical record system at Beijing Tiantan Hospital. Immune monitoring data were collected 7 days after the third and fifth vaccine doses. MRI scans were performed at Beijing Tiantan Hospital at the following time points: preoperatively, within 3 days postoperatively, before the first vaccination, 7 days after the third vaccination, before the fifth vaccination, and every 3 months after the fifth dose until disease progression. Participants were followed from enrollment until 31 October 2025, with survival outcomes assessed every 3 months by investigators via telephone follow-up.
Outcomes	The primary objective of the study is to assess safety of ZSNeo-DC vaccine, using NCI CTCAE 5.0 to grade all adverse events. The secondary objectives included preliminary clinical efficacy and immunological efficacy of ZSNeo-DC. Clinical efficacy were assessed using PFS, OS, and 1-year survival rate. Immunological efficacy were assessed using ex vivo enzyme-linked immunospot (ELISPOT) assay to detec the number of subjects with specific IFN-gamma+ T cells in peripheral blood PBMC after treatment with their own tumor neoantigens.

Plants

Seed stocks	*Report on the source of all seed stocks or other plant material used. If applicable, state the seed stock centre and catalogue number. If plant specimens were collected from the field, describe the collection location, date and sampling procedures.*
Novel plant genotypes	*Describe the methods by which all novel plant genotypes were produced. This includes those generated by transgenic approaches, gene editing, chemical/radiation-based mutagenesis and hybridization. For transgenic lines, describe the transformation method, the number of independent lines analyzed and the generation upon which experiments were performed. For gene-edited lines, describe the editor used, the endogenous sequence targeted for editing, the targeting guide RNA sequence (if applicable) and how the editor was applied.*
Authentication	*Describe any authentication procedures for each seed stock used or novel genotype generated. Describe any experiments used to assess the effect of a mutation and, where applicable, how potential secondary effects (e.g. second site T-DNA insertions, mosiacism, off-target gene editing) were examined.*

Flow Cytometry

Plots

Confirm that:

☒ The axis labels state the marker and fluorochrome used (e.g. CD4-FITC).

☒ The axis scales are clearly visible. Include numbers along axes only for bottom left plot of group (a 'group' is an analysis of identical markers).

☒ All plots are contour plots with outliers or pseudocolor plots.

☒ A numerical value for number of cells or percentage (with statistics) is provided.

Methodology

Sample preparation	Flow Cytometry Cell Staining Protocol for Cell Surface Markers Cell Preparation: Harvest and wash the cells. Aliquot 5 × 10^5 cells into a flow cytometry tube. Staining Suspension: Resuspend the cell pellet in a suitable volume of Cell Staining Buffer and adjust the total volume to 50 µL. This yields a final cell concentration of 1.0 × 10^7 cells/mL. Antibody Staining: Add antibodies at the pre-determined optimal concentrations to the cell suspension. Mix the tubes thoroughly by gentle vortexing. Incubation: Incubate the stained cell suspension for 15 minutes at room temperature, ensuring the tubes are protected from light throughout the incubation period. Washing: Add 1 mL of 1× Phosphate Buffered Saline (PBS) to each tube to stop the staining reaction and dilute unbound antibodies. Centrifuge at 400g for 5 minutes. Carefully aspirate and discard the supernatant to remove excess antibodies. Post-Wash Resuspension: Resuspend the cell pellet in 300 µL of Cell Staining Buffer to a homogeneous single-cell suspension.
Instrument	Cytometer: BD FACSLyric
Software	BD FACSuite v1.4.1
Cell population abundance	Not applicable.
Gating strategy	All Acquired Events → [FSC-A / SSC-A] → Cells → [FSC-H / FSC-A] → Single Cells → [FSC-A / SSC-A] → Lymphocytes.

Gating strategy	Appropriate controls including unstained cells, single-color compensations, and fluorescence-minus-one (FMO) controls were used to establish gating thresholds.

☒ Tick this box to confirm that a figure exemplifying the gating strategy is provided in the Supplementary Information.
