# Supplementary material for: Personalized neoantigen-pulsed autologous dendritic cells in newly-diagnosed glioblastoma: a phase Ib trial
Source: Nat Commun. 2026 Jul 4;17:8302. doi: 10.1038/s41467-026-75066-w (PMC13469091; doi:10.1038/s41467-026-75066-w)
Supplement: Supplementary file 4 [file 41467_2026_75066_MOESM9_ESM.pdf]

nature portfolio

Peer Review File

Personalized neoantigen-pulsed autologous dendritic cells in newly-diagnosed glioblastoma: a phase Ib trial.

Corresponding Author: Professor Nan Ji

This file contains all reviewer reports in order by version, followed by all author rebuttals in order by version.

Version 0:

Reviewer comments:

Reviewer #1

(Remarks to the Author)
Summary:

The early-phase trial evaluated the safety and preliminary efficacy of the ZSNeo-DC vaccine, a personalized neoantigen-pulsed autologous dendritic cell vaccine, as an adjuvant treatment for newly diagnosed glioblastoma multiforme (nGBM). Overall, the ZSNeo-DC vaccine demonstrated good safety and promising efficacy signals, warranting further investigation in larger and later-stage trials.

General Comments:

The authors report results from a single-institution, early-stage trial, described as phase I, but is more of a phase 1b. The study was conducted in newly diagnosed GBM. After screening, there were a possible 17 candidates, but 6 were excluded because they had <5 neoantigens identified. The safety analysis includes all 11; the efficacy analysis focuses on 10. Baseline information for all 11 subjects is provided in extended table 3. Median age was 55 years, 0/10 IDH mutated, 3/10 were MGMT-methylated, 7/10 had TERT mutations. For efficacy analyses, 1 was excluded because he was only able to receive 4 of the first 5 doses because of scheduling issues during COVID. The one patient excluded was the oldest and 1 of 3 who had residual tumor volume after surgery, and MGMT was unavailable. This patient should have been included in the efficacy analyses.

The investigational product, ZSNeo-DC vaccine, is an autologous vaccine consisting of autologous DC differentiated from PBMC, and loaded with selected autologous neoantigens. After antigen loading, the DC were matured. Each dose consisted of 10 M cells based on pre-cryopreservation counts, that were injected i.d. The treatment plan was defined as 5 doses (rationale not provided), starting with 3 weekly injections during the 3 weeks after RT/TMZ, before starting maintenance TMZ, then q4 weeks for two more doses (5 total doses). This appears to correspond to weeks 17, 18, 19, 24, 28 post-surgery, but patients were allowed up to 3 more doses at weeks 32, 36, and 40 post-surgery. However, only 1 patient got 6 doses, 9 got 5, and 1 got 4 (excluded from the current efficacy analyses. We Are not told why patients did not receive the additional doses.

Key findings include:

1. Safety: As would be expected based on other reports, this DC vaccine was well-tolerated with minimal treatment-related adverse events.
2. Clinical Efficacy: Very encouraging with the caveat that this may not be a representative sample of nGBM patients
o Median progression-free survival (PFS) was 16.7 months from surgery, which is longer than the typical 8-9 months reported for nGBM patients receiving standard-of-care (SOC) treatment.
o The 12-month OS rate was 100% from surgery.
o Median overall survival (OS) was not reached after a median follow-up of 41.6 months from surgery
3. Immunological Efficacy:
o There were significant increases in the neoantigen-specific immune response (NSIR): median 7.2-fold increase after the 3rd vaccination (week19 post-surgery), and 10.3-fold after the 5th vaccination (week 28 post-surgery).
o There was a trend for longer PFS in patients with higher NSIR levels (above the median) compared to those with lower

NSIR levels.
4. T Cell Activation: After vaccination, peripheral T cell phenotypes showed greater numbers of activated cells compared to exhausted cells, which positively correlated with NSIR and extended PFS.
5. TCR Repertoire Changes: ZSNeo-DC vaccination increased T cell receptor (TCR) repertoire evenness, driven by the expansion of newly emerging TCR clones. This increase was associated with better immune response and extended PFS.
6. Pseudo-progression: Pseudo-progression was observed in one patient, which correlated with a strong immune response and extended PFS.
7. Potential for Combination Therapy: Some patients who subsequently received immune checkpoint inhibitors (ICI) after tumor progression showed stable disease or complete remission, suggesting that the vaccine may enhance GBM's response to ICI therapies.

Abstract: Line 27-28: 11 patients completed 4 injections over 8 weeks. All 11 were appropriately included in the safety analysis, but all 11 should have been included in the efficacy analysis as well. The one patient was excluded who received 4 injections, but was the oldest and 1 of only 3 who had residual tumor following surgical resection.

Abstract: Line 32: Suggest using other than the word “shift” which implies that the existing cells changed phenotype, when an alternative explanation is entry into the circulation of more cells from activated clones. Suggest that the sentence should read, “Based on the phenotypes of circulating T cells, vaccination was followed by a substantial increase in the ratio of activated to exhausted cell numbers.

Paragraph #1 Lines 41-44: Suggest making it clear that SOC per Stupp 2005 report is surgery followed by concurrent radiation therapy (RT) and temozolomide (TMZ), then adjuvant TMZ, with consideration of tumor-treating fields (TTF) in patients who have no evidence of progression or pseudoprogression.

Paragraph #1Lines 44-45: Clarify that survival rates are from the date of surgery based on clinical trials that enrolled patients before initiating RT/TMZ. Inclusion of data from trials that were limited to patients without evidence of disease progression or pseudoprogression following RT/TMZ (such as Stupp TTF trial and Liau DCVax trial skew the data because of a more selected population.

Paragraph #2, lines 52-54, is not well-referenced. The following trials of autologous DC and autologous tumor antigens in primary GBM should be cited and perhaps contrasted to this trial in the Discussion section.

Liau DCVax 2023: (already included), Additionally her 2018 publication could be considered because provides more details about the study feasibility and patient population.

Inoges 2017: single-arm 31-patient Spanish trial of DC plus autologous tumor lysate. Treatment was started before RT/TMZ in this trial.

Buchroithner 2018 This is a more appropriate citation for clinical information than current reference 12. Austrian 76-patient randomized trial of DC plus autologous tumor lysate using mature DC.

Ridolfi 2024 Italian trial, initial report (n=11) of adaptive phase 2 DC plus autologous tumor lysate, mature DC (CombiG-vax)

Latzer 2024: (already included but significance not noted) Includes 70 primary GBM patients treated with autologous DC and neoantigens. Median # of neoantigens was 19 with range from 6 to 24.

Bota 2025: (also an initial report from 2022) Autologous DC loaded with antigens from short-term autologous tumor cell line, n=57.

Lines 70 and 83. Please delete the word “novel”

Vaccine and Study overview, lines 82-98 or in Methods lines 302-318: There was no rationale or basis provided for the various treatment parameters. Please provide the following information: [1] How many patients were planned for treatment? There was no dose escalation, and safety alone could have been established with fewer patients. Perhaps it would be better to refer to this as a Phase 1b trial rather than Phase 1 since, based on the literature, there was little safety concern for an autologous vaccine. [2] How was the 10 M dose chosen? [3] How was the vaccine schedule chosen? [4] How was i.d. route chosen? [5] How was the 5 neoantigen threshold selected? It would appear that dose and schedule were used based on the publications, which is an acceptable explanation. [6] Why was evaluability for efficacy based on treatment per protocol defined as 5 injections rather than 3 or 4, or even 1. Was this prespecified? 1 patient got 6 injections, 9 got 5 injections, 1 got 4 injections. All 11 were appropriately included in the safety analysis, but all 11 should have been included in the efficacy analysis as well. The one patient excluded was the oldest, and 1 of only 3 who had residual tumor following surgical resection; therefore, he would be expected to have a worse prognosis.

Treatment schedule: Lines 92 and 316 and Figure 2. It would be helpful to have the text and figure match. It appears this would be correct: A complete treatment course planned for injections at weeks 3, 4, 5, 8, and 12 post-concurrent RT/TMZ corresponding to weeks 17, 18, 19, 24, and 28 post-surgery. Additional injections were permitted at weeks 16, 20, and 24 post-concurrent RT/TMZ, corresponding to weeks 32, 36, and 40. The authors should explain why only 1 patient received more than 5 injections.

Discussion: Concerning feasibility, the authors should address the fact that after screening disqualified 22 potential subjects (withdrawal, poor KPS, not GBM etc), of the remaining 18, 6 (33%) had an insufficient number of antigens and therefore could not proceed.

Data and Figures: There is an excellent amount of data presented. Probably the least helpful is the safety table, whereas extended Table 3 is critical for understanding prognostic baseline features, and should be included in the main manuscript.

References: These appear to have been generated by AI rather than curated by the authors. In many instances, text is not directly supported by the references, and conversely, the conclusions and findings of the references are not reflected in the text. As noted above, there are several key references describing clinical trials testing autologous DC loaded with autologous tumor antigens that are not cited or discussed.

Reviewer #2

(Remarks to the Author)
This is a manuscript detailing the results of a phase I trial evaluating a personalized neoantigen-pulsed autologous dendritic cell vaccine as adjuvant treatment in patients with newly diagnosed GBM with a conclusion of safety of ZSNeo-DC and a reported "encouraging" efficacy signal.

I would caution the authors regarding the use of "encouraging" and "potentially promising immunotherapy" in describing their ZSNeo-DC given the very small sample size in addition to the favorable baseline population they selected (example >90% resection of contrast enhancing disease).

Can the authors clarify why eligibility criteria required that patients have 90% or greater of their contrast enhancing resected?

There is significant heterogeneity in the management of the patients in this small study which makes the survival data harder to interpret.

For patient R01, was the patient symptomatic? I'm unclear as to why the patient was treated with bevacizumab "for controlling pseudoprogression" which has implications on imaging interpretation unless the patient was symptomatic and requiring high dose steroids.

For patient R02, what was the justification to treat the patient with pembrolizumab and bevacizumab?

What was the reason 10 patients withdrew consent?

Why did so many patients receive bevacizumab after first progression? Were there no other lines of therapy to consider as an option (example CCNU)?

For patient R10, why was progression not called until after treatment with bevacizumab?

For the serial imaging, were any advanced imaging techniques used like perfusion and spectroscopy to aid in the review particularly those patients who had changes that were presumed to pseudoprogression?

Reviewer #3

(Remarks to the Author)
This Phase 1 study considers a novel personalized vaccine for treatment of newly-diagnosed glioblastoma, which has substantial unmet need for therapeutic advances since the current standard of care was established two decades ago. The sample size is small, but the results are promising and merit further investigation of the therapy in larger trials. My concerns mostly relate to additional context needed to interpret the results in a way that makes clearer (a) how the safety and efficacy of the therapy relate to those of competing therapies, and (b) how further studies of this vaccine should be designed. With this additional context I think this paper can be an excellent contribution to the field.

Major Concerns:

1. To better characterize the analyzed patient population and its generalizability, please further describe (a) if there are any observed differences between patients who withdrew consent vs. those who consented, (b) any factors (clinical or technical) associated with having fewer than 5 predicted neoantigen peptides, and (c) the reason for the 1 manufacturing failure.

2. The abstract provides an incomplete and somewhat misleading summary of the AEs, especially the sentence "Treatment was well tolerated, with only two (18.2%) patients reporting grade-1/2 fever as treatment-related adverse events." which seems to imply that there were no other AEs aside from fever. In addition, in the Results and Discussion sections it would be helpful to comment in greater detail on how the observed AE rates compare specifically to those for other approved or investigational anti-glioma vaccines or competing therapies.

3. Regarding the dosing, it would be helpful to explain (a) the rationale determining the dose amounts and schedule and any uncertainty regarding the potential superiority of other dosing schemes, (b) why no patients were given more than 5 doses despite this being mentioned as a possibility in the protocol (i.e. why specifically did the subjects' tolerance and compliance

patterns lead the investigator(s) to decide never to give more than 5 injections?).

4. Based on the Kaplan-Meier curves it appears that patients who experienced an "unfavorable" vaccine response (in terms of Gini coefficient and/or other metrics) had PFS rates comparable to published rates on the Stupp regimen SOC. It would be helpful to comment explicitly on this point specifically to contextualize survival differences by vaccine response.

5. I understand the sample size is very limited to answer this question, but with that in mind was there any evidence that MGMT methylation status or other tumor genomic markers were associated with vaccine response?

Minor concerns:

1. On page 3 "stupp's" should be capitalized.

2. On page 7 TME acronym (I assume tumor microenvironment) should be defined.

Version 1:

Reviewer comments:

Reviewer #1

(Remarks to the Author)
Nature Communications. (revised title): Zhang Y, Chi X, Liu K et al. Personalized neoantigen-pulsed autologous dendritic cells vaccine as an adjuvant treatment for patients with newly-diagnosed glioblastoma multiforme: a phase IB trial.

General Comments:

The authors have provided a revised manuscript that adequately addresses most of the issues previously raised by reviewers. This includes citing several other relevant GBM vaccine studies that were previously published.

Specific comments;

1.Line 55: Please exchange the existing reference 8 (Erhart F, Buchroithner J Reitermeir R et al. with their other 2018 reference: Buchroithner J, Erhart F, Pichler J, et al. Audencel immunotherapy based on dendritic cells has no effect on overall and progression-free survival in newly diagnosed glioblastoma: A phase II randomized trial. Cancers (Basel). 2018;10:372. This is the publication that actually describes the randomized clinical trial and its results. The Erhart reference, which is focused on the immunologic correlations, is appropriate for the current 8 on line 254.

2. Lines 66-67 are referring to neoantigen vaccines that have been tested in glioma patients. References 23 and 24 appear to be appropriate, but reference 25 (Grassl N et al) describes treatment with H3K27M-specific long peptide vaccine (H3K27M-vac) and is being targeted as a shared tumor-associated antigen rather than patient-specific neoantigens. It is not appropriate to characterize the study by Latzer P et al as “pilot study.”

3. Lines 225-226. The first line of the discussion should be revised for accuracy and clarity. DCs themselves do not constitute a vaccine. DC vaccines have not been tested in low-grade gliomas. The terms “cancer vaccines” and “tumor vaccines” imply anti-tumor or anti-cancer activity. For example, one might say “Many clinical trials have been conducted utilizing dendritic cell vaccines to treat malignant gliomas.” references

4. Discussion Lines 226,242,246. In previous comments, the reviewer erroneously advised that the Latzer P publication from 2024 (reference 24) described primary GBM patients treated with autologous DC and neoantigens. In fact, as clearly described in that manuscript, these were not dendritic cell vaccines; their vaccines consisted of patient-specific neoantigen peptides (range of 6 to 24) that were injected with adjuvant GM-CSF. Therefore, reference 24 should be removed from the superscripted references on lines 226, 242, and 246.

5. Discussion. Latzer et al. previously reported encouraging clinical outcomes with no safety concerns in a neoantigen GBM trial that was a much larger clinical study of GBM personalized neoantigen vaccines than described in the current report (70 vs 11 primary GBM patients). The results of that report should be briefly summarized in the discussion. Please comment that despite the theoretical advantages of antigen delivery by DC, comparable results might be obtained with other neoantigen vaccine strategies.

6. Discussion. The authors may want to note that variations in dendritic cell manufacturing may account for some of the differences between studies, and that the DC in the other studies cited in this article did not load antigens in the presence of anti-PDL-1 and a CD40 agonist, and not all used cytokines to induce maturation.

7. References. The authors should use the preferred style of the journal rather than copying the citations from PubMed or other sources. For example, some references have capitalization of words other than articles or prepositions in the titles while others are all lower case.

Reviewer #2

(Remarks to the Author)
I appreciate the authors' thoughtful response to all the reviewers' comments. I do not have any additional comments. I would support acceptance of this revised manuscript.

Reviewer #3

(Remarks to the Author)
I appreciate the author's responsiveness to my previous comments and I am mostly satisfied with the revision. I only have two further comments which should be easy to address.

1. Response Table 1 is interesting and relevant to the interpretation of the trial results. It should be included as an Extended Data Table and discussed briefly in the Results section (both the lack of apparent differences between the enrolled/consent-withdrawn patients and the factors associated with low number of predicted neoantigents).

2. To my understanding, there were no manuscript changes in my previous comment 3 about the dosing rationale. Please include in the manuscript a brief discussion of these issues and some of the references (if you're past journal limits on manuscript length, this could be included as Supplementary Material).

Open Access This Peer Review File is licensed under a Creative Commons Attribution 4.0 International License, which permits use, sharing, adaptation, distribution and reproduction in any medium or format, as long as you give appropriate credit to the original author(s) and the source, provide a link to the Creative Commons license, and indicate if changes were made.
In cases where reviewers are anonymous, credit should be given to 'Anonymous Referee' and the source.
The images or other third party material in this Peer Review File are included in the article’s Creative Commons license, unless indicated otherwise in a credit line to the material. If material is not included in the article’s Creative Commons license and your intended use is not permitted by statutory regulation or exceeds the permitted use, you will need to obtain permission directly from the copyright holder.
To view a copy of this license, visit https://creativecommons.org/licenses/by/4.0/

Response to Reviewers

Manuscript title: Personalized neoantigen-pulsed autologous dendritic cells vaccine as an adjuvant treatment for patients with newly-diagnosed glioblastoma multiforme: a phase Ib trial.

Manuscript ID: NCOMMS-25-101455

We thank the reviewers and editor for their constructive comments.

We have carefully revised the manuscript and addressed all concerns.

REVIEWER COMMENTS

Reviewer #1 (Remarks to the Author):

Summary:

The early-phase trial evaluated the safety and preliminary efficacy of the ZSNeo-DC vaccine, a personalized neoantigen-pulsed autologous dendritic cell vaccine, as an adjuvant treatment for newly diagnosed glioblastoma multiforme (nGBM). Overall, the ZSNeo-DC vaccine demonstrated good safety and promising efficacy signals, warranting further investigation in larger and later-stage trials.

General Comments:

The authors report results from a single-institution, early-stage trial, described as phase I, but is more of a phase 1b. The study was conducted in newly diagnosed GBM. After screening, there were a possible 17 candidates, but 6 were excluded because they had <5 neoantigens identified. The safety analysis includes all 11; the efficacy analysis focuses on 10. Baseline information for all 11 subjects is provided in extended table 3. Median age was 55 years, 0/10 IDH mutated, 3/10 were MGMT-methylated, 7/10 had TERT mutations. For efficacy analyses, 1 was excluded because he was only able to receive 4 of the first 5 doses because of scheduling issues during COVID. The one patient excluded was the oldest and 1 of 3 who had residual

tumor volume after surgery, and MGMT was unavailable. This patient should have been included in the efficacy analyses.

The investigational product, ZSNeo-DC vaccine, is an autologous vaccine consisting of autologous DC differentiated from PBMC, and loaded with selected autologous neoantigens. After antigen loading, the DC were matured. Each dose consisted of 10 M cells based on pre-cryopreservation counts, that were injected i.d. The treatment plan was defined as 5 doses (rationale not provided), starting with 3 weekly injections during the 3 weeks after RT/TMZ, before starting maintenance TMZ, then q4 weeks for two more doses (5 total doses). This appears to correspond to weeks 17, 18, 19, 24, 28 post-surgery, but patients were allowed up to 3 more doses at weeks 32, 36, and 40 post-surgery. However, only 1 patient got 6 doses, 9 got 5, and 1 got 4 (excluded from the current efficacy analyses. We Are not told why patients did not receive the additional doses.

Key findings include:

1.Safety: As would be expected based on other reports, this DC vaccine was well-tolerated with minimal treatment-related adverse events.
2.Clinical Efficacy: Very encouraging with the caveat that this may not be a representative sample of nGBM patients
oMedian progression-free survival (PFS) was 16.7 months from surgery, which is longer than the typical 8-9 months reported for nGBM patients receiving standard-of-care (SOC) treatment.
oThe 12-month OS rate was 100% from surgery.
oMedian overall survival (OS) was not reached after a median follow-up of 41.6 months from surgery
3.Immunological Efficacy:
oThere were significant increases in the neoantigen-specific immune response (NSIR): median 7.2-fold increase after the 3rd vaccination (week19 post-surgery), and 10.3-fold after the 5th vaccination (week 28 post-surgery).
oThere was a trend for longer PFS in patients with higher NSIR levels (above the median) compared to those with lower NSIR levels.
4.T Cell Activation: After vaccination, peripheral T cell phenotypes showed

greater numbers of activated cells compared to exhausted cells, which positively correlated with NSIR and extended PFS.

5. TCR Repertoire Changes: ZSNeo-DC vaccination increased T cell receptor (TCR) repertoire evenness, driven by the expansion of newly emerging TCR clones. This increase was associated with better immune response and extended PFS.

6. Pseudo-progression: Pseudo-progression was observed in one patient, which correlated with a strong immune response and extended PFS.

7. Potential for Combination Therapy: Some patients who subsequently received immune checkpoint inhibitors (ICI) after tumor progression showed stable disease or complete remission, suggesting that the vaccine may enhance GBM's response to ICI therapies.

Abstract: Line 27-28: 11 patients completed 4 injections over 8 weeks. All 11 were appropriately included in the safety analysis, but all 11 should have been included in the efficacy analysis as well. The one patient was excluded who received 4 injections, but was the oldest and 1 of only 3 who had residual tumor following surgical resection.

Response 1:

We appreciate the reviewer's assessment and suggestion. Including all 11 patients, regardless of their protocol completion status, would indeed ensure a more unbiased and comprehensive evaluation of the vaccine's efficacy. Accordingly, we have included patient R04 in the efficacy analysis for this revision, and have made corresponding updates in the revised manuscript, with changes indicated in bold below.

Changes in manuscript:

1. Page 1, Line 28:
   The sentence "Ten patients completed one treatment course and were evaluable for efficacy." has been deleted from the Abstract.
   The following sentence has been changed: "Median progression-free survival (PFS) was **16.2** months, median overall survival (OS) was not reached, and 12-month OS rate was 100% from surgery."
2. Page 3, Line 108:
   The following sentence has been changed: "Due to one patient failed in the DC generation, 11 patients were eventually administered with the vaccine, and were included in the safety analysis **and efficacy evaluation.**"

The sentence “10 patients completed a treatment course and were included in the efficacy evaluation as per the protocol.” has been deleted.

3. Page 4, Line 142:
   The following sentence has been changed: “Kaplan-Meier analysis revealed that the median PFS was **16.2** months (95% CI: 10.3 months-not reached), median OS was not reached (95% CI: 23.1 months-not reached), and the 12-month OS rate was 100% from surgery (Figure 2B-C).”
4. Page 4, Line 166:
   Added the following sentence: “10 patients completed the full treatment course, and peripheral blood samples were collected according to the schedule for the evaluation of immunological efficacy.”
5. Page 6, Line 232:
   The following sentence has been changed: “The median PFS was **16.2** months (95% CI: 10.3 months-not reached), and median OS had not been reached after a median follow-up of 41.6 months.”

Abstract: Line 32: Suggest using other than the word “shift” which implies that the existing cells changed phenotype, when an alternative explanation is entry into the circulation of more cells from activated clones. Suggest that the sentence should read, “Based on the phenotypes of circulating T cells, vaccination was followed by a substantial increase in the ratio of activated to exhausted cell numbers.

Response 2:

We sincerely appreciate this precise suggestion regarding the terminology. This has been revised accordingly, with changes indicated in bold below.

Changes in manuscript:

1. Page 1, Line 31:
   The following sentence has been changed: “**Following vaccination, both the activated**-to-**exhausted ratio of effector/central memory T cells** and **the clonal evenness** of **the** T cell receptor repertoire **increased significantly** in peripheral **blood; these parameters** positively correlated with NISR and PFS.”
2. Page 5, Line 184:
   The following sentence has been changed: “These findings indicate that ZSNeo-DC therapy may promote **phenotypic conversion** from exhaustion to

activation in peripheral effector/CM T cell populations. **Accordingly**, we utilized the **activated-to-exhausted** ratios in these T cell populations to **quantify this phenotypic alteration**, and **found that** they **positively correlated** with NSIR (Figure 4C) and extended PFS (Figure 4D), supporting **the** clinical relevance of peripheral T cell phenotype **remodeling following** ZSNeo-DC vaccination."

3. Page 6, Line 235:
   The following sentence has been changed: "With respect to immune response, the vaccine elicited a median increase of 7.23-fold and 10.33-fold in NSIR respectively at C3D7 and C5D7, promoted **phenotypic conversion from** exhaustion- to- activation in peripheral effector/CM T cells and induced a dramatic increase in peripheral TCR repertoire evenness."
4. Page 6, Line 257:
   The following sentence has been changed: "However, **a** higher **activated-to- exhausted ratio** in peripheral effector/CM T cells (Figure 4C-D), **as well as** a more balanced **clonal** space (Figure 5B-C and Extended Data Figure 6A-B) or newly emerging clones (Figure 5F-G and Extended Data Figure 6E-F) in **the** peripheral T cell repertoire**,** correlated with better response to ZSNeo-DC therapy."

Paragraph #1 Lines 41-44: Suggest making it clear that SOC per Stupp 2005 report is surgery followed by concurrent radiation therapy (RT) and temozolomide (TMZ), then adjuvant TMZ, with consideration of tumor-treating fields (TTF) in patients who have no evidence of progression or pseudoprogression.

Response 3:

We apologize for the confusion caused by our imprecise wording. This has been revised accordingly, with changes indicated in bold below.

Changes in manuscript:

1. Page 2, Line 41:
   The following sentence has been changed: "The standard-of-care (SOC) paradigm for GBM treatment involves maximal surgical resection followed by radiation therapy combined with temozolomide (TMZ)-based concurrent and adjuvant chemotherapy, **with consideration of tumor-treating fields (TTFs) for patients who have no evidence of progression following**

radiochemotherapy[2,3]."

Paragraph #1Lines 44-45: Clarify that survival rates are from the date of surgery based on clinical trials that enrolled patients before initiating RT/TMZ. Inclusion of data from trials that were limited to patients without evidence of disease progression or pseudoprogression following RT/TMZ (such as Stupp TTF trial and Liau DCVax trial skew the data because of a more selected population.

Response 4:

We sincerely appreciate the reviewer's emphasis on this critical methodological distinction. This has been revised accordingly, with changes indicated in bold below.

Changes in manuscript:

1. Page 2, Line 44:
   The following sentence has been changed: "However, these standard therapies provide only modest survival benefits, yielding a median overall survival of 15-20 months and a 5-year survival rate not exceeding 10% **from the date of surgery**"
2. Page 7, Line 285:
   The following sentence has been changed: "**Preliminary** efficacy **signals may be confounded by selection bias and post-progression management variability, requiring validation in randomized controlled** trials."

Paragraph #2, lines 52-54, is not well-referenced. The following trials of autologous DC and autologous tumor antigens in primary GBM should be cited and perhaps contrasted to this trial in the Discussion section.

Liau DCVax 2023: (already included), Additionally her 2018 publication could be considered because provides more details about the study feasibility and patient population.

Inoges 2017: single-arm 31-patient Spanish trial of DC plus autologous tumor lysate. Treatment was started before RT/TMZ in this trial.

Buchroithner 2018 This is a more appropriate citation for clinical

information than current reference 12. Austrian 76-patient randomized trial of DC plus autologous tumor lysate using mature DC.

Ridolfi 2024 Italian trial, initial report (n=11) of adaptive phase 2 DC plus autologous tumor lysate, mature DC (CombiG-vax)

Latzer 2024: (already included but significance not noted) Includes 70 primary GBM patients treated with autologous DC and neoantigens. Median # of neoantigens was 19 with range from 6 to 24.

Bota 2025: (also an initial report from 2022) Autologous DC loaded with antigens from short-term autologous tumor cell line, n=57.

Response 5:

We are deeply grateful to the reviewer for pointing out these key references. These trials represent important DC-based immunotherapies for GBM treatment. We have cited these references in the introduction section of the revised manuscript, as follows.

Changes in manuscript:

1. Page 2, Line 52:
   The following references has been changed: "These endeavors have yielded encouraging clinical efficacy in treating this deadly brain cancer[5-10]. Among these vaccines, DCvax, a personalized DC vaccine pulsed with autologous tumor cell lysate, has showed survival benefit in a phase 3 trial[5]. Nevertheless, despite the promising results of DCvax and other tumor-lysate DC vaccines[5,8,11,12], the uncertainty surrounding tumor antigens poses challenges in precise monitoring of immune response and raises concerns about off-tumor toxicity[13]."

Reference

5. Liau LM, Ashkan K, Brem S, et al. Association of Autologous Tumor Lysate-Loaded Dendritic Cell Vaccination With Extension of Survival Among Patients With Newly Diagnosed and Recurrent Glioblastoma: A Phase 3 Prospective Externally Controlled Cohort Trial. JAMA Oncol 2023;9(1):112-121. DOI: 10.1001/jamaoncol.2022.5370.
6. Liau LM, Ashkan K, Tran DD, et al. First results on survival from a large Phase 3 clinical trial of an autologous dendritic cell vaccine in newly diagnosed glioblastoma. J Transl Med 2018;16(1):142. DOI: 10.1186/s12967-

018-1507-6.
7. Inoges S, Tejada S, de Cerio AL, et al. A phase II trial of autologous dendritic cell vaccination and radiochemotherapy following fluorescence-guided surgery in newly diagnosed glioblastoma patients. J Transl Med 2017;15(1):104. DOI: 10.1186/s12967-017-1202-z.
8. Erhart F, Buchroithner J, Reitermaier R, et al. Immunological analysis of phase II glioblastoma dendritic cell vaccine (Audencel) trial: immune system characteristics influence outcome and Audencel up-regulates Th1-related immunovariables. Acta Neuropathol Commun 2018;6(1):135. DOI: 10.1186/s40478-018-0621-2.
9. Ridolfi L, Gurrieri L, Riva N, et al. First step results from a phase II study of a dendritic cell vaccine in glioblastoma patients (CombiG-vax). Front Immunol 2024;15:1404861. DOI: 10.3389/fimmu.2024.1404861.
10. Bota DA, Taylor TH, Piccioni DE, et al. Phase 2 study of AV-GBM-1 (a tumor-initiating cell targeted dendritic cell vaccine) in newly diagnosed Glioblastoma patients: safety and efficacy assessment. J Exp Clin Cancer Res 2022;41(1):344. DOI: 10.1186/s13046-022-02552-6.
11. Everson RG, Hugo W, Sun L, et al. TLR agonists polarize interferon responses in conjunction with dendritic cell vaccination in malignant glioma: a randomized phase II Trial. Nat Commun 2024;15(1):3882. DOI: 10.1038/s41467-024-48073-y.
12. Hu JL, Omofoye OA, Rudnick JD, et al. A Phase I Study of Autologous Dendritic Cell Vaccine Pulsed with Allogeneic Stem-like Cell Line Lysate in Patients with Newly Diagnosed or Recurrent Glioblastoma. Clin Cancer Res 2022;28(4):689-696. DOI: 10.1158/1078-0432.CCR-21-2867.
13. Stone JD, Harris DT, Kranz DM. TCR affinity for p/MHC formed by tumor antigens that are self-proteins: impact on efficacy and toxicity. Curr Opin Immunol 2015;33:16-22. DOI: 10.1016/j.coi.2015.01.003.

Furthermore, as suggested, we have compared our findings with these benchmark studies in the Discussion section of the revised manuscript, as detailed below.

Changes in manuscript:

1. Page 6, Line 241:
   The following sentence has been added: “ZSNeo-DC demonstrated a favorable safety profile, with only two grade 1/2 febrile TRAEs and no

injection site reactions or fatigue commonly reported with DC therapy[10,24,40]. Consistent with previous reports[6,7,40], the most common AE was lymphocytopenia, recorded in 63.6% (7/11) of participants and likely related to standard therapy. Notably, no serious TRAEs were observed in this cohort, aligning with the extremely low incidence (0.3%-2.1%) reported in two large cohorts of DC therapy[6,24]. Together, the safety profile of ZSNeo-DC is comparable to that of these landmark studies, underscoring the safety advantage of DC therapy for GBM treatment."

Reference

6. Liau LM, Ashkan K, Tran DD, et al. First results on survival from a large Phase 3 clinical trial of an autologous dendritic cell vaccine in newly diagnosed glioblastoma. J Transl Med 2018;16(1):142. DOI: 10.1186/s12967-018-1507-6.

7. Inoges S, Tejada S, de Cerio AL, et al. A phase II trial of autologous dendritic cell vaccination and radiochemotherapy following fluorescence-guided surgery in newly diagnosed glioblastoma patients. J Transl Med 2017;15(1):104. DOI: 10.1186/s12967-017-1202-z.

10. Bota DA, Taylor TH, Piccioni DE, et al. Phase 2 study of AV-GBM-1 (a tumor-initiating cell targeted dendritic cell vaccine) in newly diagnosed Glioblastoma patients: safety and efficacy assessment. J Exp Clin Cancer Res 2022;41(1):344. DOI: 10.1186/s13046-022-02552-6.

24. Latzer P, Zelba H, Battke F, et al. A real-world observation of patients with glioblastoma treated with a personalized peptide vaccine. Nat Commun 2024;15(1):6870. DOI: 10.1038/s41467-024-51315-8.

40. Shamshiripour P, Nikoobakht M, Mansourinejad Z, Ahmadvand D, Akbarpour M. A comprehensive update to Dendritic Cell therapy for glioma: a systematic review and meta-analysis. Expert Rev Vaccines 2022;21(4):513-531. DOI: 10.1080/14760584.2022.2027759.

Lines 70 and 83. Please delete the word "novel"

Response 6:

Thanks for your suggestion. We have deleted the word in the revised manuscript.

Changes in manuscript:

1. Page 2, Line 71:

The following sentence has been changed: “To address these issues, we developed a personalized DC vaccine (ZSNeo-DC), which was loaded with multiple neoantigen peptides to generate a robust and tumor-specific immune response while avoiding immune evasion.”

2. Page 2, Line 84:

   The following sentence has been changed: “The ZSNeo-DC vaccine adopted a DC design that combined a neoantigen-prediction algorithm based on tumor tissue/blood multi-omic data (Extended Data Figure 1), and immune cell engineering for DC modification to enhance the abilities in activating neoantigen-specific T cells (Figure 1A and Extended Data Figure 2-3).”

Vaccine and Study overview, lines 82-98 or in Methods lines 302-318: There was no rationale or basis provided for the various treatment parameters. Please provide the following information: [1] How many patients were planned for treatment? There was no dose escalation, and safety alone could have been established with fewer patients. Perhaps it would be better to refer to this as a Phase 1b trial rather than Phase 1 since, based on the literature, there was little safety concern for an autologous vaccine. [2] How was the 10 M dose chosen? [3] How was the vaccine schedule chosen? [4] How was i.d. route chosen? [5] How was the 5 neoantigen threshold selected? It would appear that dose and schedule were used based on the publications, which is an acceptable explanation. [6] Why was evaluability for efficacy based on treatment per protocol defined as 5 injections rather than 3 or 4, or even 1. Was this prespecified? 1 patient got 6 injections, 9 got 5 injections, 1 got 4 injections. All 11 were appropriately included in the safety analysis, but all 11 should have been included in the efficacy analysis as well. The one patient excluded was the oldest, and 1 of only 3 who had residual tumor following surgical resection; therefore, he would be expected to have a worse prognosis.

Response 7:

We sincerely appreciate these suggestions. We have added the required information to the Methods section (Page 8, Line 328). Below is our point-by-point response to the specific concerns raised.

[1] How many patients were planned for treatment? There was no dose

escalation, and safety alone could have been established with fewer patients. Perhaps it would be better to refer to this as a Phase 1b trial rather than Phase 1 since, based on the literature, there was little safety concern for an autologous vaccine.

A total of 10 patients were planned for treatment in this study. We anticipated that this sample size would provide sufficient preliminary data on clinical and immunological efficacy beyond safety profile.

As suggested, we have updated the manuscript to refer to this study as a Phase 1b trial.

Changes in manuscript:

1. Page 1, Line 2:
   Title has been changed: "Personalized neoantigen-pulsed autologous dendritic cells vaccine as an adjuvant treatment for patients with newly-diagnosed glioblastoma multiforme: a phase Ib trial."
2. Page 1, Line 23:
   The following sentence has been changed: "Here, we present results of a phase 1b trial, which evaluated the safety and preliminary efficacy of neoantigen-pulsed autologous dendritic cells (ZSNeo-DC) as an adjuvant treatment for nGBM."
3. Page 2, Line 76:
   The following sentence has been changed: "Therefore, we initiated a phase 1b study to evaluate its safety and preliminary efficacy, when it was administered alongside the SOC therapy in newly-diagnosed GBM (nGBM) patients."
4. Page 3, Line 87:
   The following sentence has been changed: "To investigate safety and preliminary efficacy of ZSNeo-DC, we initiated a phase 1b trial on the treatment of nGBMs using the vaccine as an adjuvant therapy, in combination with SOC post-operative radiochemotherapy."
5. Page 6, Line 228:
   The following sentence has been changed: "In this phase 1b pilot study, we evaluated the safety and preliminary efficacy of a neoantigen-loaded DC vaccine--ZSNeo-DC, when it was combined with the SOC therapy for nGBMs."
6. Page 8, Line 302:
   The following sentence has been changed: "This is a single-center,

single-arm phase 1b study to determine the safety and preliminary efficacy of ZSNeo-DC for treating nGBMs."

[2] How was the 10 M dose chosen?

T
h
e

d
o

Reference

1. Tanyi JL, Bobisse S, Ophir E, et al. Personalized cancer vaccine effectively mobilizes antitumor T cell immunity in ovarian cancer. *Sci Transl Med*. 2018;10(436):eaao5931. doi:10.1126/scitranslmed.aao5931

2. Fucikova J, Hensler M, Kasikova L, et al. An Autologous Dendritic Cell Vaccine Promotes Anticancer Immunity in Patients with Ovarian Cancer with Low Mutational Burden and Cold Tumors. *Clin Cancer Res*. 2022;28(14):3053-3065. doi:10.1158/1078-0432.CCR-21-4413

10^7 DC cells per injection was selected primarily based on experience from other DC trials. This dose has been validated in large-scale trials, such as

[3] How was the vaccine schedule chosen?

The dosing regimen was designed primarily based on the kinetics of dendritic cell (DC)-mediated immune responses and experience from other DC trials in nGBMs[1-4]. Upon administration, DC vaccines initiate an immune cascade by migrating to draining lymph nodes to prime naive T cells. This process triggers the expansion of antigen-specific T cells, which typically reaches its peak before entering a contraction phase, eventually leaving behind a pool of long-lived memory T cells[5]. The interval from T-cell priming to the peak of clonal expansion is approximately 7 - 10 days. Repeated vaccinations facilitate full expansion of antigen-specific T cells. Therefore, a three-injection protocol with a 7-10-day interval for immune induction was widely adopted in anti-glioma DC trials[1-4]. In this study, we adopted a schedule of three weekly injections (at Weeks 3, 4 and 5 post-radiotherapy) for immune induction.

We administered another two booster injections for immune consolidation at Weeks 8 and 12 respectively post-radiotherapy. The timing of the booster injections was planned to align with the initiation of TMZ maintenance

t
r
i

chemotherapy, two weeks after the start of 5-day TMZ treatment for each cycle. We anticipated this timing would be beneficial for triggering anti-tumor immune responses[6].

Moreover, given the aggressive nature of GBM and the limited window of clinical stability post-radiotherapy, we sought to capitalize on this "honeymoon period"—defined by favorable physical status, minimal chemotherapy-induced immune suppression, and radiation-mediated immune priming—by employing a condensed, intensive dosing regimen to optimize immunological and clinical outcomes.

Reference

1. Liau LM, Ashkan K, Brem S, et al. Association of Autologous Tumor Lysate-Loaded Dendritic Cell Vaccination With Extension of Survival Among Patients With Newly Diagnosed and Recurrent Glioblastoma: A Phase 3 Prospective Externally Controlled Cohort Trial. JAMA Oncol. 2023;9(1):112-121. doi:10.1001/jamaoncol.2022.5370
2. Inogés S, Tejada S, de Cerio AL, et al. A phase II trial of autologous dendritic cell vaccination and radiochemotherapy following fluorescence-guided surgery in newly diagnosed glioblastoma patients. J Transl Med. 2017;15(1):104. Published 2017 May 12. doi:10.1186/s12967-017-1202-z
3. Bota DA, Taylor TH, Piccioni DE, et al. Phase 2 study of AV-GBM-1 (a tumor-initiating cell targeted dendritic cell vaccine) in newly diagnosed Glioblastoma patients: safety and efficacy assessment. J Exp Clin Cancer Res. 2022;41(1):344. Published 2022 Dec 14. doi:10.1186/s13046-022-02552-6
4. Liau LM, Ashkan K, Tran DD, et al. First results on survival from a large Phase 3 clinical trial of an autologous dendritic cell vaccine in newly diagnosed glioblastoma. J Transl Med. 2018;16(1):142. Published 2018 May 29. doi:10.1186/s12967-018-1507-6
5. Blass E, Ott PA. Advances in the development of personalized neoantigen-based therapeutic cancer vaccines. Nat Rev Clin Oncol. 2021;18(4):215-229. doi:10.1038/s41571-020-00460-2
6. Latzer P, Zelba H, Battke F, et al. A real-world observation of patients with glioblastoma treated with a personalized peptide vaccine. Nat Commun. 2024;15(1):6870. Published 2024 Aug 11. doi:10.1038/s41467-024-51315-8

[4] How was i.d. route chosen?

The intradermal (i.d.) route was chosen as it is a standard approach in DC

therapy, facilitating efficient DC migration the draining lymph nodes[1,2].

Reference

1. Liau LM, Ashkan K, Brem S, et al. Association of Autologous Tumor Lysate-Loaded Dendritic Cell Vaccination With Extension of Survival Among Patients With Newly Diagnosed and Recurrent Glioblastoma: A Phase 3 Prospective Externally Controlled Cohort Trial. *JAMA Oncol*. 2023;9(1):112-121. doi:10.1001/jamaoncol.2022.5370
2. Inogés S, Tejada S, de Cerio AL, et al. A phase II trial of autologous dendritic cell vaccination and radiochemotherapy following fluorescence-guided surgery in newly diagnosed glioblastoma patients. J Transl Med. 2017;15(1):104. Published 2017 May 12. doi:10.1186/s12967-017-1202-z

[5] How was the 5 neoantigen threshold selected?

A threshold of 5 neoantigens was chosen based on prediction algorithm accuracy (~40%[1] and internal validation), ensuring more than two functional neoantigens per vaccine with high probability.

Reference

1. Bulik-Sullivan B, Busby J, Palmer CD, et al. Deep learning using tumor HLA peptide mass spectrometry datasets improves neoantigen identification. *Nat Biotechnol*. Published online December 17, 2018. doi:10.1038/nbt.4313

[6] Why was evaluability for efficacy based on treatment per protocol defined as 5 injections rather than 3 or 4, or even 1. Was this prespecified? 1 patient got 6 injections, 9 got 5 injections, 1 got 4 injections. All 11 were appropriately included in the safety analysis, but all 11 should have been included in the efficacy analysis as well. The one patient excluded was the oldest, and 1 of only 3 who had residual tumor following surgical resection; therefore, he would be expected to have a worse prognosis.

The "five-injection" criterion was defined primarily based on experience from other DC trials and DC manufacturing feasibility. Previous DC studies have shown that five injections are sufficient to induce an effective anti-tumor immune response[1,2].

10^7 cells for 5 injections is achievable in all the participants without repeat leukapheresis. Therefore, the "five-injection" criterion represents the optimal balance between maximizing dose intensity and avoiding burdensome repeat leukapheresis in GBM patients.

In addition, we entirely agree that patient R04 should be included in efficacy analysis. As suggested, we have made corresponding updates in the revised manuscript (please see Response #1).

Reference

1. Tanyi JL, Bobisse S, Ophir E, et al. Personalized cancer vaccine effectively mobilizes antitumor T cell immunity in ovarian cancer. *Sci Transl Med*. 2018;10(436):eaao5931. doi:10.1126/scitranslmed.aao5931

2. Fucikova J, Hensler M, Kasikova L, et al. An Autologous Dendritic Cell Vaccine Promotes Anticancer Immunity in Patients with Ovarian Cancer with Low Mutational Burden and Cold Tumors. *Clin Cancer Res*. 2022;28(14):3053-3065. doi:10.1158/1078-0432.CCR-21-4413

Treatment schedule: Lines 92 and 316 and Figure 2. It would be helpful to have the text and figure match. It appears this would be correct: A complete treatment course planned for injections at weeks 3, 4, 5, 8, and 12 post-concurrent RT/TMZ corresponding to weeks 17, 18, 19, 24, and 28 post-surgery. Additional injections were permitted at weeks 16, 20, and 24 post-concurrent RT/TMZ, corresponding to weeks 32, 36, and 40. The authors should explain why only 1 patient received more than 5 injections.

Response 8:

We sincerely thank you for your valuable suggestions and for identifying annotation inconsistencies in Figures. We have revised these sections, as detailed below.

(1) Description of treatment schedule

We have clarified the schedule according to our protocol and provided the primary rationale for the vaccine regimen.

In addition, given variable intervals between surgery and radiotherapy, we did not anchor our vaccine schedule to the surgical time-point.

Changes in manuscript:

1. Page 3, Line 93:

   The following sentence has been changed: “**Based on experience from benchmark DC trials in nGBMs**[5,7,10], **the standard** vaccination **schedule comprised** three **weekly doses for immune induction and at** least **two booster** doses **for immune consolidation. Specifically, ZSNeo-DC (1 × 10^7 DCs/dose) was administered intradermally near inguinal and axillary lymph nodes at weeks 3, 4, 5, 8, and 12 post-chemoradiotherapy (five-dose standard** course**). Up to three additional doses (weeks 16, 20, 24) were permitted if DCs were available. Multiple courses were allowed.**”

Reference

5. Liau LM, Ashkan K, Brem S, et al. Association of Autologous Tumor Lysate-Loaded Dendritic Cell Vaccination With Extension of Survival Among Patients With Newly Diagnosed and Recurrent Glioblastoma: A Phase 3 Prospective Externally Controlled Cohort Trial. JAMA Oncol 2023;9(1):112-121. DOI: 10.1001/jamaoncol.2022.5370.

7. Inoges S, Tejada S, de Cerio AL, et al. A phase II trial of autologous dendritic cell vaccination and radiochemotherapy following fluorescence-guided surgery in newly diagnosed glioblastoma patients. J Transl Med 2017;15(1):104. DOI: 10.1186/s12967-017-1202-z.

10. Bota DA, Taylor TH, Piccioni DE, et al. Phase 2 study of AV-GBM-1 (a tumor-initiating cell targeted dendritic cell vaccine) in newly diagnosed Glioblastoma patients: safety and efficacy assessment. J Exp Clin Cancer Res 2022;41(1):344. DOI: 10.1186/s13046-022-02552-6.

2. Page 8, Line 330:

   The following sentence has been changed: “ZSNeo-DC is administered intradermally **at a** dose **of 1 × 10^7** DCs **per injection, delivered** near the inguinal and axillary lymph nodes at weeks 3, 4, 5, 8, and 12 post-**chemoradiotherapy, comprising** a total of five doses **per** treatment course. **If sufficient DCs remain, up** to three additional doses **(at weeks 16, 20, and 24 post-chemoradiotherapy) may be administered** within **the same** course. **Multiple** treatment courses are permitted.”

(2) Annotation inconsistencies

We have updated Figure 2D to remove the inconsistencies. We have also updated Figure 1B to anchor the start of the vaccine schedule on the end of chemoradiotherapy.

(3) Explain why only 1 patient received more than 5 injections.

Due to constraints in DC manufacturing, as mentioned before (Response #7), only patient R01 had sufficient DCs for a sixth injection.

Discussion: Concerning feasibility, the authors should address the fact that after screening disqualified 22 potential subjects (withdrawal, poor KPS, not GBM etc), of the remaining 18, 6 (33%) had an insufficient number of antigens and therefore could not proceed.

Response 9:

We thank the reviewer for pointing out the feasibility issue of ZSNeo-DC therapy. A high exclusion rate is indeed a challenge for neoantigen-based DC immunotherapy. As suggested, we have highlighted this as a limitation of ZSNeo-DC therapy in the Discussion section of the revised manuscript.

Changes in manuscript:

1. Page 7, Line 287:
   The following sentence has been changed: "Additionally, **the 33.3% exclusion rate** due to **insufficient neoantigens may limit broader applicability** of **ZSNeo-DC therapy.**"

Data and Figures: There is an excellent amount of data presented. Probably the least helpful is the safety table, whereas extended Table 3 is critical for understanding prognostic baseline features, and should be included in the main manuscript.

Response 10: As suggested, we have moved Extended Data Table 3 to the main manuscript as Table 1.

References: These appear to have been generated by AI rather than curated by the authors. In many instances, text is not directly supported by the references, and conversely, the conclusions and findings of the references are not reflected in the text. As noted above, there are several key references describing clinical trials testing autologous DC loaded with autologous tumor antigens that are not cited or discussed.

Response 11:

All the references were curated by the authors rather than AI. We sincerely apologize for any reference inaccuracy. In this revision, we have performed a meticulous re-evaluation of references to ensure that each citation accurately supports the corresponding text. We found that one citation (reference 38, Page 6 Line 242 in the original manuscript) inaccurately supporting the correlation between tumor mutation burden and therapeutic efficacy of tumor vaccines. We had misused a reference regarding anti-PD1 therapy. We thank the review for identifying this error and have revised the reference to "Sun S, Liu L, Zhang J, et al. The role of neoantigens and tumor mutational burden in cancer immunotherapy: advances, mechanisms, and perspectives. *J Hematol Oncol*. 2025;18(1):84. Published 2025 Sep 2. doi:10.1186/s13045-025-01732-z".

Moreover, we have added the mentioned references in the revised manuscript, as detailed in our previous response (please see Response #5)

Reviewer #2 (Remarks to the Author):

This is a manuscript detailing the results of a phase I trial evaluating a personalized neoantigen-pulsed autologous dendritic cell vaccine as adjuvant treatment in patients with newly diagnosed GBM with a conclusion of safety of ZSNeo-DC and a reported "encouraging" efficacy signal.,

I would caution the authors regarding the use of "encouraging" and "potentially promising immunotherapy" in describing their ZSNeo-DC given the very small sample size in addition to the favorable baseline population they selected (example >90% resection of contrast enhancing disease).

Response 12:

We sincerely appreciate the reviewer's caution regarding our efficacy data interpretation. We acknowledge that the small sample size (n=11) and favorable baseline characteristics necessitate a conservative interpretation.

We have revised the descriptive language accordingly and expanded the Limitations section to address potential selection bias and cohort constraints, as detailed below.

Changes in manuscript:

1. Page 1, Line 34:

The following sentence has been changed: “Overall, ZSNeo-DC **exhibited favorable** safety **profile** and **preliminary efficacy signals** in nGBM, supporting phase 2 evaluation in expanded cohorts.”

2. Page 6, Line 233:

   The following sentence has been changed: “The survival data shows **an efficacy signal** of ZSNeo-DC therapy, as compared with the median PFS of 7-10 months commonly reported in nGBM patients receiving SOC treatment.”

3. Page 6, Line 239:

   The following sentence has been changed: “These observations **suggest** the **potential** immune **activity** of the vaccine. Together, these **exploratory findings provide a rationale for** further **investigation of ZSNeo-DC** in nGBMs.”

4. Page 7, Line 284:

   The following sentence has been changed: “As a pilot trial, **this study had a limited sample size, precluding statistically significant** correlations between NSIR and clinical outcomes **despite observable trends. Preliminary efficacy signals may be confounded by selection bias and post-progression management variability, requiring validation in randomized controlled trials.** Additionally, **the 33.3% exclusion rate due to insufficient neoantigens may limit broader applicability** of **ZSNeo-DC therapy.**”

5. Page 7, Line 294:

   The following sentence has been changed: “ZSNeo-DC vaccine, administered with the SOC treatment, was well- tolerated; **preliminary data suggest possible survival benefits in this small, selected nGBM cohort, warranting validation in larger trials.**”

Can the authors clarify why eligibility criteria required that patients have 90% or greater of their contrast enhancing resected?

Response 13:

“>=90% of contrast enhancement resected” is the threshold for “Near-Total Resection (NTR)” in GBM surgery. Utilizing this threshold allows for a relatively homogeneous patient population, thereby reducing the confounding impact by varying resection extents on survival outcomes. More importantly, patients under NTR represent a population with “low tumor burden” or

"minimal residual tumor volume" who are likely to benefit from tumor vaccine, as evidenced by multiple vaccine trials requiring "minimal residual tumor volume" for enrollment[1,2].

Reference

1. Inogés S, Tejada S, de Cerio AL, et al. A phase II trial of autologous dendritic cell vaccination and radiochemotherapy following fluorescence-guided surgery in newly diagnosed glioblastoma patients. *J Transl Med*. 2017;15(1):104. Published 2017 May 12. doi:10.1186/s12967-017-1202-z
2. Keskin DB, Anandappa AJ, Sun J, et al. Neoantigen vaccine generates intratumoral T cell responses in phase Ib glioblastoma trial. *Nature*. 2019;565(7738):234-239. doi:10.1038/s41586-018-0792-9

There is significant heterogeneity in the management of the patients in this small study which makes the survival data harder to interpret.

Response 14:

We agree that there is heterogeneity in patient management, particularly in salvage therapy owing to the lack of standard effective therapies after disease progression in GBM. As suggested, we have toned down efficacy claims in the revised manuscript and highlighted these limitations in the Discussion section.

Changes in manuscript:

1. Page 7, Line 284:
   The following sentence has been changed: "As a pilot trial, **this study had a limited sample size, precluding statistically significant** correlations between NSIR and clinical outcomes **despite observable trends. Preliminary efficacy signals may be confounded by selection bias and post-progression management variability, requiring validation in randomized controlled trials.**"

For patient R01, was the patient symptomatic? I'm unclear as to why the patient was treated with bevacizumab "for controlling pseudoprogression" which has implications on imaging interpretation unless the patient was symptomatic and requiring high dose steroids.

Response 15:

We sincerely appreciate your insightful comments.

The patient was indeed symptomatic at the peak of radiographic "pseudoprogression", manifesting as reduced walking distance and slightly decreased strength in the right lower limb compared to baseline. Given concerns regarding the potential adverse impact of steroids on efficacy and response to immunotherapy, the patient was administered bevacizumab rather than steroids to control these symptoms.

We apologize for omitting this detailed information in the original manuscript. We have accordingly revised this section, as follows.

Changes in manuscript:

1. Page 4, Line 146:
   The following sentence has been changed: "While the patient **initially** maintained physical activity **at** baseline, contrast-enhancement emerged surrounding the resection cavity **on** MRI at Week 42 (10 weeks after the last vaccination) and enlarged significantly at Week 74, **accompanied by reduced walking distance and slightly decreased strength in the right lower limb compared to baseline**. Without any treatment, the enhancement shrank **on** the subsequent MRI nine weeks later. Therefore, we suspected tumor pseudoprogression, a phenomenon commonly reported in anti-glioma vaccine studies[5,33], and administered six cycles of bevacizumab **to** control pseudoprogression **and related symptoms.**"

For patient R02, what was the justification to treat the patient with pembrolizumab and bevacizumab?

Response 16:

Patient R02 was treated with pembrolizumab and bevacizumab after tumor progression at the physician's discretion. The treatment was based on the rationale that anti-PD1/PD-L1 therapies might be effective at progression after tumor vaccine treatment[1] and that bevacizumab may enhance anti-tumor immune response[2] beyond standard treatment for GBM recurrence. Moreover, there are several reports showing potential synergy between DC vaccines and anti-PD1/PD-L1 therapies[3,4], as well as ongoing trials examining their safety and efficacy when administered concurrently in GBM[5,6].

Reference

1. Keskin DB, Anandappa AJ, Sun J, et al. Neoantigen vaccine generates intratumoral T cell responses in phase Ib glioblastoma trial. *Nature*. 2019;565(7738):234-239. doi:10.1038/s41586-018-0792-9
2. Allen E, Jabouille A, Rivera LB, et al. Combined antiangiogenic and anti-PD-L1 therapy stimulates tumor immunity through HEV formation. *Sci Transl Med*. 2017;9(385):eaak9679. doi:10.1126/scitranslmed.aak9679
3. Antonios JP, Soto H, Everson RG, et al. PD-1 blockade enhances the vaccination-induced immune response in glioma. *JCI Insight*. 2016;1(10):e87059. doi:10.1172/jci.insight.87059
4. Zhu P, Li SY, Ding J, et al. Combination immunotherapy of glioblastoma with dendritic cell cancer vaccines, anti-PD-1 and poly I:C. *J Pharm Anal*. 2023;13(6):616-624. doi:10.1016/j.jpha.2023.04.012
5. Ahluwalia MS, Peereboom DM, Ciolfi M, et al. Phase II study of pembrolizumab plus SurVaxM for glioblastoma at first recurrence. Journal of Clinical Oncology 2020;38(15_suppl):TPS2581-TPS2581. DOI: 10.1200/JCO.2020.38.15_suppl.TPS2581.
6. Yao Y, Huang X, Hu L, et al. Phase IIb randomized, blinded, controlled trial evaluating neoadjuvant PD-1 blockade combined with A2B5⁺ glioma stem-like cell lysate-loaded DC vaccine in recurrent glioblastoma (IDH1/2 -). Journal of Clinical Oncology 2024;42(16_suppl):2059-2059. DOI: 10.1200/JCO.2024.42.16_suppl.2059.

For better understanding by readers, we accordingly added this explanation in the revised manuscript, as detailed below.

Changes in manuscript:

1. Page 4, Line 158:
   The following sentence has been changed: “**Post-progression therapy was administered at the physician’s discretion. Based on the reported potential synergy between DC vaccines and anti-PD-1/PD-L1 therapies**[23,24,25], the patient was treated with pembrolizumab **in addition to standard** bevacizumab **therapy for recurrent GBM.**”
2. Page 7, Line 276:
   The following sentence has been added: “**Therefore, there are several reports showing potential synergy between DC vaccines and anti-PD1/PD-L1 therapies, as well as ongoing trials examining their safety and efficacy when administered concurrently in GBM.**”

What was the reason 10 patients withdrew consent?

Response 17:

We have checked the causes of patients withdrawing consent. We found that all these patients withdrew consent primarily due to inconvenience from strict travel restrictions for Covid-19 control in China at the pandemic peak between 2021 and 2022.

Why did so many patients receive bevacizumab after first progression? Were there no other lines of therapy to consider as an option (example CCNU)?

Response 18:

We sincerely appreciate your insightful question, which reminded us of information omitted in the original manuscript (Figure 2A and Table 1).

In this study, seven patients experienced disease progression, and their subsequent treatments were at the physician's discretion, based on molecular profiles, clinical performance status, and the rationale for combining tumor vaccine and anti-PD1 therapies (Response #16).

Specifically, patients R02, R06, R08, and R10 all had unmethylated MGMT promoter status. Given the aforementioned reason (Response #16) and concerns regarding possible unresponsiveness to alkylating agents such as CCNU in MGMT-unmethylated GBM, these patients were treated with anti-PD1 therapy in addition to bevacizumab. Subsequently, R06 and R08 had stable disease, and R02 even achieved complete remission that has been sustained to date. Patients R06 and R10 received a variety of salvage chemo/target therapies after second progression (detailed in revised Table 1). R08 died of brainstem hemorrhage caused by accidental trauma.

For patients R09 and R11, due to significant clinical deterioration at progression, intensive regimens such as CCNU were not considered; instead, they received single-agent bevacizumab for symptom palliation (R09) or TMZ plus cisplatin (discontinued after a single dose due to intolerable myelosuppression) combined with bevacizumab therapy (R11)

Patient R04 withdrew from the study protocol after the fourth vaccination due to inconvenience from strict travel restrictions during the Covid-19

pandemic and completed only the standard Stupp regimen.

We thank you again for your valuable comments. We have revised Figure 2A and Table 1 to provide detailed information regarding post-progression treatments.

For patient R10, why was progression not called until after treatment with bevacizumab?

Response 19:

Patient R10 developed symptomatic "trapped temporal horn syndrome" at Week 20 due to an enlarging contrast-enhancing lesion obstructing CSF drainage from the left temporal horn (Response Figure 1). Given that the enhancement appeared within 12 weeks after radiotherapy and particularly given the pathological diagnosis of biopsy samples obtained during ventriculostomy for treating "trapped temporal horn syndrome", pseudo-progression was diagnosed and the patient was administered bevacizumab to control associated edema and symptoms (headache, nausea, gait disturbance and cognitive impairment).

During bevacizumab treatment, a new contrast-enhancing lesion emerged in the left frontal region at Week 44, with significant clinical deterioration; this was reported as PD according to RANO criteria.

For better understanding by readers, we have added all surgical interventions after primary surgery to Figure 2A (R08 and R10). We sincerely thank the reviewer for this valuable comment.

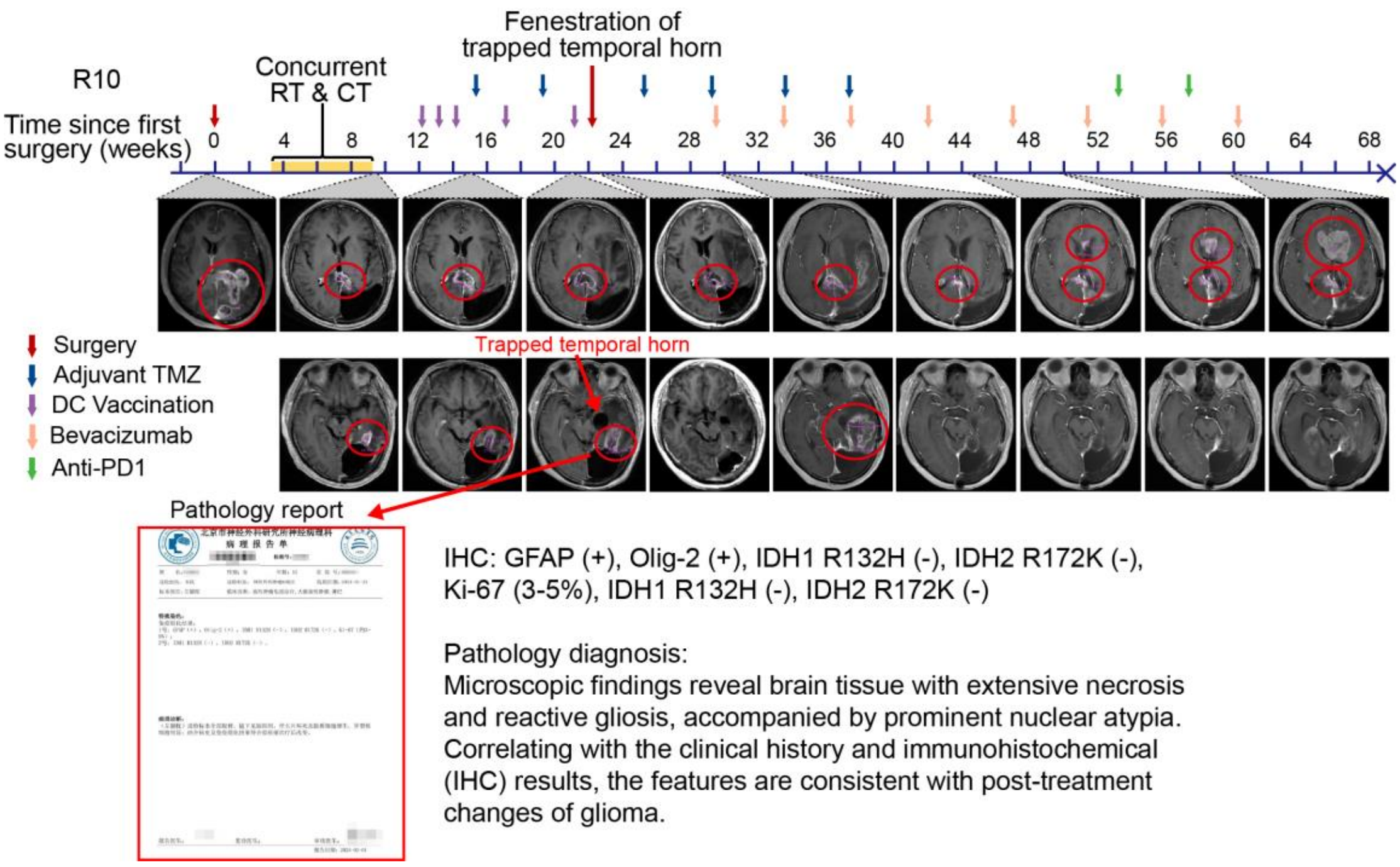

Response Figure 1. Treatment timelines and longitudinal imaging of patient R10

For the serial imaging, were any advanced imaging techniques used like perfusion and spectroscopy to aid in the review particularly those patients who had changes that were presumed to pseduoprogression?

Response 20:

We used MRI perfusion and/or PET scans to facilitate differentiation between pseudo-progression and true progression.

Two patients (R01 and R10) in this cohort were suspected of pseudo-progression during treatment. For patient R01, a PET-CT scan was planned to rule out true progression at initial lesion enlargement but failed due to travel restrictions by the COVID-19 pandemic. However, subsequent MRI confirmed the pseudo-progression diagnosis. Patient R10 had Perfusion-Weighted Imaging (PWI) at the time of suspected pseudo-progression, which showed an increased in relative cerebral blood volume (rCBV), raising suspicion for recurrence (Response Figure 2). However, subsequent pathological diagnosis supported pseudo-progression (Response #19).

In contrast, the diagnosis of tumor progression was facilitated by increased rCBV on PWI in patient R09, and elevated metabolic activity on PET in patients R06, R08 and R11 (Response Figure 2). Notably, in patient R11, PWI did not

show increased rCBV at tumor progression, whereas PET exhibited increased metabolic activity.

Collectively, tumor progression diagnosis was primarily based on RANO criteria, with advanced imaging techniques as ancillary approaches.

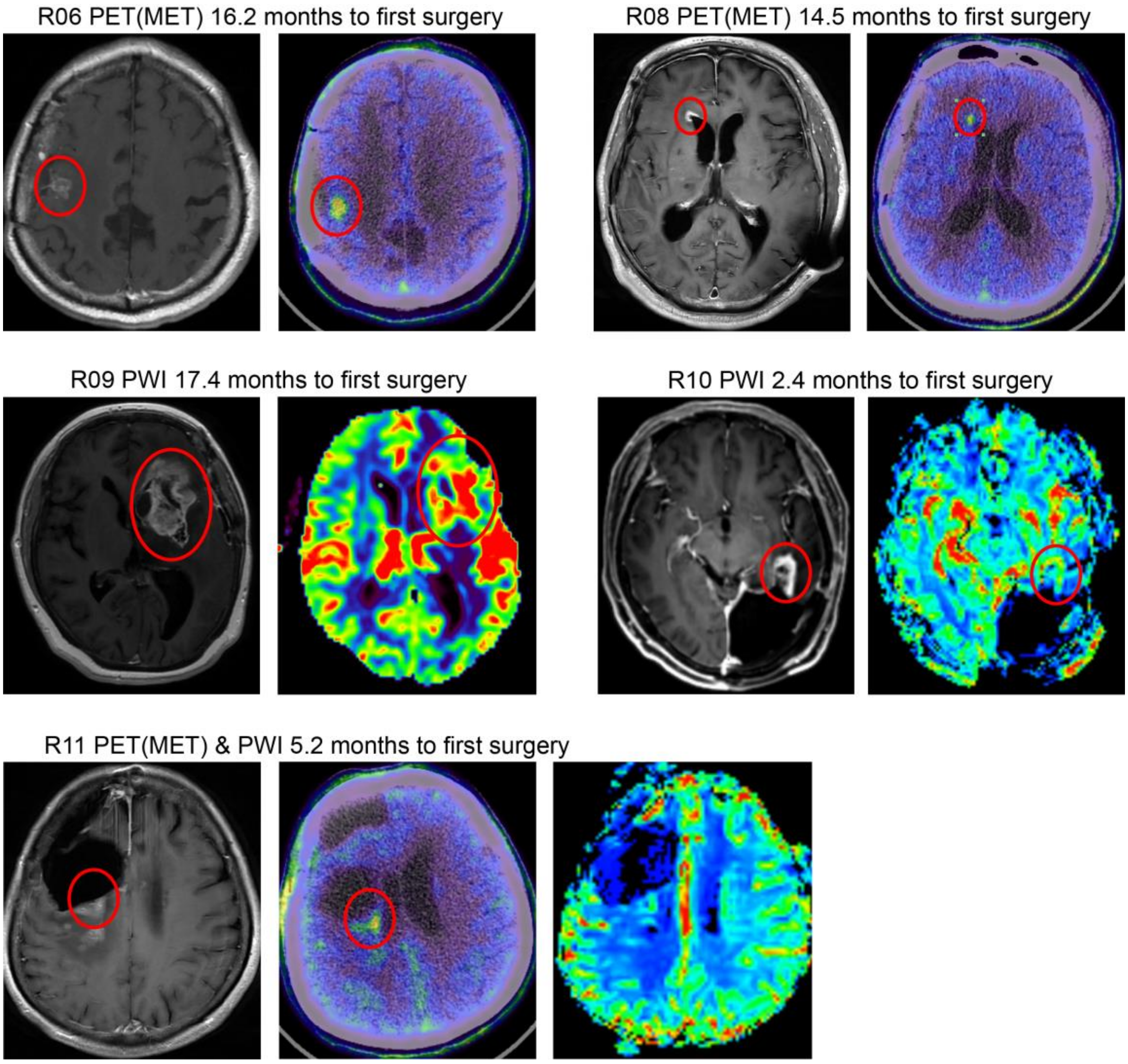

Response Figure 2. Advanced imaging of patients with suspected disease progression

Reviewer #3 (Remarks to the Author):

This Phase 1 study considers a novel personalized vaccine for treatment of newly-diagnosed glioblastoma, which has substantial unmet need for therapeutic advances since the current standard of care was established two decades ago. The sample size is small, but the results are promising and merit further investigation of the therapy in larger trials. My concerns

mostly relate to additional context needed to interpret the results in a way that makes clearer (a) how the safety and efficacy of the therapy relate to those of competing therapies, and (b) how further studies of this vaccine should be designed. With this additional context I think this paper can be an excellent contribution to the field.

Major Concerns:

1. To better characterize the analyzed patient population and its generalizability, please further describe (a) if there are any observed differences between patients who withdrew consent vs. those who consented, (b) any factors (clinical or technical) associated with having fewer than 5 predicted neoantigen peptides, and (c) the reason for the 1 manufacturing failure.

Response 21:

We thank the reviewer for these constructive comments.

(1) Differences between enrolled and consent-withdrawn patients

As suggested, we compared baseline characteristics between enrolled and consent-withdrawn patients (Response Table 1). No significant differences were observed, suggesting a low likelihood of bias toward better survival in enrolled patients.

(2) Factors associated with low number of predicted neoantigens

We also compared baseline characteristics between enrolled patients and those with < 5 predicted neoantigens (Response Table 1). Patients with < 5 predicted neoantigens exhibited better KPS and lower tumor mutation burden than enrolled patients. Therefore, we assumed that relatively low tumor mutation burden may partially contribute to the low number of predicted neoantigens in these screen-failed patients.

(3) Reason for the single manufacturing failure

We attempted vaccine manufacturing for the patient twice, but both attempts failed due to insufficient differentiation and maturation of monocyte-derived dendritic cells during culture.

Response Table 1. Comparisons of baseline characteristics between enrolled and screen-failed patients

Variable	Enrolled patients (n=11)	Consent-withdrawn patients (n=10)	P value	Patients with < 5 neoantigens (n=6)	P value
Age (ys), median (range)	55 (34, 67)	52.5 (42, 65)	0.864	55.5 (20, 67)	0.668
Sex (n)					
Male	6	7	0.659	4	1.000
Female	5	3		2	
Baseline KPS, median (range)	70 (60, 90)	70 (40, 80)	0.392	90 (80, 100)	0.004
Pre-operative volume (cm^3), median (range)	54.018 (16.555, 148.392)	71.886 (20.424, 99.483)	0.557	39.25 (30.836, 116.196)	0.660
Post-operative volume (cm^3), median (range)	0 (0, 1.482)	0 (0, 0)	0.094	0 (0, 11.889)	0.852
Extent of resection (%), median (range)	100 (97.17, 100)	100 (100, 100)	0.094	100 (71.822, 100)	0.852
MGMT (n)					

Methylated	3	3	1.000	4	0.406
Un-methylated	7	7		2	
TERT (n)					
Mutant	7	9	0.311	6	0.237
Wild-type	4	1		0	
Tumor mutation burden (mut/Mb), median (range)	2.65 (1.13, 803.96)	-	-	0.92 (0.48, 3.19)	0.018
CYT score	1.90 (1.04, 5.11)	-	-	1.73 (0.87, 3.96)	0.591

2. The abstract provides an incomplete and somewhat misleading summary of the AEs, especially the sentence "Treatment was well tolerated, with only two (18.2%) patients reporting grade-1/2 fever as treatment-related adverse events." which seems to imply that there were no other AEs aside from fever. In addition, in the Results and Discussion sections it would be helpful to comment in greater detail on how the observed AE rates compare specifically to those for other approved or investigational anti-glioma vaccines or competing therapies.

Response 22:

We thank the reviewer for pointing out the ambiguity in our description of adverse events (AEs). We have accordingly revised the Abstract to explicitly state this.

Furthermore, as suggested, we have compared our safety findings with benchmark DC trials in the Discussion section of the revised manuscript.

Changes in manuscript:

1. Page 1, Line 26:
   The following sentence has been changed: "Treatment was well tolerated, **with adverse events (AEs) predominantly grade 1 or 2;** only **two grade-1/2 febrile AEs were recorded** as treatment-related."
2. Page 6, Line 241:
   The following sentence has been added: "**ZSNeo-DC demonstrated a favorable safety profile, with only two grade 1/2 febrile TRAEs and no injection site reactions or fatigue commonly reported with DC therapy[10,24,40]. Consistent with previous reports[6,7,40], the most common AE was lymphocytopenia, recorded in 63.6% (7/11) of participants and likely related to standard therapy. Notably, no serious TRAEs were observed in this cohort, aligning with the extremely low incidence (0.3%-2.1%) reported in two large cohorts of DC therapy[6,24]. Together, the safety profile of ZSNeo-DC is comparable to that of these landmark studies, underscoring the safety advantage of DC therapy for GBM treatment.**"

3. Regarding the dosing, it would be helpful to explain (a) the rationale determining the dose amounts and schedule and any uncertainty regarding the potential superiority of other dosing schemes, (b) why no patients were

given more than 5 doses despite this being mentioned as a possibility in the protocol (i.e. why specifically did the subjects' tolerance and compliance patterns lead the investigator(s) to decide never to give more than 5 injections?).

Response 23:

We thank the reviewer for these insightful comments. Below is our point-by-point response to the specific concerns raised.

(1) Rationale for dose amount

The dose

Reference

1. Tanyi JL, Bobisse S, Ophir E, et al. Personalized cancer vaccine effectively mobilizes antitumor T cell immunity in ovarian cancer. *Sci Transl Med*. 2018;10(436):eaao5931. doi:10.1126/scitranslmed.aao5931

2. Fucikova J, Hensler M, Kasikova L, et al. An Autologous Dendritic Cell Vaccine Promotes Anticancer Immunity in Patients with Ovarian Cancer with Low Mutational Burden and Cold Tumors. *Clin Cancer Res*. 2022;28(14):3053-3065. doi:10.1158/1078-0432.CCR-21-4413

10^7 DC cells per injection was selected primarily based on experience from other DC trials. This dose has been validated in large-scale trials, such as

(2) Rationale for vaccine schedule

the VIABLE Phase 3

The dosing regimen was designed primarily based on the kinetics of dendritic cell (DC)-mediated immune responses and experience from other DC trials in nGBMs[1-4]. Upon administration, DC vaccines initiate an immune cascade by migrating to draining lymph nodes to prime naive T cells. This process triggers the expansion of antigen-specific T cells, which typically reaches its peak before entering a contraction phase, eventually leaving behind a pool of long-lived memory T cells[5]. The interval from T-cell priming to the peak of clonal expansion is approximately 7 - 10 days. Repeated vaccinations facilitate full expansion of antigen-specific T cells. Therefore, a three-injection protocol with a 7-10-day interval for immune induction was widely adopted in many anti-glioma DC trials[1-4]. In this study, we adopted a schedule of three weekly injections (at Weeks 3, 4 and 5 post-radiotherapy) for immune

induction.

We administered another two booster injections for immune consolidation at Weeks 8 and 12 respectively post-radiotherapy. The timing of the booster injections was planned to align with the initiation of TMZ maintenance chemotherapy, two weeks after the start of 5-day TMZ treatment for each cycle. We anticipated this timing would be beneficial for triggering anti-tumor immune responses[6].

Moreover, given the aggressive nature of GBM and the limited window of clinical stability post-radiotherapy, we sought to capitalize on this "honeymoon period" — defined by favorable physical status, minimal chemotherapy-induced immune suppression, and radiation-mediated immune priming—by employing a condensed, intensive dosing regimen to optimize immunological and clinical outcomes.

Reference

1. Liau LM, Ashkan K, Brem S, et al. Association of Autologous Tumor Lysate-Loaded Dendritic Cell Vaccination With Extension of Survival Among Patients With Newly Diagnosed and Recurrent Glioblastoma: A Phase 3 Prospective Externally Controlled Cohort Trial. JAMA Oncol. 2023;9(1):112-121. doi:10.1001/jamaoncol.2022.5370
2. Inogés S, Tejada S, de Cerio AL, et al. A phase II trial of autologous dendritic cell vaccination and radiochemotherapy following fluorescence-guided surgery in newly diagnosed glioblastoma patients. J Transl Med. 2017;15(1):104. Published 2017 May 12. doi:10.1186/s12967-017-1202-z
3. Bota DA, Taylor TH, Piccioni DE, et al. Phase 2 study of AV-GBM-1 (a tumor-initiating cell targeted dendritic cell vaccine) in newly diagnosed Glioblastoma patients: safety and efficacy assessment. J Exp Clin Cancer Res. 2022;41(1):344. Published 2022 Dec 14. doi:10.1186/s13046-022-02552-6
4. Liau LM, Ashkan K, Tran DD, et al. First results on survival from a large Phase 3 clinical trial of an autologous dendritic cell vaccine in newly diagnosed glioblastoma. J Transl Med. 2018;16(1):142. Published 2018 May 29. doi:10.1186/s12967-018-1507-6
5. Blass E, Ott PA. Advances in the development of personalized neoantigen-based therapeutic cancer vaccines. Nat Rev Clin Oncol. 2021;18(4):215-229. doi:10.1038/s41571-020-00460-2
6. Latzer P, Zelba H, Battke F, et al. A real-world observation of patients with glioblastoma treated with a personalized peptide vaccine. Nat Commun.

2024;15(1):6870. Published 2024 Aug 11. doi:10.1038/s41467-024-51315-8

(3) Rationale for "five-injection"

The "five-injection" criterion was defined primarily based on experience from other DC trials and DC manufacturing feasibility. Previous DC studies have shown that five injections are sufficient to induce effective an anti-tumor immune response[1,2].

With respect to DC manufacturing,

Reference

1. Tanyi JL, Bobisse S, Ophir E, et al. Personalized cancer vaccine effectively mobilizes antitumor T cell immunity in ovarian cancer. *Sci Transl Med*. 2018;10(436):eaao5931. doi:10.1126/scitranslmed.aao5931

2. Fucikova J, Hensler M, Kasikova L, et al. An Autologous Dendritic Cell Vaccine Promotes Anticancer Immunity in Patients with Ovarian Cancer with Low Mutational Burden and Cold Tumors. *Clin Cancer Res*. 2022;28(14):3053-3065. doi:10.1158/1078-0432.CCR-21-4413

(4) Why no additional injections or courses

Due to constraints in DC manufacturing, as mentioned before, only patient K01 had sufficient DCs for a sixth injection.

Regarding the statement "subjects' tolerance and compliance patterns lead the investigator(s) to decide never to give more than 5 injections" in the protocol, "tolerance" reflects our initial concerns regarding the tolerability of DC vaccine when combined with standard chemotherapy; "compliance patterns" reflects our initial concerns about the special timing of the COVID-19 pandemic, when most patients in the trial were enrolled and treated under strict travel restrictions in China that significantly impacted patient enrollment and return visits for repeated vaccine administrations. Thus, compliance patterns were particularly related to whether patients could comply with the required vaccine schedule under these strict travel restrictions.

4. Based on the Kaplan-Meier curves it appears that patients who experienced an "unfavorable" vaccine response (in terms of Gini coefficient and/or other metrics) had PFS rates comparable to published rates on the Stupp regimen SOC. It would be helpful to comment explicitly on this point specifically to contextualize survival differences by vaccine response.

Response 24:

We thank the reviewer for this insightful observation. We agree that contextualizing the survival of patients with an 'unfavorable' vaccine response relative to the historical SOC data would hint at the possibility of resistance to DC therapy in these patients. We have accordingly revised this part as follows.

Changes in manuscript:

Page 7, Line 260:

"By contrast, patients without these favorable immunological profiles exhibited survival outcomes, on some occasions, comparable to those reported for the standard Stupp regimen[5,41,42], suggesting potential resistance to ZSNeo-DC therapy."

5. I understand the sample size is very limited to answer this question, but with that in mind was there any evidence that MGMT methylation status or other tumor genomic markers were associated with vaccine response?

Response 25:

We appreciate the reviewer's insightful comment regarding the potential genomic correlates of the vaccine response. As suggested, we attempted to investigate the association between tumor molecular features and the magnitude of neoantigen-specific immune response (NSIR) (Response Figure 3). As a result, we observed that patients with MGMT-unmethylated or TERT-wildtype tumors showed a trend toward higher NSIR, while this trend did not translate to extended PFS (Extended Table 3). The mechanisms underlying these correlations are intriguing. However, given the sample size noted by the reviewer, these findings require further validation in subsequent larger trials.

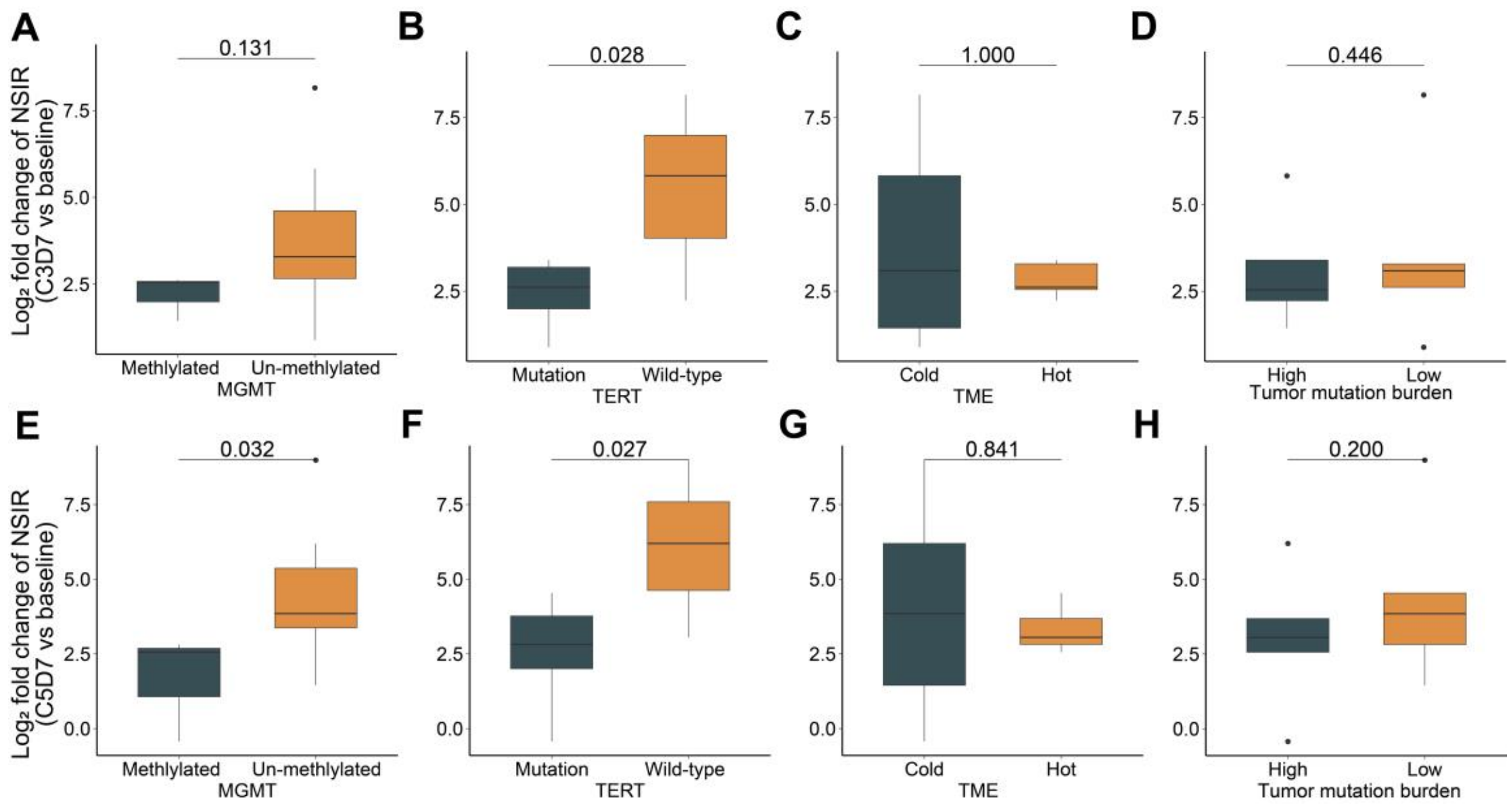

Response Figure 3. Association between tumor genomic features and neoantigen-specific immune response changes after ZSNeo-DC therapy.

(A, E) Comparison of neoantigen-specific immune response (NSIR) fold changes between patients with MGMT-methylated and unmethylated tumors.

(B, F) Comparison of NSIR fold changes between patients with TERT-promoter mutation and wild-type tumors.

(C, G) Comparison of NSIR fold changes between patients with cold and hot tumor microenvironment (TME).

(D, H) Comparison of NSIR fold changes between patients with high (≥ median) and low (<median) tumor mutational burden.

Two-sided t-tests were applied to assess statistical significance. The median and interquartile range with whiskers extending to the minimum and maximum values are depicted.

Minor concerns:

1. On page 3 "stupp's" should be capitalized.

Response 26:

We thank the reviewer for this careful observation. We have corrected the capitalization of 'Stupp' s' throughout the manuscript, including on Page 3.

Changes in manuscript:

Page 3, Line 117:

"In combination with the vaccination, all patients received the standard **Stupp's** regimen of radio-chemotherapy[28], and one patient (R05) concurrently had the TTF therapy, which has also constituted a standard treatment for nGBM[2]."

2. On page 7 TME acronym (I assume tumor microenvironment) should be defined.

Response 27:

We appreciate the reviewer's suggestion. The acronym 'TME' has now been defined as 'tumor microenvironment' at its first appearance in the revised manuscript.

Changes in manuscript:

Page 7, Line 273:

"GBM is notorious for its "cold" and inhibitory **tumor microenvironment (TME)**[49,50], leading to its resistance to immune checkpoint blockade (ICB) therapies in several large-scale trials[47]."

Response to Reviewers

Manuscript title: Personalized neoantigen-pulsed autologous dendritic cells in newly-diagnosed glioblastoma: a phase Ib trial.

Manuscript ID: NCOMMS-25-101455B

We thank the reviewers and editor for their constructive comments.

We have carefully revised the manuscript and addressed all concerns.

REVIEWERS' COMMENTS

Reviewer #1 (Remarks to the Author):

Nature Communications. (revised title): Zhang Y, Chi X, Liu K et al. Personalized neoantigen-pulsed autologous dendritic cells vaccine as an adjuvant treatment for patients with newly-diagnosed glioblastoma multiforme: a phase IB trial.

General Comments:

The authors have provided a revised manuscript that adequately addresses most of the issues previously raised by reviewers. This includes citing several other relevant GBM vaccine studies that were previously published.

Specific comments;

1.Line 55: Please exchange the existing reference 8 (Erhart F, Buchroithner J Reitermeir R et al. with their other 2018 reference: Buchroithner J, Erhart F, Pichler J, et al. Audencel immunotherapy based on dendritic cells has no effect on overall and progression-free survival in newly diagnosed glioblastoma: A phase II randomized trial. Cancers (Basel). 2018;10:372. This is the publication that actually describes the randomized clinical trial and its results. The Erhart reference, which is focused on the immunologic correlations, is appropriate for the current 8 on line 254.

Response 1:

We thank the reviewer for this constructive suggestion. We carefully examined the current reference 8 (Erhart F, Buchroithner J, Reitermaier R, et al. Immunological analysis of phase II glioblastoma dendritic cell vaccine (Audencel) trial: immune system characteristics influence outcome and Audencel up-regulates Th1-related immunovariables. Acta Neuropathol Commun 2018;6(1):135. DOI: 10.1186/s40478-018-0621-2.). Upon revision, we recognize that this study primarily focuses on immunological correlates rather than clinical outcomes. Therefore, to ensure conceptual accuracy and consistency within the context of our manuscript, we have revised the corresponding statement to appropriately reflect immunological efficacy rather than clinical benefit.

Regarding the replacement reference (Buchroithner J, Erhart F, Pichler J, et al. Audencel immunotherapy based on dendritic cells has no effect on overall and progression-free survival in newly diagnosed glioblastoma: A phase II randomized trial. Cancers (Basel). 2018;10:372.), it seems inappropriate to cite it in this section, since it primarily reports no survival benefits. However, this publication describes one of the few randomized clinical trials for anti-glioma vaccines and demonstrates a favorable safety profile of the investigated DC therapy. Therefore, we have cited it in the second paragraph of the Discussion section as reference 40 (Page 6, Line 244).

Changes in manuscript:

Page 2, Line 53:
The following sentence has been changed: “These endeavors have yielded encouraging **therapeutic efficacy** in treating this deadly brain cancer[5, 6, 7, 8, 9, 10].”

Reference
5. Liau LM, et al. Association of Autologous Tumor Lysate-Loaded Dendritic Cell Vaccination With Extension of Survival Among Patients With Newly Diagnosed and Recurrent Glioblastoma: A Phase 3 Prospective Externally Controlled Cohort Trial. JAMA Oncol 9, 112-121 (2023).
6. Liau LM, et al. First results on survival from a large Phase 3 clinical trial of an autologous dendritic cell vaccine in newly diagnosed glioblastoma. J Transl Med 16, 142 (2018).
7. Inoges S, et al. A phase II trial of autologous dendritic cell

vaccination and radiochemotherapy following fluorescence-guided surgery in newly diagnosed glioblastoma patients. J Transl Med 15, 104 (2017).
8. Erhart F, et al. Immunological analysis of phase II glioblastoma dendritic cell vaccine (Audencel) trial: immune system characteristics influence outcome and Audencel up-regulates Th1-related immunovariables. Acta Neuropathol Commun 6, 135 (2018).
9. Ridolfi L, et al. First step results from a phase II study of a dendritic cell vaccine in glioblastoma patients (CombiG-vax). Front Immunol 15, 1404861 (2024).
10. Bota DA, et al. Phase 2 study of AV-GBM-1 (a tumor-initiating cell targeted dendritic cell vaccine) in newly diagnosed Glioblastoma patients: safety and efficacy assessment. J Exp Clin Cancer Res 41, 344 (2022).

2. Lines 66-67 are referring to neoantigen vaccines that have been tested in glioma patients. References 23 and 24 appear to be appropriate, but reference 25 (Grassl N et al) describes treatment with H3K27M-specific long peptide vaccine (H3K27M-vac) and is being targeted as a shared tumor-associated antigen rather than patient-specific neoantigens. It is not appropriate to characterize the study by Latzer P et al as "pilot study."

Response 2:

We thank the reviewer for this important clarification. We agree that the study by Grassl N et al. describes an H3K27M-specific long peptide vaccine targeting a shared antigen rather than patient-specific neoantigens and therefore should not be cited in this context. Accordingly, we have removed the inappropriate citation in the manuscript.

Additionally, we have also removed the "pilot" in the revised manuscript, and revised the corresponding sentence as "Based on them, several studies on personalized neoantigen peptide vaccines have demonstrated promising results in triggering intense tumor-specific T cell responses and thus extending survival in glioma patients[23, 24]." (Line 67, Page 2)

3. Lines 225-226. The first line of the discussion should be revised for accuracy and clarity. DCs themselves do not constitute a vaccine. DC vaccines have not been tested in low-grade gliomas. The terms "cancer vaccines" and "tumor vaccines" imply anti-tumor or anti-cancer activity.

For example, one might say “Many clinical trials have been conducted utilizing dendritic cell vaccines to treat malignant gliomas.” References

Response 3:

We thank you for this helpful comment. We have revised this sentence according to your suggestion,

Changes in manuscript:

Page 6, Line 246:
The sentence has been revised as: “**Many clinical trials have been conducted utilizing DC vaccines to treat malignant gliomas**[5, 6, 7, 8, 10, 14, 18, 41, 42].”

4. Discussion Lines 226,242,246. In previous comments, the reviewer erroneously advised that the Latzer P publication from 2024 (reference 24) described primary GBM patients treated with autologous DC and neoantigens. In fact, as clearly described in that manuscript, these were not dendritic cell vaccines; their vaccines consisted of patient-specific neoantigen peptides (range of 6 to 24) that were injected with adjuvant GM-CSF. Therefore, reference 24 should be removed from the superscripted references on lines 226, 242, and 246.

Response 4:

We thank the reviewer for reminding us of this citing error. Upon careful re-evaluation of the cited literature, we confirm that the study by Latzer P et al. employed patient-specific neoantigen peptides administered with GM-CSF as an adjuvant, instead of dendritic cell (DC)-based vaccines. Therefore, its inclusion in the context of DC vaccine studies is inappropriate. We sincerely appreciate this helpful comment.

Accordingly, we have removed reference 24 from the superscripted citations on Lines 226, 242, and 246 in the revised manuscript. In addition, we have replaced the citation on Line 246 with a more appropriate reference (Bota et.al. 2022) and revised the corresponding text.

Changes in manuscript:

Page 6, Line 243:
The following sentence has been revised as: “Notably, no serious TRAEs

were observed in this cohort, aligning with the extremely low incidence (**0-0.3%**) reported in large cohorts of DC therapy[6, 10, 40]."

Reference
6. Liau LM, et al. First results on survival from a large Phase 3 clinical trial of an autologous dendritic cell vaccine in newly diagnosed glioblastoma. J Transl Med 16, 142 (2018).
10. Bota DA, et al. Phase 2 study of AV-GBM-1 (a tumor-initiating cell targeted dendritic cell vaccine) in newly diagnosed Glioblastoma patients: safety and efficacy assessment. J Exp Clin Cancer Res 41, 344 (2022).
40. Buchroithner J, et al. Audencel Immunotherapy Based on Dendritic Cells Has No Effect on Overall and Progression-Free Survival in Newly Diagnosed Glioblastoma: A Phase II Randomized Trial. Cancers (Basel) 10, (2018).

5. Discussion. Latzer et al. previously reported encouraging clinical outcomes with no safety concerns in a neoantigen GBM trial that was a much larger clinical study of GBM personalized neoantigen vaccines than described in the current report (70 vs 11 primary GBM patients). The results of that report should be briefly summarized in the discussion. Please comment that despite the theoretical advantages of antigen delivery by DC, comparable results might be obtained with other neoantigen vaccine strategies.

Response 5:

We have placed a brief summary of the study by Latzer P et al. in the Discussion section of the revised manuscript.

Changes in manuscript:

Page 6, Line 246:
The following paragraph has been added as: "Many clinical trials have been conducted utilizing DC vaccines to treat malignant gliomas[5, 6, 7, 8, 10, 14, 18, 41, 42]. Current DC-vaccine strategies for gliomas include pulsing DCs with tumor lysate, TAAs and VAs. Neoantigen-loaded DC vaccines are theorized to elicit a stronger and more specific anti-tumor immune response. This study represents the first to show the promise of the DC platform to provide neoantigen-based immunotherapies against gliomas. Nevertheless, a recent trial by Latzer P et al. involving 70 patients with nGBM also demonstrated encouraging clinical outcomes and a favorable safety profile with

personalized neoantigen peptide vaccines[24], suggesting that comparable clinical benefits may be achieved with alternative neoantigen vaccine strategies."

Reference

5. Liau LM, et al. Association of Autologous Tumor Lysate-Loaded Dendritic Cell Vaccination With Extension of Survival Among Patients With Newly Diagnosed and Recurrent Glioblastoma: A Phase 3 Prospective Externally Controlled Cohort Trial. JAMA Oncol 9, 112-121 (2023).

6. Liau LM, et al. First results on survival from a large Phase 3 clinical trial of an autologous dendritic cell vaccine in newly diagnosed glioblastoma. J Transl Med 16, 142 (2018).

7. Inoges S, et al. A phase II trial of autologous dendritic cell vaccination and radiochemotherapy following fluorescence-guided surgery in newly diagnosed glioblastoma patients. J Transl Med 15, 104 (2017).

8. Erhart F, et al. Immunological analysis of phase II glioblastoma dendritic cell vaccine (Audencel) trial: immune system characteristics influence outcome and Audencel up-regulates Th1-related immunovariables. Acta Neuropathol Commun 6, 135 (2018).

10. Bota DA, et al. Phase 2 study of AV-GBM-1 (a tumor-initiating cell targeted dendritic cell vaccine) in newly diagnosed Glioblastoma patients: safety and efficacy assessment. J Exp Clin Cancer Res 41, 344 (2022).

14. Wen PY, et al. A Randomized Double-Blind Placebo-Controlled Phase II Trial of Dendritic Cell Vaccine ICT-107 in Newly Diagnosed Patients with Glioblastoma. Clin Cancer Res 25, 5799-5807 (2019).

18. Batich KA, Mitchell DA, Healy P, Herndon JE, 2nd, Sampson JH. Once, Twice, Three Times a Finding: Reproducibility of Dendritic Cell Vaccine Trials Targeting Cytomegalovirus in Glioblastoma. Clin Cancer Res 26, 5297-5303 (2020).

24. Latzer P, et al. A real-world observation of patients with glioblastoma treated with a personalized peptide vaccine. Nat Commun 15, 6870 (2024).

41. Wong CE, et al. Dendritic cell vaccine for glioblastoma: an updated meta-analysis and trial sequential analysis. J Neurooncol 170, 253-263 (2024).

42. Yao Y, et al. Molecular subgroups and B7-H4 expression levels predict responses to dendritic cell vaccines in glioblastoma: an exploratory randomized phase II clinical trial. Cancer Immunol Immunother 67, 1777-1788 (2018).

6. Discussion. The authors may want to note that variations in dendritic cell manufacturing may account for some of the differences between studies, and that the DC in the other studies cited in this article did not load antigens in the presence of anti-PDL-1 and a CD40 agonist, and not all used cytokines to induce maturation.

Response 6:

We thank the reviewer for the constructive comment. We have highlighted these differences in the Discussion section of the revised manuscript.

Changes in manuscript:

Page 6, Line 253:
The following sentences have been added in the revised manuscript:
"Besides the neoantigen design, ZSNeo-DC manufacturing adopts cytokine-driven maturation and utilizes PD-L1 blockade and CD40 agonism to enhance DC functions; these procedures are not routinely used in the manufacturing of other DC vaccines. The impact of these manufacturing variations on clinical and immune responses warrants further investigations."

7. References. The authors should use the preferred style of the journal rather than copying the citations from PubMed or other sources. For example, some references have capitalization of words other than articles or prepositions in the titles while others are all lower case.

Response 7:

We sincerely appreciate your help. We have revised all references to ensure consistency with the journal's preferred citation style, including the standardization of title capitalization and overall formatting.

Reviewer #3 (Remarks to the Author):

I appreciate the author's responsiveness to my previous comments and I am mostly satisfied with the revision. I only have two further comments which should be easy to address.

1. Response Table 1 is interesting and relevant to the interpretation of the trial results. It should be included as an Extended Data Table and discussed briefly in the Results section (both the lack of apparent differences between the enrolled/consent-withdrawn patients and the factors associated with low number of predicted neoantigents).

Response 1:

We thank the reviewer for this insightful suggestion. We agree that the information in Response Table 1 is important for interpreting the trial results. Accordingly, we have included this table in the revised manuscript as Table S3 and added a brief description in the Results section.

Changes in manuscript:

Page 3, Line 122:
The following paragraph has been included in the revised manuscript:
"There was no significant difference in baseline characteristics between the treated and consent-withdrawn patients, whereas those patients excluded due to a low number of predicted neoantigens exhibited better baseline KPS and lower tumor mutation burden than the treated patients (Table S3). Therefore, the low tumor mutation burden may partially contribute to ineligibility for ZSNeo-DC therapy."

2. To my understanding, there were no manuscript changes in my previous comment 3 about the dosing rationale. Please include in the manuscript a brief discussion of these issues and some of the references (if you're past journal limits on manuscript length, this could be included as Supplementary Material).

Response 2:

We thank the reviewer for this suggestion. We have now added a brief discussion of the dosing rationale in the Methods section of the revised manuscript.

Changes in manuscript:

Page 8, Line 339:
The following sentences have been added: "The dose of 1×10^7 cells[54] and the dosing scheme[5, 10] were designed primarily based on the kinetics of DC-mediated immune responses and experience from other DC trials, with three

weekly induction doses for full expansion of antigen-specific T cells, and two subsequent booster doses for immune consolidation. The timing of the booster doses was set at two weeks after the start of the 5-day TMZ maintenance chemotherapy to further enhance anti-tumor immune responses[24]."

Reference

5. Liau LM, Ashkan K, Brem S, et al. Association of Autologous Tumor Lysate-Loaded Dendritic Cell Vaccination With Extension of Survival Among Patients With Newly Diagnosed and Recurrent Glioblastoma: A Phase 3 Prospective Externally Controlled Cohort Trial. JAMA Oncol 2023;9(1):112-121. DOI: 10.1001/jamaoncol.2022.5370.

10. Bota DA, Taylor TH, Piccioni DE, et al. Phase 2 study of AV-GBM-1 (a tumor-initiating cell targeted dendritic cell vaccine) in newly diagnosed Glioblastoma patients: safety and efficacy assessment. J Exp Clin Cancer Res 2022;41(1):344. DOI: 10.1186/s13046-022-02552-6.

24. Latzer P, Zelba H, Battke F, et al. A real-world observation of patients with glioblastoma treated with a personalized peptide vaccine. Nat Commun 2024;15(1):6870. DOI: 10.1038/s41467-024-51315-8.

54. Vogelzang NJ, et al. Efficacy and Safety of Autologous Dendritic Cell-Based Immunotherapy, Docetaxel, and Prednisone vs Placebo in Patients With Metastatic Castration-Resistant Prostate Cancer: The VIABLE Phase 3 Randomized Clinical Trial. JAMA Oncol 8, 546-552 (2022).
